# Anticancer and Anti-Inflammatory Activities of Some New Pyrazolo[3,4-*b*]pyrazines

**DOI:** 10.3390/molecules23102657

**Published:** 2018-10-16

**Authors:** Hussein El-Kashef, Talaat El-Emary, Pierre Verhaeghe, Patrice Vanelle, Maha Samy

**Affiliations:** 1 Chemistry Department, Faculty of Science, Assiut University, 71516 Assiut, Egypt; emarytalaat@yahoo.com (T.E.-E.); maha11292@yahoo.com (M.S.); 2 LCC-CNRS Université de Toulouse, CNRS, UPS, 205 Route de Narbonne, 3107 Toulouse, France; pierre.verhaeghe@lcc-toulouse.fr; 3 Aix-Marseille Université, CNRS, Institut de Chimie Radicalaire ICR, UMR 7273, Laboratoire de Pharmaco-Chimie Radicalaire, Faculté de Pharmacie, 27 Boulevard Jean Moulin CS 30064, 13385 Marseille CEDEX 05, France; patrice.vanelle@univ-amu.fr

**Keywords:** pyrazolo[3,4-*b*]pyrazines, *o*-aminonitrosopyrazole, anticancer, anti-inflammatory, anti-breast cancer

## Abstract

New derivatives of pyrazolo[3,4-*b*]pyrazines and related heterocycles were synthesized using 5-amino-3-methyl-4-nitroso-1-phenyl-pyrazole (**1**) as a starting material. The 5-acetyl derivative **15** was shown to be a useful key intermediate for the synthesis of several derivatives of pyrazolopyrazines. Some of the prepared compounds were evaluated for their anti-inflammatory and anti-breast cancer MCF-7 cell line activities. SAR study showed that compounds **15** and **29** exhibited remarkable anti-inflammatory activity, where **15** showed the same activity as that of the reference drug indomethacin. On the other hand, compounds **25i**, **25j** showed very significant inhibitory activity (*p* < 0.001) against MCF-7 breast cancer cell line.

## 1. Introduction

Although pyrazolo[3,4-*b*]pyrazines are not highly sited in the literature, they proved to be an interesting class of pyrazolopyrazine heterocyles. Therapeutic importance has been reported for these heterocycles, such as their use for the treatment and/or prevention of a wide variety of diseases related to adenosine receptors, depression, anxiety, Parkinson’s disease, pain, dementia, heart failure, and cerebrovascular disease [1,2,3].

They are also used as therapeutic agents for periodontosis, hypercalcemia, osteoporosis, rheumatoid arthritis, Paget’s disease, and as bone metabolism improvers [4]. Some reports indicated their use as blood platelet aggregation inhibitors [5], anti-inflammatories [6], in controlling herbicides [7], and anticancer agents with low toxicity [8,9]. In the domain of dye chemistry, they are used as fluorescent [10] and disperse dyes [11]. Certain derivatives were reported to possess antiviral, antineoplastic, antiparasitic, and anti-fungal properties [12,13,14,15]. Others showed anticonvulsant [16] and antibacterial activities [17,18]. Certain derivatives are useful for the treatment of hematologic diseases [19], also for the prophylaxis and treatment of protein kinase-mediated diseases, including inflammation and other related diseases. They are also used for the treatment of p38 map kinase-mediated diseases including rheumatoid arthritis, psoriasis, chronic obstructive pulmonary disease, pain, and other inflammatory disorders [20]. A microwave-assisted synthesis of fused pyrazolo[3,4-*b*]pyrazines *via* the reaction of *o*-aminonitrosopyrazoles with cyclic *β*-diketones was also reported [21].

In continuation with our interest in the synthesis of pyrazolo[3,4-*b*]pyrazins [5,12,13,16,17,18], we report herein the synthesis of other derivatives and related heterocycles. Certain newly synthesized derivatives were screened for their anti-inflammatory and and anti-breast cancer MCF-7 cell line activities.

## 2. Results

### 2.1. Chemistry

**Scheme 1:**
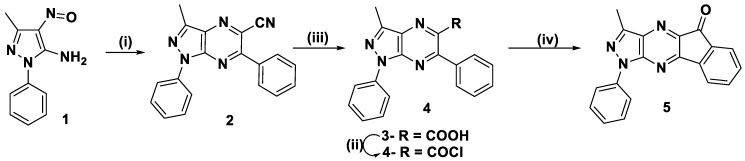
Synthesis of compound **5**. Reagents and conditions: (i) Benzoylacetonitrile, pyridine, reflux 3 h; (ii) NaOH 20%, reflux 6 h; (iii) SOCl_2_, reflux 2 h; and (iv) AlCl_3_, CS_2_, reflux 6 h.

In a preceding paper of our group [18], we have described the first synthesis of 3-methyl-1-phenyl-1*H*-indeno[2,1-*e*]pyrazolo[3,4-*b*]pyrazin-5-one (**5**). The synthetic route for this compound involved the use of *o*-aminonitrosopyrazole **1** as a starting material, which was reacted with the active methylene benzoylacetonitrile to give **2**. Hydrolysis of the cyano group of the latter compound gave the carboxylic acid **3**, which was converted into the acid chloride **4**, followed by intramolecular Friedel-Crafts cyclization giving **5** (Scheme 1).

**Scheme 2:**
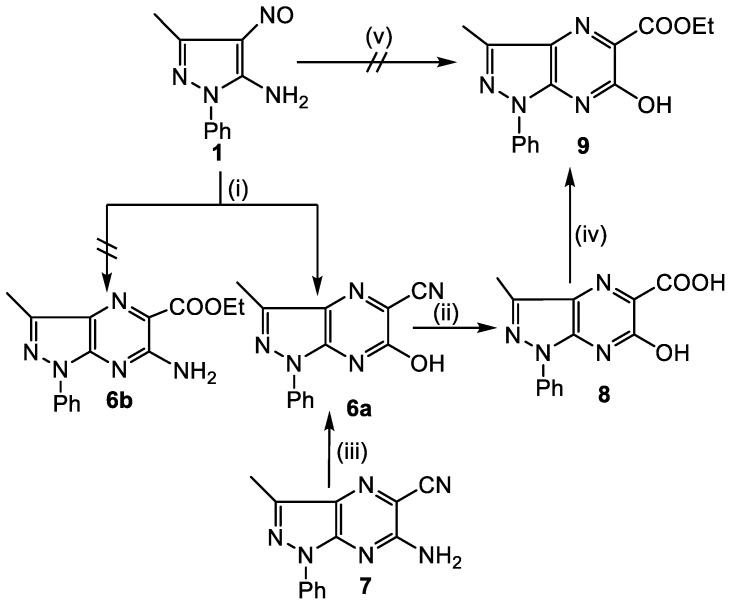
Synthesis of compounds **6**–**9**. Reagents and conditions: (i) CH_2_(CN)(COOC_2_H_5_), pyridine, reflux overnight; (ii) NaNO_2_, H_2_SO_4_, room temperature (rt) at 0 °C; (iii) NaOH 20%, reflux 5 h; (iv) EtOH, HCl, reflux 6 h; and (v) CH_2_(COOC_2_H_5_)_2_, pyridine, reflux overnight.

In the present work, when *o*-aminonitrosopyrazole **1** was reacted with ethyl cyanoacetate in refluxing pyridine, the expected reaction product could be either **6a** or **6b** (Scheme 2). The structure **6b** was immediately ruled out by examining the ir spectrum of the product, which showed no bands corresponding to -NH_2_ and -COOEt groups. However, the spectrum showed two bands at 3197 and 2254 cm^−1^ corresponding to ν OH and ν CN, respectively confirming the structure **6a**. Further confirmation of this structure was obtained from the ^1^H-NMR spectral analysis which showed, in addition to the phenyl protons, two characteristic signals assigned to CH_3_ and OH protons at 2.51 and 8.43 ppm, respectively. Alternatively, compound **6a** was obtained through unequivocal synthesis *via* diazotization of the amino group of the derivative **7** [13], followed by decomposition of the resulting diazonium salt. The alkaline hydrolysis of **6a** gave the hydroxycarboxylic acid **8**, which was esterified in refluxing absolute ethanol in the presence of concentrated H_2_SO_4_ to give the corresponding hydroxyester **9**. Contrary to the work of Rangnekar et al. [22], all attempts to prepare the latter compound **9** directly through the reaction of **1** with diethylmalonate under various conditions were unsuccessful (Scheme 2).

**Scheme 3:**
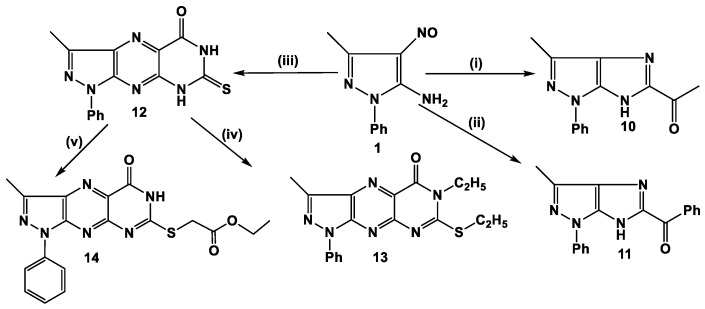
Synthesis of compounds **10**–**14**. Reagents and conditions: (i) CH_3_COCH_2_Cl, pyridine, reflux overnight; (ii) PhCOCH_2_Br, pyridine, reflux overnight; (iii) thiobarbituric acid, pyridine, reflux overnight; (iv) C_2_H_5_I, DMF, rt 5 h; and (v) ClCH_2_COOC_2_H_5_, DMF, rt 5 h.

On the other hand, the interaction of **1** with *α*-haloketones such as chloroacetone and phenacyl bromide gave the corresponding imidazo[4,5-*c*]pyrazole derivative **10** and **11** respectively. When the aminonitrosopyrazole **1** was reacted with thiobarbituric acid in refluxing pyridine, the reaction product was identified as 3-methyl-1-phenyl-7-thioxo-7,8-dihydro-1*H*-pyrazolo[4,3-*g*]pteridin- 5(6*H*)-one (**12**). Alkylation of **12** with excess ethyl iodide in DMF in the presence of anhydrous K_2_CO_3_ yielded 6-ethyl-7-(ethylthio)-3-methyl-1-phenyl-1*H*-pyrazolo[4,3-*g*]pteridin-5(6*H*)-one (**13**), however when **12** was interacted with one mole of ethyl chloroacetate, the ethyl mercaptoacetate derivative **14** was obtained (Scheme 3).

**Scheme 4:**
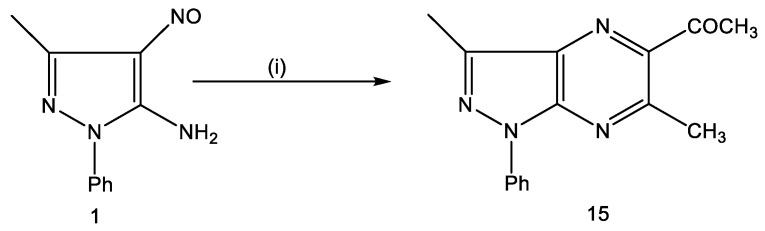
Synthesis of compound **15**. Reagents and conditions: (i) CH_2_(COCH_3_)_2_, dry pyridine, reflux 8 h.

The interaction of **1** with acetylacetone under the same reaction conditions, the expected product 5-acetyl-3,6-dimethyl-1-phenyl-1*H*-pyrazolo[3,4-*b*]pyrazine (**15**) was obtained (Scheme 4).

**Scheme 5:**
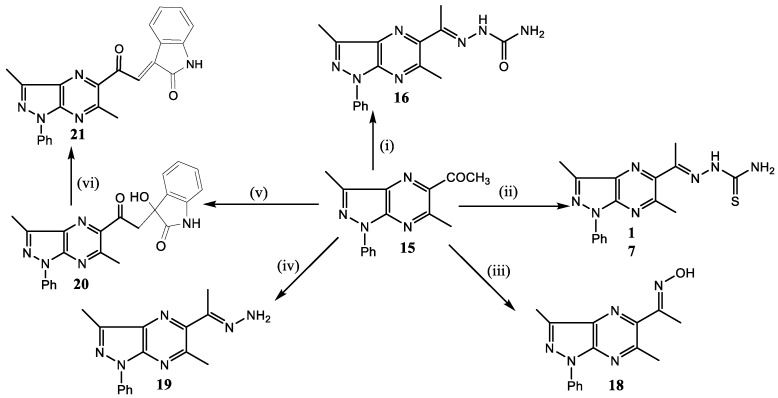
Synthesis of compound **16**–**21**. Reagents and conditions: (i) NH_2_NHCONH_2_·HCl, EtOH, reflux 8 h; (ii) NH_2_NHCSNH_2_, EtOH, reflux 8 h; (iii) NH_2_OH·HCl, sodium acetate, EtOH, reflux 3–4 h; (iv) NH_2_NH_2_, reflux 6 h; (v) isatin, (C_2_H_5_)_2_NH, EtOH, reflux overnight; and (v) EtOH, HCl, reflux 15 min.

The acetyl derivative **15** was shown to be a useful key intermediate for the synthesis of several derivatives of the title compounds. Thus, the condensation of **15** with semicarbazide and thiosemicarbazide in boiling ethanol afforded the corresponding semicarbazone and thiosemicarbazone **16** and **17**, respectively. On the other hand, when the acetyl function of **15** was interacted with hydroxyl amine the reaction product was identified as the oxime **18**. The hydrazone **19** was obtained *via* the interaction of **15** with hydrazine hydrate. Crossed aldol condensation between 5-acetylpyrazolo[3,4-*b*]pyrazine **15** and isatin was carried out in the presence of diethyl amine as a basic catalyst to give the 3**-**hydroxy-3-(2-(3,6-dimethyl-1-phenyl-1*H*-pyrazolo[3,4-*b*] pyrazine-5-yl)-2-oxoethyl)indolin-2-one (**20**). Dehydration of **20** by heating its ethanolic solution under reflux in the presence of concentrated HCl afforded the corresponding chalcone **21** (Scheme 5).

**Scheme 6:**
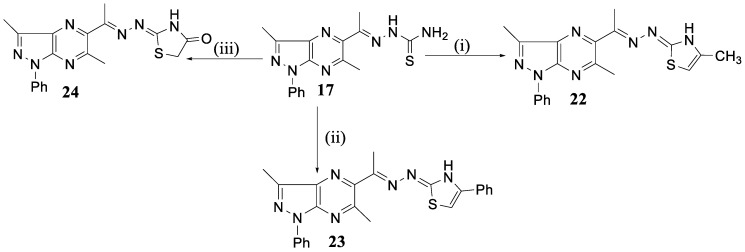
Synthesis of compound **22**–**24**. Reagents and conditions: (i) ClCH_2_COCH_3_, anhyd. CH_3_COONa, EtOH reflux 5 h; (ii) PhCOCH_2_Br, anhyd. CH_3_COONa, EtOH, reflux 5 h; and (iii) ClCH_2_COOH, anhyd. CH_3_COONa, EtOH, reflux 5 h.

When the thiosemicarbazone **17** was allowed to react with *α*-haloketones, such as chloroacetone and phenacyl bromide, the corresponding thiazolines **22**, **23** were obtained, while its reaction with *α*-chloroacetic acid gave the thiazolidinone **24** (Scheme 6).

**Scheme 7:**
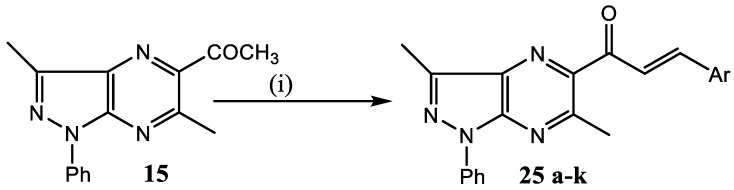
Synthesis of chalcones **25a**–**k**. Reagents and conditions: (i) ArCHO, EtOH, 25% aq. NaOH, rt overnight. **a**, Ar = C_6_H_5_; **b**, Ar = 2-OHC_6_H_4_; **c**, Ar = 2-NO_2_C_6_H_4_; **d**, Ar = 4-NO_2_C_6_H_4_; **e**, Ar = 3-NO_2_C_6_H_4_; **f**, Ar = 4-CNC_6_H_4_; **g**, Ar = 4(CH_3_O)C_6_H_4_; **h**, Ar = 4-N(CH_3_)_2_C_6_H_4_; **i**, Ar = 4-ClC_6_H_4_; **j**, Ar = 3,4-(CH_3_O)_2_C_6_H_3_; and **k**, Ar = CH=CH-C_6_H_4._

Chalcones are known by their biological activities and in particular by their anticancer activities [23,24,25,26,27,28,29]. Accordingly, a series of chalcones **25a**–**k** was synthesized *via* the Claisen-Schmidt reaction of **15** with a number of aromatic aldehydes (Scheme 7) for the sake of their evaluation against MCF-7 breast cancer cells.

**Scheme 8:**
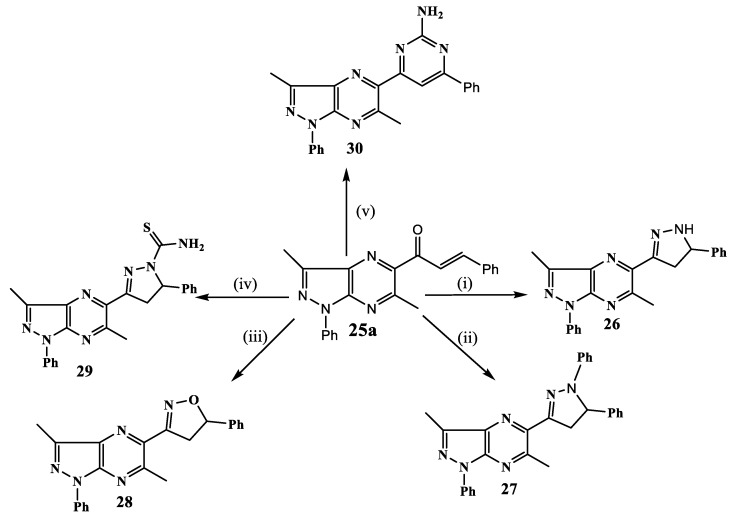
Synthesis of compounds **26**–**30**. Reagents and conditions: (i) NHNH_2_·H_2_O, EtOH, reflux 10 h; (ii) PhNHNH_2_, EtOH, reflux 15 min; (iii) NH_2_OH·HCl, CH_3_COONa, EtOH, reflux 6 h; (iv) NH_2_NHCSNH_2_, ethanolic NaOH (25%), reflux 3 h; and (v) NH_2_C=NHNH_2_·H_2_SO_4_, ethanolic KOH (25%), reflux 3–4 h.

On the other hand, the *α*,*β*-unsaturated ketonic function of chalcones renders them ready to undergo reaction with bidentate nucleophiles to give five- and six-membered heterocyclic rings. Thus, the reaction of **25a** with hydrazine hydrate and phenyl hydrazine in ethanol gave the corresponding pyrazolinyl derivatives **26** and **27**, respectively. Also, the reaction of **25a** with hydroxylamine hydrochloride in the presence of anhydrous sodium acetate led to the formation of the dihydroisoxazole **28**. Moreover, the interaction of **25a** with thiosemicarbazide in an ethanolic sodium hydroxide solution (25%) yielded the pyrazolylthioamide **29**. Finally, the treatment of **25a** with guanidine sulfate in ethanolic potassium hydroxide solution (10%) yielded the 2-aminopyrimidine **30** (Scheme 8).

### 2.2. Biology

#### 2.2.1. Anti-Inflammatory Activity

Anti-inflammatory activity of compounds (**15**, **25a**, **26**–**30**) was evaluated against the carrageenan-induced rat oedema using indomethacin as the reference drug [30]. Mean changes in paw oedema thickness of the animals pretreated with the tested compounds after 0.5, 1, 2, 3, 4, and 5 h from induction of inflammation was measured, and the inhibition percent of oedema by the tested compounds was calculated. The relative potencies % of the tested compounds compared with indomethacin at the fifth hour was also calculated (Table 1). Amongst all the tested pyrazolopyrazines, the starting compound 5-acetyl-3,6-dimethyl-1-phenyl-1*H*-pyrazolo[3,4-*b*] pyrazine (**15**) showed the highest anti-inflammatory activity, compared to that of indomethacin (44.44%). This activity was decreased when the acetyl derivative **15** was converted into its corresponding chalcone **25a**, displaying 12.5% inhibition. An increase of this latter activity was shown by compounds 26 and 27, having pyrazolinyl substituent at 5-position of the pyrazolopyrazine nucleus (23.6%, 15.07% respectively). Substituting the 5-pyrazolinyl moiety by aminopyrimidinyl ring (compound **30**) results in more increase in activity (27%). Higher activity (34%) was observed when the latter aminopyrimidinyl ring was replaced by isoxazolinyl moiety (compound **28**). Noticeable increase of potency (close to that of indomethacin) was shown by the pyrazolylthioamide derivative **29** (40%).

The percent oedema inhibition was calculated from the mean effect shown by the control and treated animals according to the following equation:

$$\mathrm{Percent}\;\mathrm{oedema}\;\mathrm{inhibition}=\frac{v_{c}-v_{t}}{v_{c}}\times 100$$

where v_c_ represents the mean increase in paw volume in the control group of rats and v_t_ represents the mean increase in paw volume in rats treated with tested compounds. The potency was calculated as the percentage of the change of the standard and tested compounds, as depicted in Table 1. All the results are expressed as the mean ± standard error of the mean (S.E.M.). Statistical evaluations were performed using graph pad prism program software version 5.00 through One-way ANOVA.

#### 2.2.2. Cytotoxic Activity

**Figure 1:**
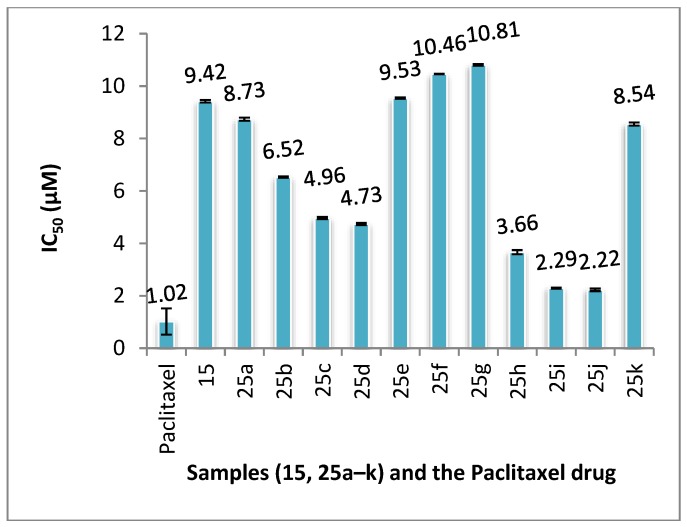
Effect of compounds (**15**, **25a**–**k**) on the proliferation of MCF-7 breast cancer cells using 3-(4,5-dimethylthiazol-2yl)-2,5-diphenyl tetrazolium bromide (MTT) cell viability assay. The IC_50_ of the tested compounds was calculated after 24 h.

**Figure 2:**
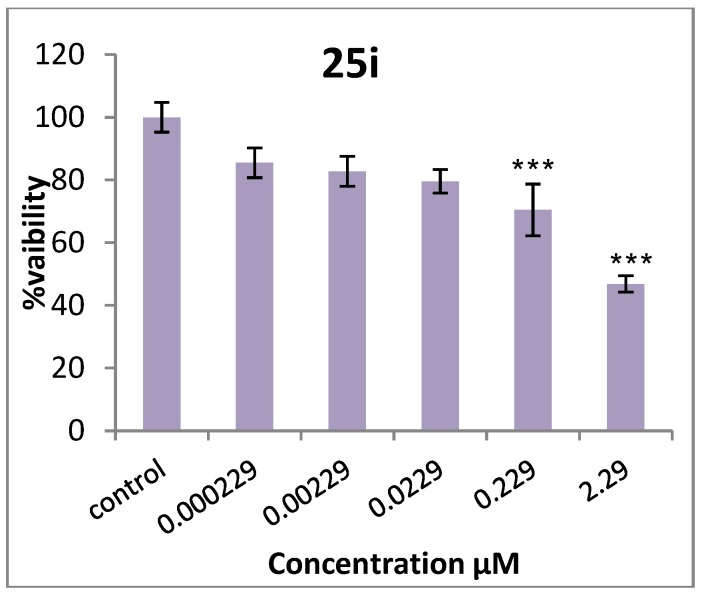
Cytotoxic effect of compounds **25i**, **25j** against the proliferation of MCF-7 breast cancer cells. Cells were treated with the indicated doses and cell viability was obtained using MTT cell viability assay. *** *p* < 0.001 calculated by comparing each concentration with the control. The data were normally distributed and were expressed as the mean ± standard error of the mean (SEM). Two-tailed paired student test *p*-values as determined by Graphpad Prism software is indicated as *** *p* < 0.001.

The chalcones **25a**–**k** along with their starting compound **15** were evaluated for their cytotoxic activity against MCF-7 breast cancer cells using 3-(4,5-dimethylthiazol-2yl)-2,5-diphenyl tetrazolium bromide (MTT) cell viability assay according to literature procedure [31]. The results obtained revealed that the parent acetyl compound **15** exhibited an inhibitory activity with IC_50_ value of 9.42 μM (Figure 1). The chalcone **25a**, with unsubstituted phenyl group in the *α*,*β*-unsaturated ketonic function, showed higher activity than that of the parent compound **15**. An increase in activity was observed upon replacement of this phenyl ring by a phenyl ethen-2-yl group (**25k**). The effect of substitution in the phenyl group of **25a** on the cytotoxic activity was studied. Thus, the introduction of an OH group in the 2-position of this phenyl group (**25b**) led to an improvement of the activity. On the other hand, the introduction of an NO_2_ group showed variable activities according to its position, where the activity was in the order 4- > 2- > 3-nitro isomer (**25d**, **25c**, **25e**). Replacement of the 4-NO_2_ group by a CN or OCH_3_ function (**25f** and **25g**) lowered the cytotoxic activity. However, its replacement by 4*-N*,*N*-dimethylamino group (**25h**) resulted in higher cytotoxic activity than all the former derivatives with IC_50_ of 3.66 μM. A further higher activity was shown by the 4-Cl derivative (**25i**), while the highest activity was shown by the 3,4-dimethoxy derivative (**25j**) with IC_50_ value of 2.22 μM. The reference drug Paclitaxel showed an IC_50_ of 1.02 μM (Figure 2).

## 3. Experimental

### 3.1. Chemistry

All melting points were determined on a Stuart melting point apparatus SMP3 (Sigma-Aldrich, Saint Louise, MI, USA). IR spectra were recorded on a Nicolet iS10 FT-IR spectrometer (Thermo Fisher, Waltham, MA, USA) using KBr wafer technique. The ^1^H-NMR spectra were recorded on Bruker AV500 (400 MHz) (Bruker, Billerica, MA, USA) and Bruker Avance III (400 MHz) spectrometers (Bruker). ^1^H- and ^13^C-NMR chemical shifts δ were reported in parts per million (ppm) and were referenced to the solvent peak; CDCl_3_ (7.26 ppm for ^1^H and 76.90 ppm for ^13^C) and DMSO-*d*_6_ (2.50 ppm for ^1^H and 39.70 ppm for ^13^C). Multiplicities are represented by s (singlet), d (doublet), t (triplet), q (quartet), and m (multiplet). Coupling constants (*J*) are reported in Hertz (Hz). Mass spectra were taken on a Jeol JMS-600 mass spectrometer (Jeol Inc., Peabody, MA, USA). Elemental analyses were carried out using a Perkin Elmer 240 C Micro analyzer (Perkin Elmer, Waltham, MA, USA) and they were found to be within ±0.4% of the theoretical values. Their results were found in good agreement with the calculated values. Paclitaxel and 3-(4,5-dimethylthiazol-2yl)-2,5-diphenyl tetrazolium bromide (MTT) were purchased from Sigma Aldrich, more detailed IR and NMR data can be found in the Supplementary Materials.

*6-Hydroxy-3-methyl-1-phenyl-1H-pyrazolo[3,4-b]pyrazine-5-carbonitrile* (**6a**). Procedure (1): A mixture of compound **1** (3.0 g, 1.5 mmol) and ethyl cyanoacetate (1.8 mL, 1.5 mmol) in dry pyridine (20 mL) was heated under reflux for overnight. After cooling, the solid product obtained was filtered, washed with water, and dried. Recrystallization from ethanol-water mixture (2:1) gave fine yellow needles, yield 0.68 g (68%), m.p. 240–242 °C. Procedure (2): Compound **7** [13] (0.5 g, 2 mmol) was dissolved in cold H_2_SO_4_ (5 mL), then a cold sodium nitrite solution (0.4 g in 2 mL H_2_O) was added dropwise under stirring to the above solution during 0.5 h in an ice bath at 0–5 °C. The reaction mixture was allowed to stir at room temperature (rt) for 1 h further, then it was poured onto ice-water mixture. The solid product obtained was filtered, washed with water, and recrystallized from ethanol-water mixture (2:1) as yellow crystals, yield 0.3 g (60%), m.p. 240–242 °C. IR (ν, cm^−1^): 3197 (OH), 3073 (CH aromatic), 2234 (C≡N). ^1^H-NMR (400 MHz, DMSO-*d*_6_) δ (ppm): 2.51 (s, 3H, CH_3_), 7.38 (t, 1H, phenyl), 7.57 (t, 2H, phenyl), 8.08 (d, 2H, phenyl), 8.43 (s, 1H, OH). ^13^C-NMR (100 MHz, DMSO-*d*_6_) δ (ppm): 10.99 (CH_3_), 114.58 (C), 115.73 (C≡N), 120.28 (2 CH), 126.55 (CH), 129.01 (C), 129.31 (2CH), 137.89 (C), 141.84 (C), 144.48 (C), 160.40 (C) MS: *m*/*z* (251 M^+^). Anal. calcd. for C_13_H_9_N_5_O (251.24): C, 62.15; H, 3.61; N, 27.87. Found: C, 62.30; H, 3.50; N, 27.72%.

*6-Hydroxy-3-methyl-1-phenyl-1H-pyrazolo[3,4-b]pyrazine-5-carboxylic acid* (**8**)*:* The hydroxy cyano compound **6a** (0.5 g) was dissolved in EtOH (10 mL), then aqueous solution of NaOH (20%, 10 mL) was added. The reaction mixture was heated under reflux for 5 h, then it was evaporated to half of its volume. After cooling, it was neutralized with dil. HCl, giving a solid precipitate which was filtered off, washed with water, dried, and recrystallized from dioxane as yellow crystals, yield 0.4 g (80%), m.p. 190–192 °C. IR (ν, cm^−1^): 3493 (OH), 2969, 2920, 2862 (CH aliph.), 3350-2493 (characteristic of COOH), 1667 (C=O). ^1^H-NMR (400 MHz, DMSO-*d*_6_) δ (ppm): 2.57 (s, 3H, CH_3_), 7.33 (t, 1H, phenyl), 7.55 (t, 2H, phenyl), 8.12 (d, 2H, phenyl), 8.38 (s, 1H, OH). ^13^C-NMR (100 MHz, DMSO-*d*_6_): 11.76 (CH_3_), 119.67 (2CH, C), 125.59 (CH, C), 129.53 (2CH, C), 139.55 (2C), 144.77 (C), 165.16 (C). Anal. calcd. for C_13_H_10_N_4_O_3_ (270.24): C, 57.78; H, 3.73; N, 20.73. Found**.** C, 57.66; H, 3.88; N, 20.56%.

*Ethyl 6-hydroxy-3-methyl-1-phenyl-1H-pyrazolo[3,4-b]pyrazine-5-carboxylate* (**9**): To the hydroxy acid **8** (0.5 g, 1 mmol) in anhydrous ethanol (20 mL), conc. HCl (15 mL) was added, and the mixture was heated under reflux for 6 h. The reaction mixture was then cooled, poured on ice-cold solution of NaHCO_3_, and the solid precipitate was filtered, washed with water, and dried. Crystallization from dioxane-water mixture (2:1) gave pale brown crystals, yield 0.35 g (70%), m.p. 143–145 °C. IR (ν, cm^−1^): 3117 (OH), 2974 (CH aliph.), 1677 (C=O). ^1^H-NMR (400 MHz, CDCl_3_) δ (ppm): 1.57 (t, *J* = 7.1 Hz, 3H, CH_3_), 2.76 (s, 3H, CH_3_), 4.64 (q, *J* = 7.1 Hz, 2H, CH_2_), 7.33 (t, 1H, phenyl), 7.53 (t, 2H, phenyl), 8.25 (d, 2H, phenyl), 12.08 (s, 1H, OH),^13^C-NMR (100 MHz, CDCl_3_): 11.64 (CH_3_), 14.20 (CH_3_), 63.48 (CH_2_), 120.32 (2 CH), 123.05 (C) 126.37 (CH), 129.23 (2 CH), 130.58 (C), 138.39 (C), 143.09 (C), 145.8 (C), 161.27 (C), 169.07 (C=O). MS: *m*/*z* (298 M^+^). Anal. calcd. for C_15_H_14_N_4_O_3_ (298.30): C, 60.40; H, 4.73; N, 18.78. Found: C, 60.30; H, 4.50; N, 18.92%.

*5-Acetyl-3-methyl-1-phenyl-1H,6H-imidazo[4,5-c]pyrazole* (**10**): A mixture of compound **1** (1.0 g, 5 mmol) and chloro acetone (0.46 mL, 5 mmol) in dry pyridine (15 mL) was heated under reflux for overnight. After cooling the solid product was filtered, washed with water, and recrystallized from ethanol-dioxane mixture (2:1) as fine yellow needles, yield 0.52 g (98%), m.p. 288–290 °C. IR (ν, cm^−1^): 3438 (NH), 2922, 2784 (CH aliph.), 1655 (C=O). ^1^H-NMR (400 MHz, DMSO-*d*_6_) δ (ppm): 2.40 (s, 3H, CH_3_), 2.60 (s, 3H, CH_3_), 7.33 (t, 1H, phenyl), 7.52 (t, 2H, phenyl), 7.6 (s, 1H, NH), 8.11 (d, 2H, phenyl). ^13^C-NMR (100 MHz, DMSO-*d*_6_) δ (ppm): 11.26 (CH_3_), 21.17 (CH_3_), 119.87 (2CH), 125.79 (CH, C), 129.19 (2CH, C), 138.82 (2C), 155.33 (C=O). Anal. calcd. for C_13_H_12_N_4_O (240. 26): C, 64.99; H, 5.03; N, 23.32. Found: C, 64.60; H, 5.21; N, 23.40%.

*5-Benzoyl-3-methyl-1-phenyl-1H,6H-imidazo[4,5-c]pyrazole* (**11**): A mixture of compound **1** (1.0 g, 5 mmol) and phenacyl bromide (0.46 g, 5 mmol) in dry pyridine (15 mL) was heated under reflux for overnight. After cooling the solid product was filtered, washed with water, dried, and recrystallized from ethanol-dioxane mixture (1:1) as yellow needles, yield 0.8 g (88%), m.p. 304–306 °C. IR (ν, cm^−1^): 3075 (NH), 2921, 2781 (CH aliph.), 1641 (C=O). ^1^H-NMR (400 MHz, DMSO-*d*_6_) δ (ppm): 2.76 (s, 3H, CH_3_), 7.33 (t, 1H, phenyl), 7.41 (t, 1H, phenyl), 7.65–7.50 (m, 5H, phenyl), 8.12–8.31 (m, 3H, phenyl), 12.85 (s, 1H, NH). ^13^C-NMR (100 MHz, DMSO-*d*_6_) δ (ppm): 14.08 (CH_3_), 120.327 (C), 120.99 (2CH), 123.08 (CH), 126.78 (CH), 127.02 (CH), 127.96 (CH), 128.45 (CH), 129.57 (CH), 129.77 (CH), 129.88 (CH), 131.49 (C), 135.14 (C), 139.11 (C), 154.56 (C), 155.46 (C), 177.73 (C). MS: *m*/*z* (302 M^+^). Anal. calcd. for C_18_H_14_N_4_O (302.33): C, 71.51; H, 4.67; N, 18.53. Found: C, 71.62; H, 4.25; N, 18.72%.

*3-Methyl-1-phenyl-7-thioxo-7,8-dihydro-1H-pyrazolo[4,3-g]pteridin-5(6H)-one* (**12**): A mixture of amino nitroso **1** (1.01 g, 5 mmol) and thiobarbituric acid (0.72 g, 5 mmol) in dry pyridine (20 mL) was heated under reflux for overnight. After cooling, the solid product was filtered, washed with water, and recrystallized from ethanol-dioxane mixture (1:3) as fine yellow needles, yield 0.15 g (65%), m.p. 332–334 °C. IR (ν, cm^−1^): 3416 (NH), 3068 (CH arom.), 2891 (CH aliph.), 1693 (C=O). ^1^H-NMR (400 MHz, DMSO-*d*_6_), δ (ppm): 2.70 (s, 1H, CH_3_), 7.27 (t, 1H, phenyl), 7.45 (t, 2H, phenyl), 8.13 (d, 2H, phenyl), 11.76 (s, 1H, NH), 11.91 (s, 1H, NH), ^13^C-NMR (100 MHz, DMSO-*d*_6_) δ (ppm): 11.29 (s, 3H, CH_3_), 119.60 (2CH), 126.36 (CH), 126.99 (C), 129.37 (2CH), 132.78 (C), 138.13 (C), 142.42 (C), 145.60 (C), 147.27 (C), 158.31 (C=O), 175.95 (C=S). MS: *m*/*z* (310 M^+^). Anal. calcd. for C_14_H_10_N_5_OS (310.3): C, 54.18; H, 3.25; N, 27.08 Found: C, 54.02; H, 3.21; N, 26.82%.

*6-Ethyl-7-(ethylthio)-3-methyl-1-phenyl-1H-pyrazolo[4,3-g]pteridin-5(6H)-one* (**13**): A mixture of the thione **12** (0.25 g, 0.8 mmol), ethyl iodide (0.24 g, 1.6 mmol), and anhydrous K_2_CO_3_ (0.41 g, 3 mmol) in DMF(15 mL) was stirred at 80 °C for 5 h. After cooling the solid product was filtered, washed with water, dried, and recrystallized from ethanol-acetone mixture (2:1) to give yellow crystals, yield 0.25 g (86%), m.p. 238–240 °C. IR (ν, cm^−1^): 2922, 2784 (CH aliph.), 1655 (C=O). ^1^H-NMR (400 MHz, CDCl_3_) δ (ppm): 1.47 (t, *J* = 7.1 Hz, 3H, CH_3_), 1.53 (t, *J* = 7.4 Hz, 3H, CH_3_), 2.86 (s, 3H, CH_3_), 3.51 (q, *J* = 7.4 Hz, 2H, CH_2_), 4.34 (q, *J* = 7.0 Hz, 2H, CH_2_), 7.35 (t, *J* = 7.3 Hz, 1H, phenyl), 7.57 (t, *J* = 7.8 Hz, 2H, phenyl), 8.33 (d, *J* = 8.4 Hz, 2H, phenyl). ^13^C-NMR (100 MHz, CDCl_3_) δ (ppm): 11.75 (CH_3_), 12.93 (CH_3_), 13.68 (CH_3_), 27.05 (CH_2_), 40.44 (CH_2_), 120.25 (2CH), 125.89 (CH), 128.22 (C), 129.21 (2CH), 135.53 (C), 138.67 (C), 144.52 (C), 145.96 (C), 151.08 (C), 160.43 (C), 162.82 (C=O). MS: *m*/*z* (366 M^+^). Anal. calcd. for C_18_H_18_N_6_OS (366.44): C, 59.00; H, 4.95; N, 22.93. Found: C, 59.25; H, 4.72; N, 22.65%.

*Ethyl 2-(3-methyl-5-oxo-1-phenyl-5,6-dihydro-1H-pyrazolo[4,3-g]pteridin-7-ylthio)acetate.* (**14**): A mixture of the thione **12** (0.15 g, 0.4 mmol), ethyl chloroacetate (0.1 g, 0.8 mmol), and anhydrous K_2_CO_3_ (0.44 g, 3 mmol) in DMF (15 mL) was stirred at 80 °C for 5 h. After cooling the solid product was filtered, washed with water, dried, and recrystallized from dioxane-water mixture (2:1) to give yellow crystals, yield 0.10 g (71.4%), m.p. 243–245 °C. IR (ν, cm^−1^): 2980, 2923 (CH aliph.), 1737 (C=O), 1686 (C=O). ^1^H-NMR (400 MHz, CDCl_3_) δ (ppm): 1.28 (t, *J* = 17.3, 10.2 Hz, 3H, CH_3_), 2.78 (s, 3H, CH_3_), 4.34–4.11 (m, 4H, 2CH_2_), 7.27 (t, 1H, phenyl), 7.47 (t, 2H, phenyl), 8.23 (d, 2H, phenyl), 9.64 (s, 1H, NH). ^13^C-NMR (100 MHz, CDCl_3_) δ (ppm): 11.79 (CH_3_), 14.57 (CH_3_), 33.31 (CH_2_), 61.73 (CH_2_), 120.21 (2CH), 126.65 (CH), 129.78 (2CH), 130.09 (C), 134.73 (C), 138.76 (C), 144.31 (C), 145.79 (C), 160.60 (C), 161.41 (C), 162.100 (C=O), 168.64 (C=O). Anal. calcd. for C_18_H_16_N_6_O_3_S (396.42): C, 54.54; H, 4.07; N, 21.20. Found: C, 54.66; H, 4.12; N, 21.13%.

*5-Acetyl-3,6-dimethyl-1-phenyl-1H-pyrazolo[3,4-b]pyrazine* (**15**): A mixture of amino nitroso compound **1** (6.06 g, 30 mmol) and acetylacetone (3.0 g, 30 mmol) in dry pyridine (20 mL) was heated under reflux for 8 h. The reaction mixture was then evaporated to one-half volume and after cooling it was neutralized with dil. HCl (10%). The solid precipitate was filtered off, washed with water, and recrystallized from ethanol-water mixture (1:3) as pale brown needles, yield 6.4 g (80%), m.p. 130–132 °C. IR (ν, cm^−1^): 3050 (CH arom.), 2900 (CH aliph.), 1689 (C=O). ^1^H-NMR (400 MHz, CDCl_3_) δ (ppm): 2.70 (s, 3H, CH_3_), 2.80 (s, 3H, CH_3_), 3.00 (s, 3H, CH_3_), 7.27–7.23 (t, 1H, phenyl), 7.51–7.56 (t, 2H, phenyl), 8.23–8.31 (d, 2H, phenyl). ^13^C-NMR (100 MHz, CDCl_3_) δ (ppm): 11.32 (CH_3_), 25.15 (CH_3_), 27.79 (CH_3_), 120.15 (2CH), 126.16 (CH), 129.18 (2CH), 131.83 (C), 138.85 (C), 142.66 (C), 143.18 (C), 144.82 (C), 154.14 (C), 200.54 (C=O). MS: *m*/*z* (266 M^+^). Anal. calcd. for C_15_H_14_N_4_O (266. 12): C, 67.65; H, 5.30; N, 21.04. Found: C, 67.63; H, 5.32; N, 21.08%.

*(3,6-Dimethyl-1-phenyl-5-acetyl-1H-pyrazolo[3,4-b]pyrazine)semicarbazone* (**16**): A mixture of the acetyl derivative **5** (0.72 g, 3 mmol), semicarbazide hydrochloride (0.3 g, 3 mmol), and sodium acetate (0.6 g, 1 mol) in ethanol (15 mL) was heated under reflux for 8 h. After cooling, the product was filtered, washed with water, and recrystallized from dioxane-water mixture (3:1) as yellow crystals, yield 0.53 g (93%), m.p. 210–212 °C. IR (ν, cm^−1^): 3487, 3204 (NH_2_ + NH), 1739 (C=O). MS: *m*/*z* 323 (M^+^). Anal. calcd. for C_16_H_17_N_7_O (323.35): C, 59.43; H, 5.30; N, 30.32. Found: C, 59.33; H, 5.41; N, 30.26%. No numerical data could be obtained for this compound due its insolubility in deuterated solvents.

*(3,6-Dimethyl-1-phenyl-5-acetyl-1H-pyrazolo[3,4-b]pyrazine)thiosemicarbazone* (**17**): A mixture of **15** (1.0 g, 4 mmol) and thiosemicarbazide (0.34 g, 4 mmol) in ethanol (10 mL) was heated under reflux for 8 h. After cooling, the solid product was filtered, washed with water, and dried. Crystallization from dioxane-water mixture (1:1) gave yellow crystals, yield 0.28 g (87%); m.p. 230–232 °C; IR (ν, cm^−1^): 3434, 3237, 3152 (NH_2_ + NH), 1690 (C=O).^1^H-NMR (300 MHz, CDCl_3_) δ (ppm): 2.40 (CH_3_), 2.82 (CH_3_), 2.93 (CH_3_), 6.40 (s, 2H, NH_2_), 7.32–7.35 (t, 1H, phenyl), 7.51–7.56 (t, 2H, phenyl), 8.27–8.31 (d, 2H, phenyl), 8.88 (s, 1H, NH). ^13^C-NMR (100 MHz, CDCl_3_) δ (ppm): 11.49 (CH_3_), 25.00 (CH_3_), 27.96 (CH_3_), 120.29 (C, 2CH), 126.7 (CH), 129.7 (2CH), 131 (C), 138 (C), 142.6 (C), 143.8 (C), 144.77 (C), 153.8 (C), 200.19 (C=S). Anal. calcd. for C_16_H_17_N_7_S (339.42): C, 56.62; H, 5.05; N, 28.89. Found: C, 56.33; H, 5.20; N, 28.66%.

*(5-Acetyl-3,6-dimethyl-1-phenyl-1H-pyrazolo[3,4-b]pyrazine)oxime* (**18**): A mixture of **15** (0.25 g, 0.9 mmol), hydroxyl amine (0.07 g, 1 mmol), and sodium acetate (0.25 g, 3 mmol) in ethanol (10 mL ethanol) was heated under reflux for 3 h. The reaction mixture was then evaporated to half volume and after cooling it was neutralized with dil. HCl. The solid precipitate was filtered off, washed with water, and recrystallized from ethanol-water mixture (3-1) as fine buff needles, yield 0.16 g (89%), m.p. 223–225 °C, IR (ν, cm^−1^): 3281 broad (OH), 2900 (CH aliph.). ^1^H-NMR (400 MHz, DMSO-*d*_6_) δ (ppm): 2.29 (s, 3H, CH_3_), 2.63 (s, 3H, CH_3_) 2.81 (s, 3H, CH_3_), 7.31–7.36 (t, 1H, phenyl), 7.54–7.59 (t, 2H, phenyl), 8.20–8.23 (d, 2H, phenyl), 11.59 (s, 1H, OH). ^13^C-NMR (100 MHz, DMSO) δ (ppm): 11.01 (s, 3H, CH_3_), 13.08 (s, 3H, CH_3_), 25.11 (s, 3H, CH_3_), 119.52 (2CH), 125.81 (CH), 129.29 (2CH), 131.11 (C), 138.69 (C), 141.37 (C), 143.08 (C), 146.15 (C), 151.87 (C), 154.14 (C). MS: *m*/*z* (281 M^+^). Anal. calcd. for C_15_H_15_N_5_O (281.31): C, 64.04; H, 5.37; N, 24.90. Found: C, 64.11; H, 5.42; N, 24.97%.

*(5-Acetyl-3,6-dimethyl-1-phenyl-1H-pyrazolo[3,4-b]pyrazine)hydrazone* (**19**): A mixture of 15 (0.25 g, 0.9 mmol) and hydrazine hydrate (0.5 mL, 80%) in ethanol (10 mL) was heated under reflux for 6 h. After cooling the solid product was filtered, washed with water, and recrystallized from ethanol as buff needles, yield 0.2 g (87%), m.p. 136–138 °C. IR (ν, cm^−1^): 3453, 3342 (NH_2_), 3050 (CH arom.). ^1^H-NMR (400 MHz, CDCl_3_) δ (ppm): 2.3 (s, 3H, CH_3_), 2.70 (s, 3H, CH_3_) 2.90 (s, 3H, CH_3_), 5.58 (s, 2H, NH_2_), 7.27–7.31 (t, 1H, phenyl), 7.49–7.54 (t, 2H, phenyl), 8.20–8.31 (d, 2H, phenyl). ^13^C-NMR (100 MHz, CDCl_3_) δ (ppm): 11.36 (CH_3_), 12.56 (CH_3_), 25.65 (CH_3_), 120.03 (CH), 120.14 (CH), 125.60 (CH), 129.12 (CH), 129.18 (CH), 131.52 (C), 139.37 (C), 141.94 (C), 143.48 (C), 147.35 (C), 147.78 (C), 152.34 (C). MS: *m*/*z* (280 M^+^). Anal. calcd. for C_15_H_16_N_6_ (280.33): C, 64.27; H, 5.75; N, 29.98. Found: C, 64.11; H, 5.42; N, 29.93%.

*5-(3-Hydroxy-2,3-dihydroindol-2-on-3-ylacetyl)-3,6-dimethyl-1-phenyl-1H-pyrazolo[3,4-b]pyrazine* (**20**): A mixture of **15** (0.25 g, 0.9 mmol) and isatin (0.14 g, 0.9 mmol) and diethyl amine (0.5 mL) in absolute ethanol (15 mL) was stirred at rt for overnight. The solid product obtained was filtered and recrystallized from ethanol-water mixture (1:1) as buff crystals, yield 0.18 g (90%), m.p. 167–169 °C. IR (ν, cm^1^), 3322 (NH), 1745 (C=O), 1697 (C=O). **^1^**H-NMR (400 MHz, CDCl_3_) δ (ppm): 2.77 (d, *J* = 9.7 Hz, 1H, CH_2_), 2.84 (s, 6H, 2CH_3_), 3.01 (d, *J* = 11.4 Hz, 1H, CH_2_), 6.90 (s, 1H, OH), 7.07 (s, 1H, NH), 7.38–7.29 (m, 2H, Ar-H), 7.44 (d, 2H, Ar-H), 7.55 (t, 2H, Ar-H), 8.34–8.24 (m, 3H, Ar-H). ^13^C-NMR (100 MHz, CDCl_3_) δ (ppm): 11.25 (CH_3_), 25.02 (CH_3_), 27.46 (CH_2_), 45.61 (C), 110.11 (C), 115.52 (C), 120.31 (CH), 120.40 (CH), 123.03 (CH), 124.52 (CH), 126.19 (CH), 126.41 (CH), 129.20 (2CH), 129.98 (CH), 131.45 (C), 138.65 (C), 142.78 (C), 144.86 (C), 148.28 (C), 151.96 (C), 169.03 (C), 191.12 (C=O). Anal. calcd. for C_23_H_21_N_5_O_3_ (413.4): C, 66.82; H, 4.63; N, 16.94. Found: C, 66.51; H, 5.43; N, 16.93%.

*2,3-Dihydroindol-2-on-3-ylideneacetyl-3,6-dimethyl-1-phenyl-1H-pyrazolo[3,4-b]pyrazine* (**21**): Compound **20** (0.18 g, 4 mmol) was heated in a mixture of ethanol (7.5 mL) and conc. HCl (2.5 mL) for 15 min. After cooling the solid product obtained was filtered, washed with water, and recrystallized from ethanol-dioxane mixture (1:3) as orange needles, yield 0.13 g (81%), m.p. 248–250 °C. IR (ν, cm^−1^), 3444 (NH), 3163, 3074 (CH arom.), 1716 (C=O), 1670 (CO-NH). ^1^H-NMR (300 MHz, CDCl_3_) δ (ppm): 2.90 (s, 3H, CH_3_), 3.20 (s, 3H, CH_3_), 6.85–6.89 (d, 1H, phenyl), 7.10 (t, 1H, phenyl), 7.34–7.37 (t, 2H, phenyl), 7.52–7.55 (t, 2H, phenyl), 7.50 (s, 1H, NH), 8.30–8.33 (d, 2H, phenyl), 8.56 (s, 1H, CH), 8.68–8.71 (d, 1H, phenyl). ^13^C-NMR (100 MHz, DMSO-*d*_6_) δ (ppm): 11.72 (CH_3_), 25.32 (CH_3_), 110.80 (CH), 120.43 (CH), 120.58 (2CH), 122.29 (CH), 126.73 (CH), 126.87 (CH), 128.37 (CH), 129.87 (2CH), 131.56 (C), 133.90 (C), 137.79 (C), 138.71 (C), 142.55 (C), 144.31 (C), 145.09 (C), 145.88 (C), 155.16 (C), 168.97 (C=O), 190.75 (C=O) MS: *m*/*z* (395 M^+^). Anal. calcd. for C_23_H_19_N_5_O_2_ (395.4): C, 69.86; H, 4.33; N, 17.71. Found: C, 69.43; H, 4.64; N, 17.83%.

*N-(4-Methyl-2,3-dihydrothiazol-2-ylidene)-(5-acetyl-3,6-dimethyl-1-phenyl-1H-pyrazolo[3,4-b]pyrazine) hydrazine* (**22**): A mixture of the thiosemicarbazone **17** (0.4 g, 1 mmol), chloro acetone (0.1 mL, 1 mmol), and anhydrous sodium acetate (0.5 g) in absolute ethanol was heated under reflux for 5 h. After cooling, the solid product obtained was filtered the reaction mixture was cooled, washed with water, and recrystallized from acetone-water mixture (1:1) as buff crystals, yield 0.05 g (17%), m.p. 222–224 °C, IR (ν, cm^−1^): 3178 (NH), 3067 (CH arom.), 2921, 2852 (CH aliph.). ^1^H-NMR (400 MHz, CDCl_3_) δ (ppm): 2.36 (s, 3H, CH_3_), 2.59 (s, 3H, CH_3_), 2.74 (s, 3H, CH_3_), 3.06 (s, 3H, CH_3_), 5.66 (s, 1H, NH), 6.29 (s, 1H, CH), 7.35–7.27 (m, 1H, phenyl), 7.54 (t, 2H, phenyl), 8.33 (d, 2H, phenyl). ^13^C-NMR (100 MHz, CDCl_3_) δ (ppm): 11.46 (CH_3_), 14.02 (CH_3_), 16.71 (CH_3_), 26.43 (CH_3_), 103.13 (CH), 120.08 (2CH), 125.76 (CH), 128.74 (2CH), 131.52 (C), 141.26 (C), 139.02 (C), 143.79 (C), 145.19 (C), 146.05 (C), 149.26 (C), 152.70 (C), 169.00 (C). Anal. calcd. for C_19_H_19_N_7_S (377.47): C, 60.46; H, 5.07; N, 25.98. Found: C, 60.33; H, 5.11; N, 25.21%.

*N-(4-Phenyl-2,3-dihydrothiazol-2-ylidene)-(5-acetyl-3,6-dimethyl-1-phenyl-1H-pyrazolo[3,4-b]pyrazine) hydrazine* (**23**): A mixture of thiosemicarbazide **17** (0.25 g, 0.7 mmol), phenacyl bromide (0.3 g, 1 mmol), and anhydrous sodium acetate (0.25 g) in absolute ethanol (15 mL) was heated under reflux for 5 h. After cooling, the solid product was filtered, washed with water, and recrystallized from ethanol-dioxane mixture (3:1) as brown crystals, yield 0.11 g (40%), m.p. 256–258 °C. IR (ν, cm^−1^): 3435 (NH), 3065 (CH arom.), 2924 (CH aliph.). ^1^H-NMR (400 MHz, CDCl_3_) δ (ppm): 2.53 (s, 3H, CH_3_), 2.73 (s, 3H, CH_3_), 3.09 (s, 3H, CH_3_), 6.90 (s, 1H, CH), 7.30–7.35 (t, 2H, phenyl), 7.51–7.56 (t, 2H, phenyl), 7.39–7.44 (t, 2H, phenyl), 7.82–7.84 (d, 2H, phenyl), 8.31–8.33 (d, 2H, phenyl) 8.90 (bs, 1H, NH). MS: *m*/*z* (439 M^+^). ^13^C-NMR (100 MHz, CDCl_3_) δ (ppm): 11.49 (CH_3_), 24.85 (CH_3_), 29.07 (CH_3_), 114.30 (CH), 120.23 (2CH), 121.02 (CH), 126.13 (CH), 127.81 (CH), 129.19 (2CH), 130.52 (2CH), 131.67 (C), 138.92 (C), 142.67 (C), 144.47 (2C), 144.59 (C), 144.71 (C), 154.59 (2C), 161.76 (C). Anal. calcd. for C_24_ H_21_ N_7_ S (439.54): C, 65.58; H, 4.82; N, 22.31%. Found: C, 65.51; H, 4.75; N, 22.21%.

*N-(Thiazolidin-4-on-2-ylidene)-(5-Acetyl-3,6-dimethyl-1-phenyl-1H-pyrazolo[3,4-b]pyrazine) hydrazine* (**24**): A mixture of **17** (0.5 g, 1 mmol), chloro acetic acid (0.1 g, 0.8 mol), and anhydrous sodium acetate (0.5 g) in absolute ethanol was heated under reflux for 5 h. After cooling, the solid product obtained was filtered, washed with water, and dried. Recrystallization from ethanol-dioxane mixture (1:3) gave buff crystals, yield 0.12 g (50%), m.p. 308–310 °C. IR (ν, cm^−1^): 3446 (NH), 2850 (CH aliph.), 1705 (C=O). MS: *m/z* (379 M^+^). Anal. calcd. for C_18_H_17_N_7_OS (379.44): C, 56.98; H, 4.52; N, 25.84. Found: C, 56.88; H, 4.65; N, 25.91%.

*General procedure for the synthesis of 5-arylideneacetyl-3,6-dimethyl-1-phenyl-1H-pyrazolo[3,4-b] pyrazine* (**25a**–**k**): To a mixture of equimolar amounts (0.27, 1 mmol) of 5-acetyl-3,6-dimethyl-1-phenyl-1*H*- pyrazolo[3,4-*b*]pyrazine (**15**) and an aromatic aldehyde in ethanol (15 mL), aqueous NaOH (0.5 mL, 25%) was added. The reaction mixture was stirred at room temperature for overnight, then it was poured onto ice-cold water. The solid precipitate formed was collected by filtration, washed with water, and recrystallized from dioxane-water mixture.

*5-Benzylideneacetyl-3,6-dimethyl-1-phenyl-1H-pyrazolo[3,4-b]pyrazine* (**25a**): Yield 1.68 g (84%), m.p. 167–169 °C. IR (ν, cm^−1^): 3059 (CH arom.), 2900 (CH aliph.), 1670 (C=O). ^1^H-NMR (400 MHz, CDCl_3_) δ (ppm): 2.80 (s, 3H, CH_3_), 3.06 (s, 3H, CH_3_), 7.20–8.30 (m, 10H, phenyl),^13^C-NMR (100 MHz, CDCl_3_) δ (ppm): 11.49 (CH_3_), 24.91 (CH_3_), 120.34 (2CH), 123.29 (CH), 126.23 (CH), 128.74 (2CH), 128.95 (2CH), 129.23 (2CH), 130.60 (CH, C), 135.09 (2C), 142.69 (C), 144.55 (CH, C), 145.82 (2C), 190.23 (C=O). MS: *m*/*z* (354 M^+^). Anal. calcd. for C_22_H_18_N_4_O (354): C, 74.56; H, 5.12; N, 15.81. Found: C, 74.63; H, 5.14; N, 15.60%.

*3,6-Dimethyl-5-(2-hydroxylbenzylideneacetyl)-1-phenyl-1H-pyrazolo[3,4-b]pyrazine* (**25b**): Yield 0.14 g (70%), m.p. 194–196 °C. IR (ν, cm^−1^): 3213 (OH), 2923.40 (CH aliph.), 1657 (C=O). ^1^H-NMR (400 MHz, DMSO-*d*_6_) δ (ppm): 2.69 (s, 3H, CH_3_), 2.89 (s, 3H, CH_3_), 6.90 (t, 1H, Ar-H), 6.96 (d, *J* = 8.1 Hz, 1H, CH), 7.30 (t, 1H, Ar-H), 7.37 (t, 1H, Ar-H), 7.59 (t, 2H, Ar-H), 7.70 (d, *J* = 7.6 Hz, 1H, CH), 8.03 (s, 2H, Ar-H), 8.23 (d, 2H, phenyl), 10.32 (s, 1H, OH).^13^C-NMR (100 MHz, DMSO-*d*_6_): 11.59 (CH_3_), 24.68 (CH_3_), 116.80 (C), 120.00 (CH), 120.31(2CH), 121.73 (C), 123.33 (CH), 126.67 (CH), 129.61 (CH), 129.82 (2CH), 131.49 (CH), 132.70 (C), 138.87 (C), 140.71 (C), 142.60 (C), 144.73 (CH), 145.18 (C), 154.09 (C), 157.95 (CH), 190.71 (C=O). Anal. calcd. for C_22_H_18_N_4_O_2_ (370.40): C, 71.34; H, 4.90; N, 15.13. Found: C, 71.23; H, 4.85; N, 15.25%.

*3,6-Dimethyl-5-(2-nitrobenzylideneacetyl)-1-phenyl-1H-pyrazolo[3,4-b]pyrazine* (**25c**): Yield 0.17 g (85%), m.p. 263–265 °C. IR (ν, cm^−1^): 3066 (CH arom.), 1672 (C=O). ^1^H-NMR (400 MHz, CDCl_3_) δ (ppm): 2.84 (s, 3H, CH_3_), 3.10 (s, 3H, CH_3_), 7.37 (d, *J* = 6.5 Hz, 2H, CH, Ar-H), 7.57 (t, 2H, Ar-H), 7.90 (t, 3H, Ar-H), 8.41–8.30 (m, 4H, CH, Ar-H). ^13^C-NMR (100 MHz, CDCl_3_) δ (ppm): 11.52 (CH_3_), 25.11 (CH_3_), 120.38 (2CH), 124.20 (2CH), 126.40 (CH), 126.85 (CH), 129.18 (2CH), 129.26 (2CH, C), 138.76 (C), 140.86 (2C), 141.31 (C), 144.93 (CH, C), 155.35 (2C), 189.20 (C=O). Anal. calcd. For C_22_H_17_N_5_O_3_ (399.40): C, 66.16; H, 4.29; N, 17.53. Found: C, 66.22; H, 4.18; N, 17.41%.

*3,6-Dimethyl-5-(4-nitrobenzylideneacetyl)-1-phenyl-1H-pyrazolo[3,4-b]pyrazine* (**25d**): Yield 0.12 g (80%), m.p. 263–265 °C. IR (ν, cm^−1^): 3051 (CH arom.), 1672 (C=O). ^1^H-NMR (400 MHz, CDCl_3_) δ (ppm): 2.84 (s, 3H, CH_3_), 3.10 (s, 3H, CH_3_), 7.35–7.37 (t, *J*= 7.4 Hz, 1H, CH), 7.55–7.59 (t, 2H, Ar-H), 7.87–7.93 (t, *J* = 11.9 Hz, 2H, CH, Ar-H), 8.32–8.40 (m, 5H, Ar-H). ^13^C-NMR (100 MHz, CDCl_3_) δ (ppm): 11.52 (CH_3_), 25.11 (CH_3_), 120.37 (2CH), 124.21 (2CH), 126.40 (CH), 126.86 (CH), 129.18 (2CH), 129.26 (2CH), 131.89 (C), 138.75 (C), 140.86 (CH), 141.31 (C), 142.67 (C), 143.25 (C), 144.94 (C), 148.57 (C), 155.35 (C), 189.20 (C=O). Anal. calcd. for C_22_H_17_N_5_O_3_ (399.40): C, 66.16; H, 4.29; N, 17.53. Found: C, 66.11; H, 4.17; N, 17.42%*.*

*3,6-Dimethyl-5-(3-nitrobenzylideneacetyl)-1-phenyl-1H-pyrazolo[3,4-b]pyrazine* (**25e**): Yield 0.15 g (75%); m.p. 223–225 °C; IR (ν, cm^−1^): 3086 (CH arom.), 2925 (CH aliph.), 1674 (C=O); ^1^H-NMR (400 MHz, CDCl_3_) δ (ppm): 2.85 (s, 3H, CH_3_), 3.10 (s, 3H, CH_3_), 7.36 (t, 1H, Ar-H), 7.57 (t, 2H, Ar-H), 7.66 (t, 1H, Ar-H), 7.91 (d, *J* = 16.0 Hz, 1H, CH), 8.02 (d, *J* = 7.5 Hz, 1H, CH), 8.42–8.27 (m, 4H, Ar-H), 8.59 (s, 1H, Ar-H).^13^C-NMR (100 MHz, CDCl_3_) δ (ppm): 11.58 (CH_3_), 25.09 (CH_3_), 120.36 (2CH), 122.80 (CH), 124.64 (CH), 125.79 (CH), 126.35 (CH), 129.25 (2CH), 129.97 (CH), 131.89 (CH), 134.38 (C), 136.93 (C), 138.73 (C), 141.06 (C), 142.67 (C), 143.36 (C), 144.98 (C), 148.97 (C), 155.25 (C), 189.29 (C=O). Anal. calcd. For C_22_H_17_N_5_O_3_ (399.40): C, 66.16; H, 4.29; N, 17.53. Found: C, 66.24; H, 4.17; N, 17.66%.

*3,6-Dimethyl-5-(4-cyanobenzylideneacetyl)-1-phenyl-1H-pyrazolo[3,4-b]pyrazine* (**25f**): Yield 0.16 g (80%), m.p. 254–256 °C. IR (ν, cm^−1^): 3079 (CH arom.), 2224 (CN), 1672 (C=O). ^1^H-NMR (400 MHz, CDCl_3_) δ (ppm): 2.84 (s, 3H, CH_3_), 3.09 (s, 3H, CH_3_), 7.37 (t, 1H, Ar-H), 7.57 (d, *J* = 7.4 Hz, 2H, CH), 7.81 (m, 6H, Ar-H), 8.39–8.30 (d, 2H, Ar-H). ^13^C-NMR (100 MHz, CDCl_3_) δ (ppm): 11.50 (CH_3_), 25.10 (CH_3_), 113.45 (C),118.46 (C≡N), 120.32 (2CH), 126.16 (CH), 126.94 (CH), 128.94 (CH), 129.24 (2CH), 130.02 (CH), 131.85 (CH), 132.66 (CH), 138.77 (C), 139.45 (C), 140.34 (C), 141.42 (C), 142.64 (C), 143.32 (C), 144.89 (CH), 155.28 (C), 189.27 (C=O). Anal. calcd. for C_23_H_17_N_5_O (379.41): C, 72.81; H, 4.52; N, 18.46. Found: C, 72.76; H, 4.40; N, 18.55%*.*

*3,6-Dimethyl-5-(4-methoxybenzylideneacetyl)-1-phenyl-1H-pyrazolo[3,4-b]pyrazine* (**25g**): Yield 0.18 g (90%), m.p. 210–212 °C. IR (ν, cm^−1^): 3069 (CH arom.), 2934, 2836 (CH aliph.), 1663 (C=O); ^1^H-NMR (400 MHz, CDCl_3_) δ (ppm): 2.82 (s, 3H, CH_3_), 3.05 (s, 3H, CH_3_), 3.89 (s, 3H, CH_3_), 6.98 (d, 2H, Ar-H), 7.34 (t, 1H, Ar-H), 7.56 (t, 2H, Ar-H), 7.69 (d, 2H, Ar-H), 7.84 (d, 1H, CH), 8.00 (d, *J* = 15.9 Hz, 1H, CH), 8.34 (d, 2H, Ar-H). ^13^C-NMR (100 MHz, CDCl_3_) δ (ppm): 11.49 (CH_3_), 24.85 (CH_3_), 55.41 (CH_3_), 114.40 (2CH), 120.23 (2CH), 121.02 (C), 126.13 (CH), 127.81 (CH), 129.19 (2CH), 130.52 (2CH), 131 (C), 138.92 (C), 142.67 (C), 144.47 (CH), 144.59 (C), 144.71 (C), 154.59 (C), 161.76 (C), 190.26 (C=O). Anal. calcd. for C_23_H_20_N_4_O_2_ (384.43): C, 71.86; H, 5.24; N, 14.57. Found: C, 71.75; H, 5.33; N, 14.63%.

*3,6-Dimethyl-5-(4-N,N-dimethylaminobenzylideneacetyl)-1-phenyl-1H-pyrazolo[3,4-b]pyrazine* (**25h**): Yield 0.14 g (70%), m.p. 227–229 °C. IR (ν, cm^−1^): 2921 (CH aliph.), 1659 (C=O); ^1^H-NMR (400 MHz, CDCl_3_) δ (ppm): 2.81 (s, 3H, CH_3_), 3.03 (s, 3H, CH_3_), 3.08 (s, 6H, 2CH_3_), 6.79 (d, 2H, Ar-H), 7.38–7.25 (t, 1H, Ar-H), 7.69–7.50 (m, 4H, CH, Ar-H), 7.83 (s, 2H, Ar-H), 8.34 (d, 2H, phenyl). ^13^C-NMR (100 MHz, CDCl_3_) δ (ppm): 11.48 (CH_3_), 24.64 (CH_3_), 40.08 (2CH_3_), 111.78 (2C), 118.36 (C), 120.17 (2CH), 122.77 (CH), 126.02 (CH), 129.16 (2CH), 130.75 (2CH), 131.57 (C), 139.06 (C), 142.81 (C), 144.61 (C), 146.06 (2C), 152.12 (CH), 154.100 (C), 190.60 (C=O). Anal. calcd. For C_24_H_23_N_5_O (397.47): C, 72.52; H, 5.83; N, 17.62. Found: C, 72.63; H, 5.94; N, 17.45%.

*3,6-Dimethyl-5-(4-chlorobenzylideneacetyl)-1-phenyl-1H-pyrazolo[3,4-b]pyrazine* (**25i**): Yield 0.23 g (92%), m.p. 228–230 °C; IR (ν, cm^−1^): 3081 (CH arom.), 1669 (C=O); ^1^H-NMR (400 MHz, CDCl_3_) δ (ppm): 2.82 (s, 3H, CH_3_), 3.07 (s, 3H, CH_3_), 7.33–7.37 (t, 1H, Ar-H), 7.43–7.45 (d, 2H, Ar-H), 7.54–7.58 (t, 2H, Ar-H), 7.65–7.67 (d, 2H, Ar-H), 7.81–7.85 (d, *J* = 16.0 Hz, 1H, CH), 8.14–8.18 (d, *J* = 16.0 Hz, 1H, CH), 8.32–8.34 (d, 2H, Ar-H).^13^C-NMR (100 MHz, CDCl_3_) δ (ppm): 11.50 (CH_3_), 24.98 (CH_3_), 120.31 (2CH), 123.61 (CH), 126.26 (CH), 129.23 (2CH), 129.63 (2CH), 129.86 (2CH), 131.77 (C), 133.60 (C), 136.46 (C), 138.85 (C), 142.67 (C), 142.85 (C), 43.92 (C), 144.82 (CH), 154.96 (C), 189.83 (C). Anal. calcd. for C_22_H_17_ClN_4_O (388.85): C, 67.95; H, 4.41; Cl, 9.12; N, 14.41. Found: C, 67.8; H, 4.55; Cl, 9.07; N, 14.54%.

*3,6-Dimethyl-5-(3,4-Dimethoxybenzylideneacetyl)-1-phenyl-1H-pyrazolo[3,4-b]pyrazine* (**25j**): Yield 0.17 g (85%), m.p. 199–201 °C. IR (ν, cm^−1^): 3078 (CH arom.), 2988, 2837 (CH aliph.), 1663 (C=O); ^1^H-NMR (400 MHz, CDCl_3_) δ (ppm): 2.82 (s, 3H, CH_3_), 3.05 (s, 3H, CH_3_), 3.98 (d, *J* = 9.8 Hz, 6H, 2CH_3_), 6.95 (d, *J* = 8.2 Hz, 1H, CH), 7.40–7.24 (m, 3H, CH, Ar-H), 7.56 (t, 2H, Ar-H), 7.82 (d, 1H, Ar-H), 7.96 (d, 1H, Ar-H), 8.34 (d, 2H, Ar-H). Anal. calcd. for C_24_H_22_N_4_O_3_ (414.46): C, 69.55; H, 5.35; N, 13.52. Found: C, 69.43; H, 5.47; N, 13.67%. No ^13^C-NMR data could be obtained due to the precipitation of the compound while running the measurement.

*3,6-Dimethyl-5-(Ethene-2-ylbenzylideneacetyl)-1-phenyl-1H-pyrazolo[3,4-b]pyrazine* (**25k**): Yield 0.17 g (85%), m.p. 211–213 °C, IR (ν, cm^−1^): 2922 (CH aliph.), 1662 (C=O); ^1^H-NMR (400 MHz, CDCl_3_) δ (ppm): 2.81 (s, 3H, CH_3_), 3.05 (s, 3H, CH), 7.13 (m, 2H, CH, Ar-H), 7.43–7.33 (m, 4H, CH, Ar-H), 7.55 (d, 4H, CH, Ar-H), 7.70 (t, 2H, Ar-H), 8.33 (d, 2H, Ar-H). ^13^C-NMR (100 MHz, CDCl_3_) δ (ppm): 11.45 (CH_3_), 24.88 (CH_3_), 120.32 (2CH), 126.20 (CH), 126.84 (CH), 127.35 (2CH), 128.86 (2CH), 129.22 (2CH), 136.20 (CH), 138.90 (C), 142.02 (CH, C), 144.29 (2C), 144.58 (CH, C),144.78 (CH, C), 154.72 (C), 189.32 (C=O). Anal. calcd. for C_24_H_20_N_4_O for (380.44): C, 75.77; H, 5.30; N, 14.73. Found: C, 75.83; H, 5.18; N, 14.66%.

*3,6-Dimethyl-5-(5-phenyl-4,5-dihydro-1H-pyrazol-3-yl)-1-phenyl-1H-pyrazolo[3,4-b]pyrazine* (**26**): A mixture of chalcone **25a** (0.25 g, 7 mmol), hydrazine hydrate (80%, 0.3 mL), in ethanol (15 mL) was heated under reflux for 10 h. After cooling the solid product obtained was filtered, washed with water, and recrystallized from dioxane-water mixture (3:1) as yellow crystals, yield 0.23 g (50%), m.p. 146–148 °C. IR (ν, cm^−1^): 3351 (NH), 2921 (CH aliph.). ^1^H-NMR (400 MHz, CDCl_3_) δ (ppm): 2.63 (s, 3H, CH_3_), 3.10 (s, 3H, CH_3_), 3.73 (s, 3H, CH, CH_2_), 5.08 (s, 1H, NH), 7.33 (m, 3H, phenyl), 7.45–7.37 (m, 3H, phenyl), 7.55 (t, 2H, phenyl), 8.32 (d, 2H, phenyl). ^13^C-NMR (100 MHz, CDCl_3_) δ (ppm): 11.27 (CH_3_), 25.00 (CH_3_), 43.00 (CH_2_), 67.10 (CH), 120.04 (2CH), 125.69 (2CH), 126.3 (2CH), 128.86 (2CH), 129.1 (2CH), 129.8 (C), 131 (C), 139.7 (C), 142 (C), 143.3 (C), 143.5 (C), 144.5 (C), 154 (C). Anal. calcd. for C_22_H_20_N_6_ (368.43): C, 71.72; H, 5.47; N, 22.81. Found: C, 71.63; H, 5.53; N, 22.60%.

*3,6-Dimethyl-5-(5-phenyl-4,5-dihydro-1-phenyl-1H-pyrazol-3-yl)-1-phenyl-1H-pyrazolo[3,4-b]pyrazine* (**27**): A mixture of chalcone **25a** (0.25 g, 7 mmol), phenyl hydrazine (0.1 g, 7 mmol) in ethanol (15 mL) was heated under reflux for 15 min. After cooling, the reaction mixture was filtered, washed with water, and recrystallized from ethanol as yellow crystals, yield 0.15 g (62%), m.p. 162–164 °C. IR (ν, cm^−1^): 2900 (CH aliph.); ^1^H-NMR (400 MHz, CDCl_3_) δ (ppm): 2.81 (s, 3H, CH_3_), 3.06 (s, 3H, CH_3_), 3.70 (t, 1H, CH), 7.34–7.44 (d, 2H, phenyl), 7.45–7.46 (t, 2H, phenyl), 7.52–7.58 (t, 2H, phenyl), 7.71–7.74 (m, 6H, phenyl), 7.85 (d, 1H, CH_2_), 8.15 (d, 1H, CH_2_), 8.30–8.33 (d, 3H, phenyl). ^13^C-NMR (100 MHz, CDCl_3_) δ (ppm): 11.50 (CH_3_), 24.93 (CH_3_), 38.13 (CH_2_), 59.51 (CH), 102.18 (C), 120.33 (2CH), 123.25 (2CH), 126.23 (2CH), 128.73 (2CH), 128.94 (2CH), 129.23 (2CH), 130.60 (2CH), 131.75 (CH), 135.06 (C), 138.86 (C), 142.68 (C), 144.20 (C), 144.55 (2C), 144.82 (C), 154.82 (C). MS: *m*/*z* (443 M^+^-1). Anal. calcd. for C_28_H_24_N_6_ (444.5): C, 75.65; H, 5.44; N, 18.91. Found: C, 75.41; H, 5.21; N, 19.20%.

*3,6-Dimethyl-5-(5-phenyl-4,5-dihydro*[1,2]*oxazol-3-yl)-1-phenyl-1H-pyrazolo[3,4-b]pyrazine* (**28**): A mixture of chalcone **25a** (0.25 g, 7 mmol), hydroxylamine hydrochloride (0.1 g, 7 mmol), and sodium acetate (0.3 g, 2 mmol) in ethanol (10 mL) was heated under reflux for 6 h. The reaction mixture was then evaporated to half of its volume. After cooling, it was neutralized with dil. HCl and the solid precipitate was filtered off, washed with water, and recrystallized from ethanol-water mixture (1:1), yield 0.17 g (74%), m.p. 200–202 °C. IR (ν, cm^−1^): 2926 (CH aliph.). ^1^H-NMR (400 MHz, CDCl_3_) δ (ppm): 2.69 (s, 3H, CH_3_), 2.73 (s, 3H, CH_3_), 2.79 (d, 1H, CH_2_), 3.14 (d, 1H, CH_2_), 3.72–3.77 (t, 1H, CH), 7.28–8.33 (m, 10H, Ar-H). ^13^C-NMR (100 MHz, CDCl_3_) δ (ppm): 11.38 (CH_3_), 23.15 (CH_3_), 46.08 (CH_2_), 102.88 (CH cyclic), 106.66 (C), 120.33 (2CH), 123 (CH), 125.95 (CH), 126.17 (CH), 126.95 (CH), 128.71 (CH), 128.92 (CH), 129.00 (CH), 129.19 (CH), 130 (C), 139.01 (C), 144.53 (C), 151.42 (C), 156.52 (C), 162.43 (C), 169.64 (C). Anal. calcd. for C_22_H_19_N_5_O (369.42): C, 71.53; H, 5.18; N, 18.96. Found: C, 71.41; H, 5.21; N, 18.91%.

*5-Phenyl-4,5-dihydro-3-(3,6-dimethyl-1-phenyl-1H-pyrazolo[3,4-b]pyrazine-5-yl) pyrazole-1-carbothioamide* (**29**): A mixture of compound **25a** (0.25 g, 7 mmol) and thiosemicarbazide (0.1 g, 7 mmol) in ethanolic NaOH (25%, 10 mL) was heated under reflux for 3 h. After cooling, the reaction mixture was filtered, washed with water, and recrystallized from ethanol-dioxane mixture (1:1) as yellow crystals, yield 0.08 g (50%), m.p. 234–236 °C. IR (ν, cm^−1^): 3407, 3280 (NH_2_), 3165 (CH arom.), 2851 (CH aliph.). ^1^H-NMR (400 MHz, CDCl_3_) δ (ppm): 2.67 (s, 3H, CH_3_), 2.70 (s, 3H, CH_3_), 3.09–3.22 (dd, *J* = 27.5, 21.8 Hz, 2H), 7.03 (s, 1H, CH), 7.31–7.39 (m, 6H, phenyl), 7.52–7.60 (m, 2H, phenyl), 8.30–8.40 (d, 2H, phenyl), 9.06 (2H, NH_2_). ^13^C-NMR (100 MHz, CDCl_3_) δ (ppm): 11.48 (CH_3_), 26.86 (CH_3_), 44.7(cyclic CH_2_), 66.85 (CH cyclic), 120.25 (2CH), 125.7 (CH, C), 126.5 (2CH), 127.4 (CH, C), 129.02 (2CH), 129.8 (2CH), 131 (C), 138 (C), 141.60 (C), 155.80 (C), 156.27 (C), 165 (C), 178 (C=S)**.** Anal. calcd. for C_23_H_19_N_7_S (425.5): C, 64.92; H, 4.50; N, 23.04. Found: C, 64.94; H, 4.44; N, 23.30%.

*2-Amino-4-(4-phenyl)-6-(3,6-dimethyl-1-phenyl-1H-pyrazolo[3,4-b]pyrazine-5-yl)pyrimidine* (**30**): A mixture of chalcone **25a** (7 mmol), guanidine sulfate (0.12 g, 7 mmol) in ethanolic KOH (30%, 5 mL) was heated under reflux for 3–4 h. The reaction mixture was then evaporated to half volume and after cooling it was neutralized with dil. HCl. The solid precipitate was filtered off, washed with water, and recrystallized from dioxane-acetone mixture (1:3) as yellow crystals, yield 0.1 g (40%), m.p. 150–152 °C. IR (ν, cm^−1^): 3302, 3182 (NH_2_), 3061.37 (CH arom.), 2919, 2849 (CH aliph.).^1^H-NMR (400 MHz, CDCl_3_) δ (ppm): 2.77 (s, 3H, CH_3_), 2.99 (s, 3H, CH_3_), 5.27 (d, 2H, NH_2_), 7.34 (t, 3H, phenyl), 7.53 (t, 3H, phenyl), 7.67 (s, 1H, CH), 8.23 (d, 2H, phenyl), 8.34–8.36 (d, 2H, phenyl). ^13^C-NMR (100 MHz, CDCl_3_) δ (ppm): 11.11 (CH_3_), 24.58 (CH_3_), 107.81 (C), 119.92 (CH), 120.21 (2CH), 125.86 (CH), 127.22 (2CH), 128.77 (2CH), 129.14 (2CH), 130.66 (CH), 132.31 (C), 137.40 (C), 139.23 (C), 142.91 (C), 144.02 (C), 146.52 (C), 152.54 (C), 162.87 (C), 166.64 (C). Anal. calcd. for C_23_H_21_N_7_ (395): C, 69.85; H, 5.35; N, 24.79. Found: C, 69.63; H, 5.44; N, 24.60%.

### 3.2. Biological Evaluations

#### 3.2.1. Anti-Inflammatory Assay

Adult albino rats, weighing 150–200 g, were divided into ten groups, each of three animals. The animals were allowed food and water *ad libitum*, except during the experiment. They were housed in a room at 23 ± 2C with a 12 h light/dark cycle. All the compounds under test and the indomethacin reference drug [30], in 3% Tween 80 solution in normal saline as a vehicle, were administered at a dose of 28 μM/kg bodyweight, while control group received only a vehicle. The difference between the thicknesses of the paw at zero time and different time intervals after injection was taken as a measure of edema. The measurement was carried out at 0.5, 1, 2, 3, and 4 h after injection of the tested compounds, reference drug, and control. The right paw volume was measured using a digital plethysmometer (Model 7150, Ugo Basile, Varese, Italy), directly before and after carrageenan injection to detect the carrageenan induced inflammation. 

#### 3.2.2. Cytotoxicassay

A number of 1 × 10^4^ cells/well were cultured in 96-well plates and then incubated at 37 °C, under conditions of 5% CO_2_ and 95% air for 24 h. On the following day, the compounds under test, diluted to the desired concentrations, were added to the wells with the respective negative control and then the cells were incubated for 24 h. Next, 50 μL of MTT (3-(4,5-dimethylthiazol-2yl)-2,5-diphenyl tetrazolium bromide) solution (2 mg/mL of MTT in PBS) was added to each well and left for 3–4 h. Formazan crystals formed after incubation were dissolved in 150 μL DMSO were added to each well. The 96-well plates were shaken for 10 min and then were read at A570 with reference filter at A650 using Elisa plate reader. Paclitaxel was used as a positive control.

## 4. Conclusions

In conclusion, several new derivatives of pyrazolo[3,4-*b*]pyrazines and related heterocycles were prepared and evaluated for their anti-inflammatory and anti-breast cancer MCF-7 cell line activities. Compounds **15** and **29** showed the higher anti-inflammatory activity, where the potency of the former compound was comparable to that of the indomethacin reference drug (cf. Table 1). On the other hand, compounds **25i**, **25j** exhibited very significant anticancer activity against MCF-7 breast cancer cell line with respect to the positive control Paclitaxel (cf. Figure 1 and Figure 2).

## Figures and Tables

**Table 1:** Anti-inflammatory Activity of Tested Compounds (**15**, **25a**, **26**–**30**) against Acute Carrageenan-Induced Paw Oedema in Rats (Statistical analysis).

Compound ^a^		Paw Oedema Inhibition (Swell ± S.E.M.) ^a,b,c^ (%)					
	30 min	1 (h)	2 (h)	3 (h)	4 (h)	5 (h)	Potency ^d^
	Edema Induced by Carrageenan (% Edema Inhibition Relative to Control)						
**15**	0.40 ± 0.00	0.55 ± 0.03	0.48 ± 0.02	0.42 ± 0.02	0.38 ± 0.02	0.40 ± 0.00	44.44
**25a**	0.63 ± 0.03	0.66 ± 0.03	0.65 ± 0.03	0.66 ± 0.02	0.63 ± 0.02	0.63 ± 0.03	12.50
**26**	0.55 ± 0.03	0.68 ± 0.02	0.66 ± 0.02	0.62 ± 0.02	0.62 ± 0.02	0.55 ± 0.03	23.61
**27**	0.61 ± 0.02	0.68 ± 0.02	0.60 ± 0.03	0.60 ± 0.03	0.63 ± 0.02	0.62 ± 0.02	15.07
**28**	0.46 ± 0.02	0.63 ± 0.02	0.63 ± 0.02	0.60 ± 0.03	0.48 ± 0.02	0.47 ± 0.02	34.72
**29**	0.43 ± 0.00	0.45 ± 0.03	0.43 ± 0.02	0.42 ± 0.02	0.38 ± 0.03	0.43 ± 0.02	40.27
**30**	0.51 ± 0.02	0.61 ± 0.02	0.55 ± 0.05	0.45 ± 0.03	0.45 ± 0.03	0.52 ± 0.02	27.77
Drug *	0.40 ± 0.00	0.45 ± 0.03	0.42 ± 0.02	0.38 ± 0.0	0.37 ± 0.02	0.40 ± 0.00	44.44
**Negative Control**	0.72 ± 0.02	0.73 ± 0.02	0.73 ± 0.02	0.72 ± 0.02	0.73 ± 0.02	0.72 ± 0.02	-

(^a^) Dose 28 μM/kg. (^b^) *n* = 6. (^c^) Statistically significant from the indomethacin at *p* < 0.05. (^d^) Potency was expressed as % oedema inhibition of the tested compounds relative to % oedema inhibition of indomethacin (reference drug). * Drug = indomethacin (28 μM/kg).

## Supplementary Materials

The spectral data are available online Figures S1–S111.
