# Supplementary material for: Anticancer and Anti-Inflammatory Activities of Some New Pyrazolo[3,4-b]pyrazines
Source: Molecules. 2018 Oct 16;23(10):2657. doi: 10.3390/molecules23102657 (PMC6222508; doi:10.3390/molecules23102657)
Supplement: Supplementary file 1 [file molecules-23-02657-s001.pdf]

Anticancer and anti-inflammatory activities of some new pyrazolo[3,4-*b*]pyrazines

Hussein El-Kashef [1,*], Talaat El-Emary [1], Pierre Vehraeghe [2], Patrice Vanelle [3] and Maha Samy [1]

1 Chemistry Department, Faculty of Science, Assiut University,71516 Assiut, Egypt; elkashef@aun.edu.eg (H.E); emarytalaat@yahoo.com (T.E); maha11292@yahoo.com (M.S).
2 LCC-CNRS Université de Toulouse, CNRS, UPS, Toulouse, France; pierre.verhaeghe@lcc-toulouse.fr
3 Aix-Marseille Université, CNRS, Institut de Chimie Radicalaire ICR, UMR 7273, Laboratoire de Pharmaco-Chimie Radicalaire, Faculté de Pharmacie, 27 boulevard Jean Moulin CS 30064, Marseille Cedex 05, 13385, France; patrice.vanelle@univ-amu.fr
* Correspondence: elkashef@aun.edu.eg; Tel.: +20 1005075881

Supplementary Materials:

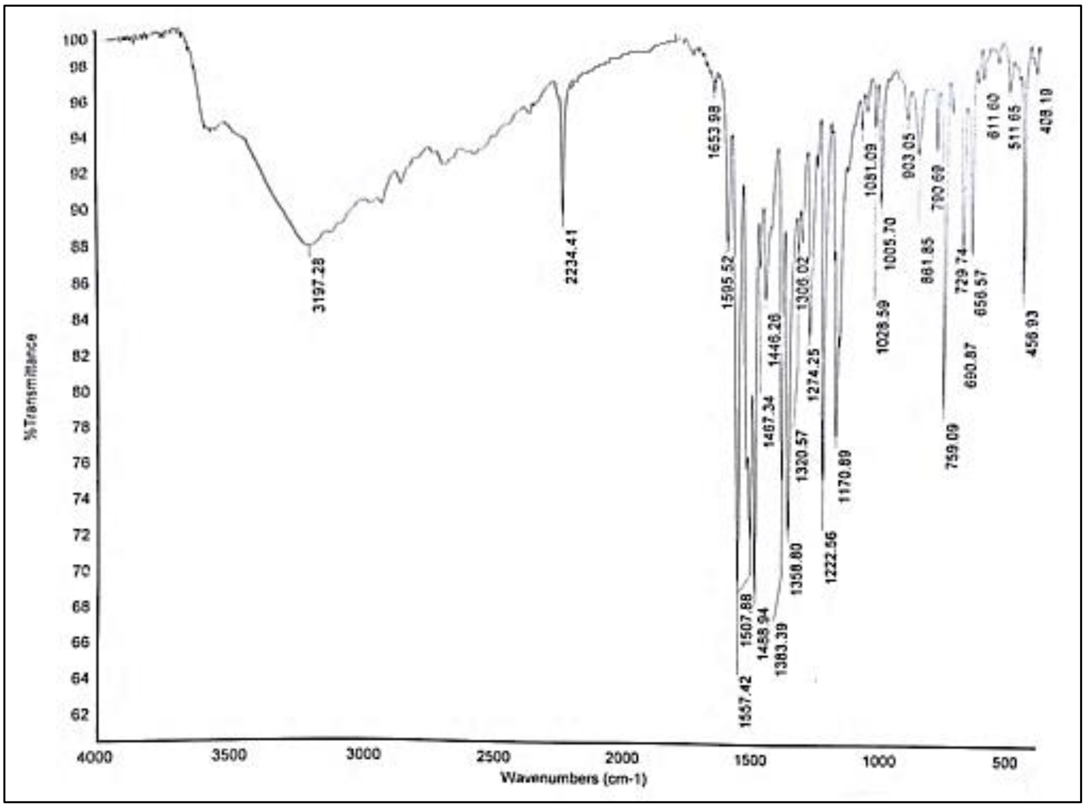

(Fig. 1), IR spectrum of *6-hydroxy-3-methyl-1-phenyl-1H-pyrazolo[3,4-b]pyrazine-5-carbonitrile* (**6a**)

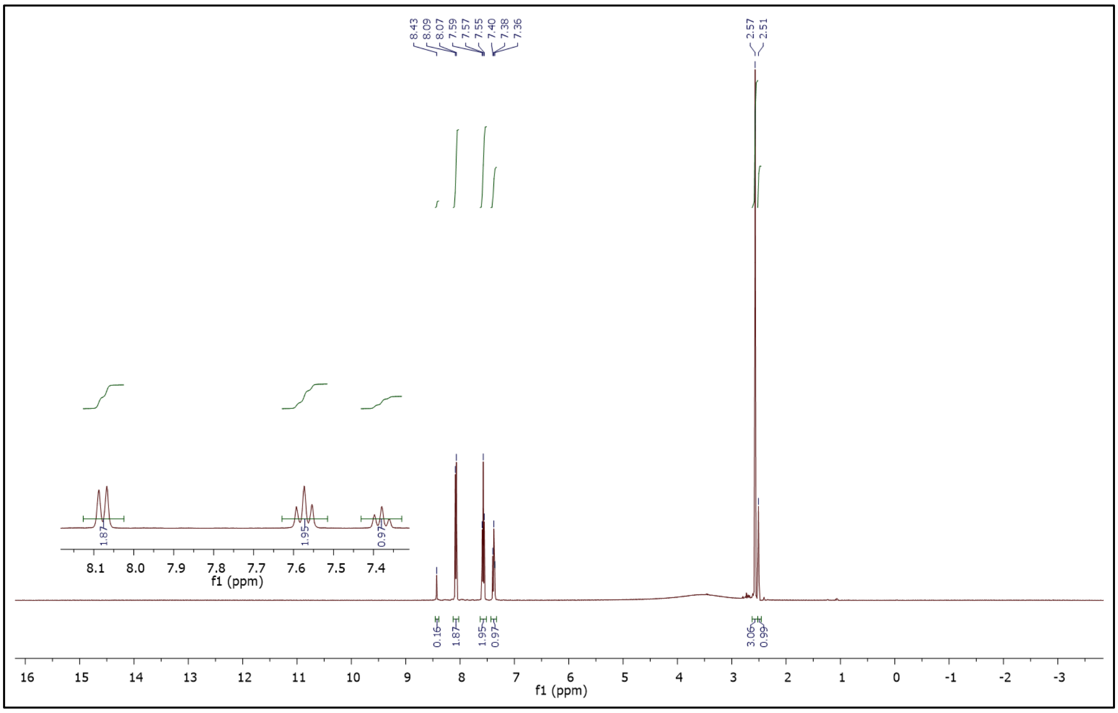

(Fig. 2), ^{1}H-NMR spectrum of *6-hydroxy-3-methyl-1-phenyl-1H-pyrazolo[3,4-b] pyrazine-5-carbonitrile* (**6a**). DMSO-d_6

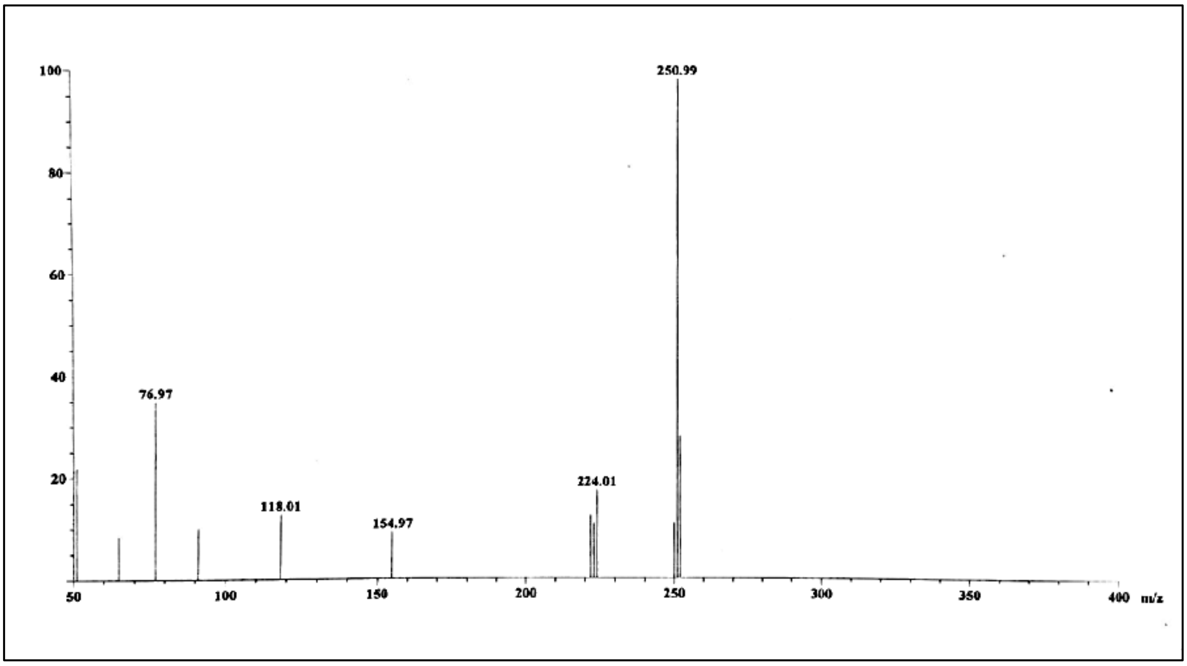

(Fig. 3), Mass spectrum of *6-hydroxy-3-methyl-1-phenyl-1H-pyrazolo[3,4-b]pyrazine-5-carbonitrile* (**6a**).

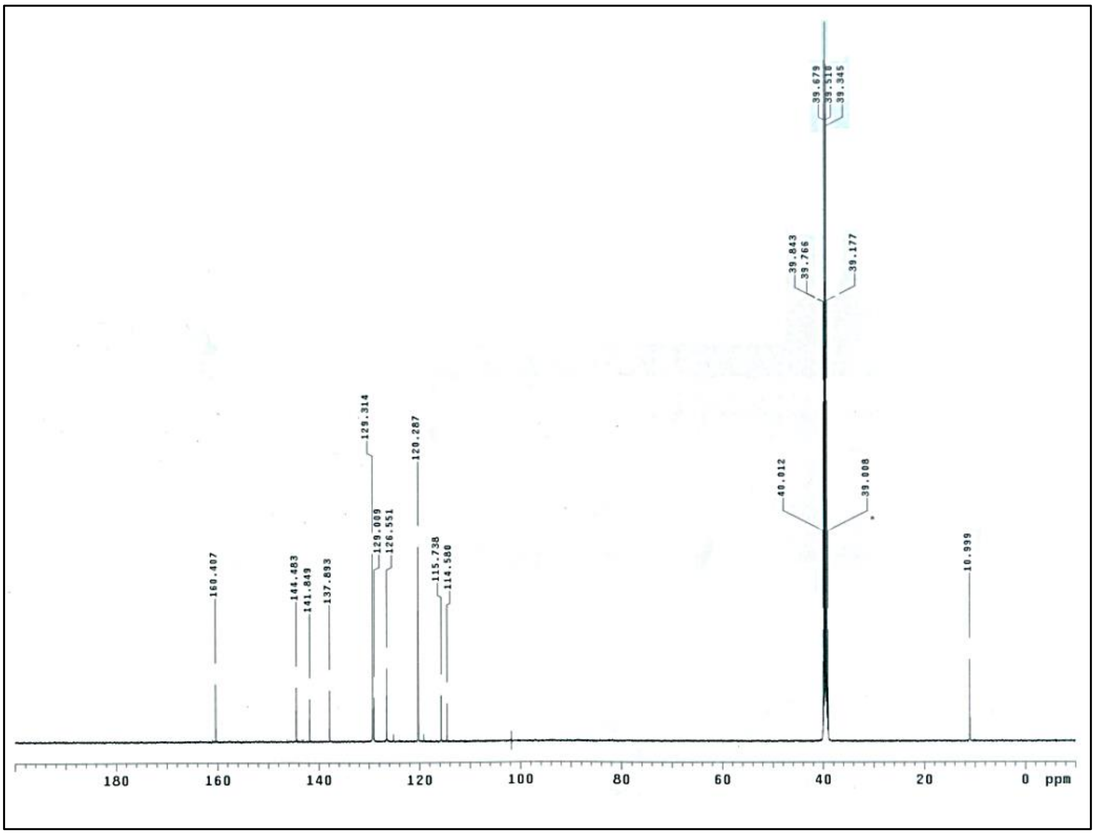

(Fig. 4), ^{13}C-NMR spectrum of *6-hydroxy-3-methyl-1-phenyl-1H-pyrazolo[3,4-b]pyrazine-5-carbonitrile* (**6a**). DMSO-d_6.

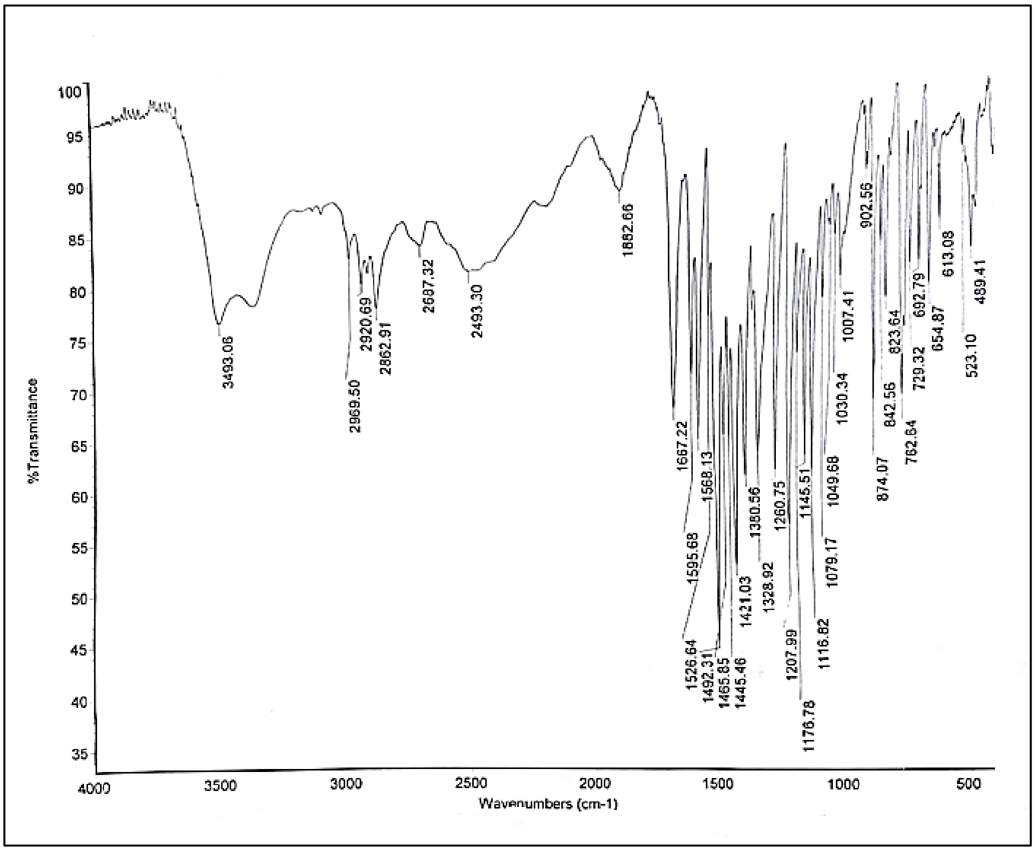

(Fig. 5), IR spectrum of 6-hydroxy-*3-methyl-1-phenyl-1H-pyrazolo[3,4-b]pyrazine-5-carboxylic acid* (**8**)

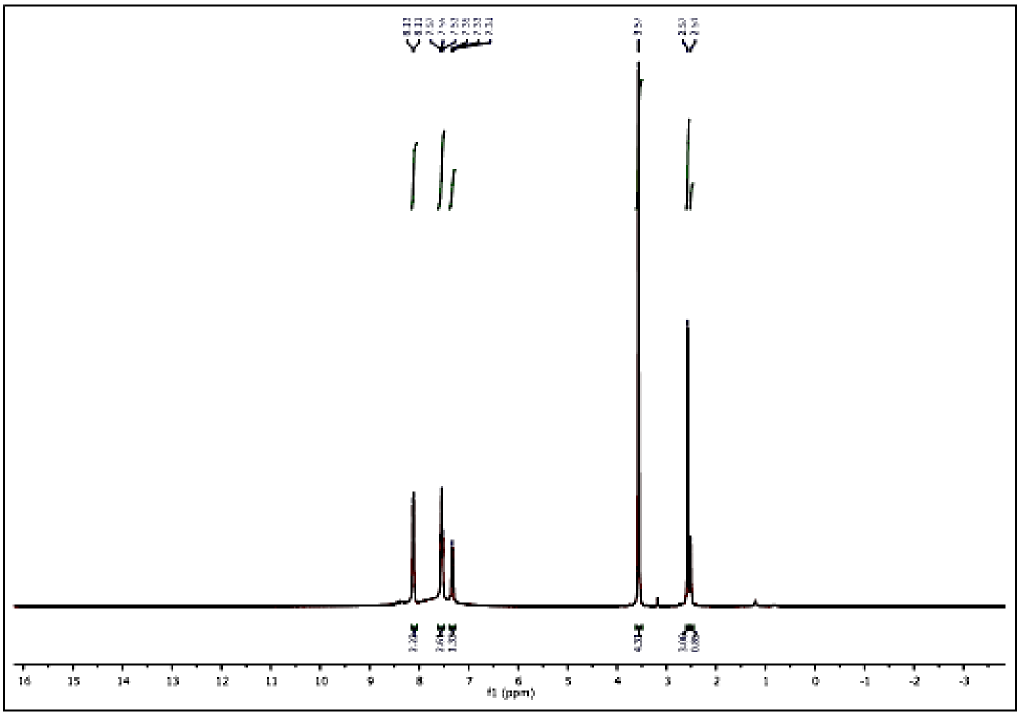

(Fig. 6), ^{1}H-NMR spectrum of 6-hydroxy-*3-methyl-1-phenyl-1H-pyrazolo[3,4 b]pyrazine-5-carboxylic acid* (**8**). DMSO-d_6.

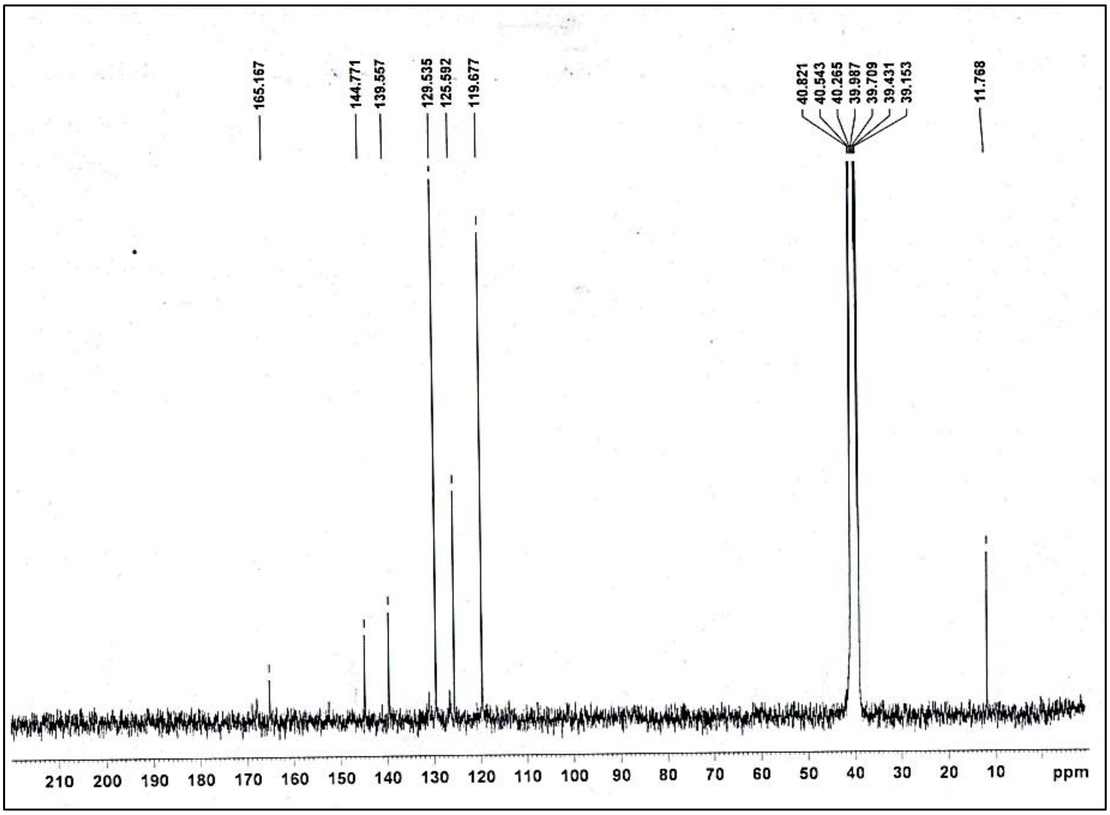

(Fig. 7), ^{13}C-NMR spectrum of 6-hydroxy-*3-methyl-1-phenyl-1H-pyrazolo[3,4-b]pyrazine-5-carboxylic acid* (**8**). DMSO-d_6.

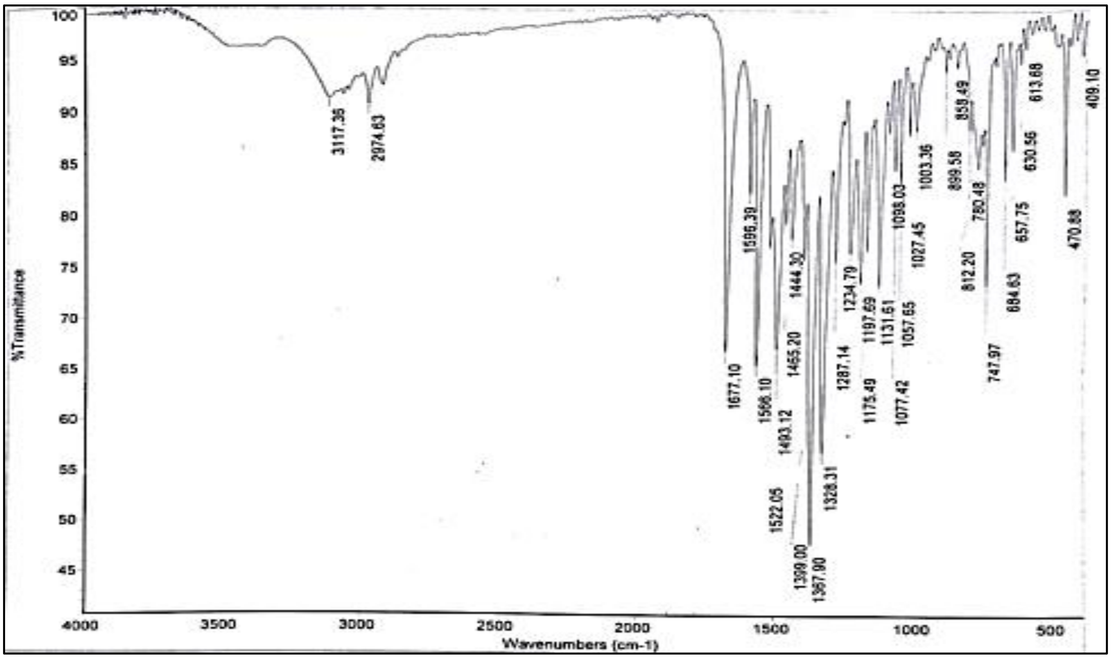

(Fig. 8), IR spectrum of *ethyl-6-hydroxy-3-methyl-1-phenyl-1H-pyrazolo[3,4-b]pyrazine-5-carboxylate (**9**).*

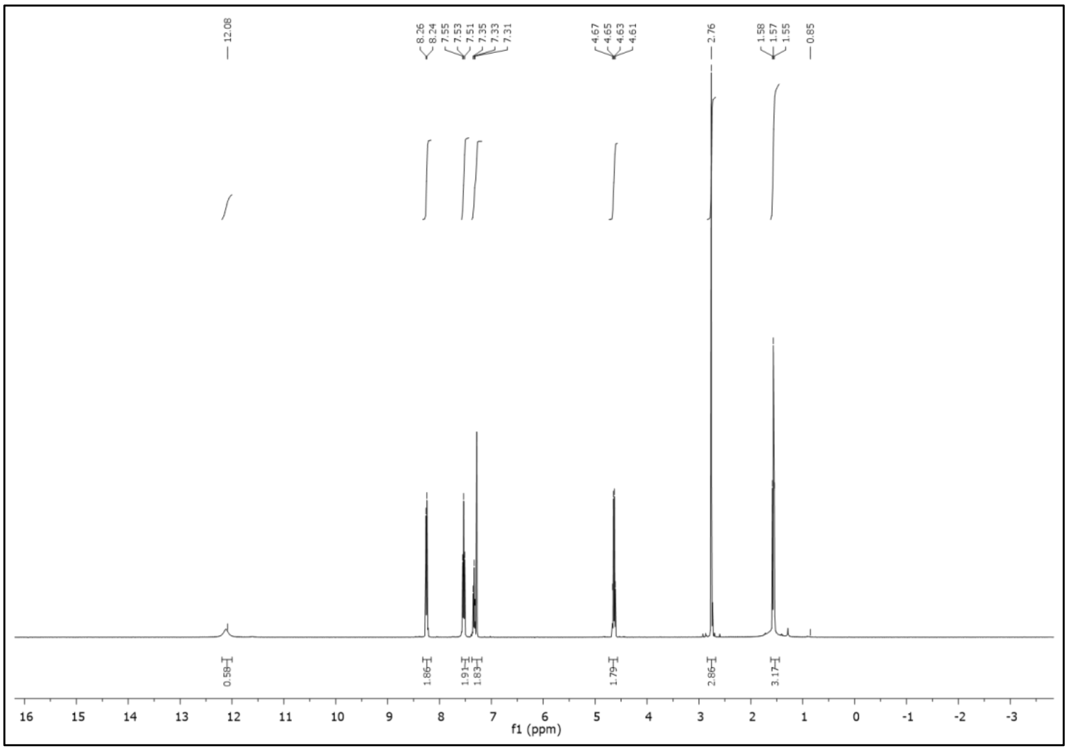

(Fig. 9), [1]*H-NMR spectrum of ethyl-6-hydroxy-3-methyl-1-phenyl-1H-pyrazolo[3,4-b]pyrazine-5-carboxylate (***9***).CDCl*$_3$

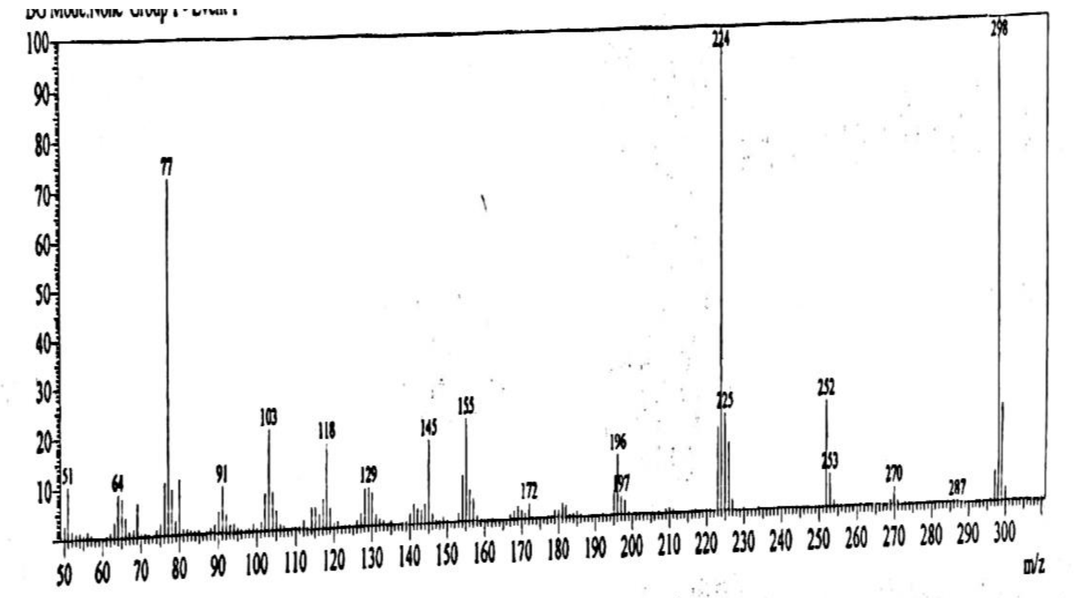

(Fig. 10), *Mass spectrum of ethyl-6-hydroxy-3-methyl-1-phenyl-1H-pyrazolo[3,4 b]pyrazine-5-carboxylate (***9***).*

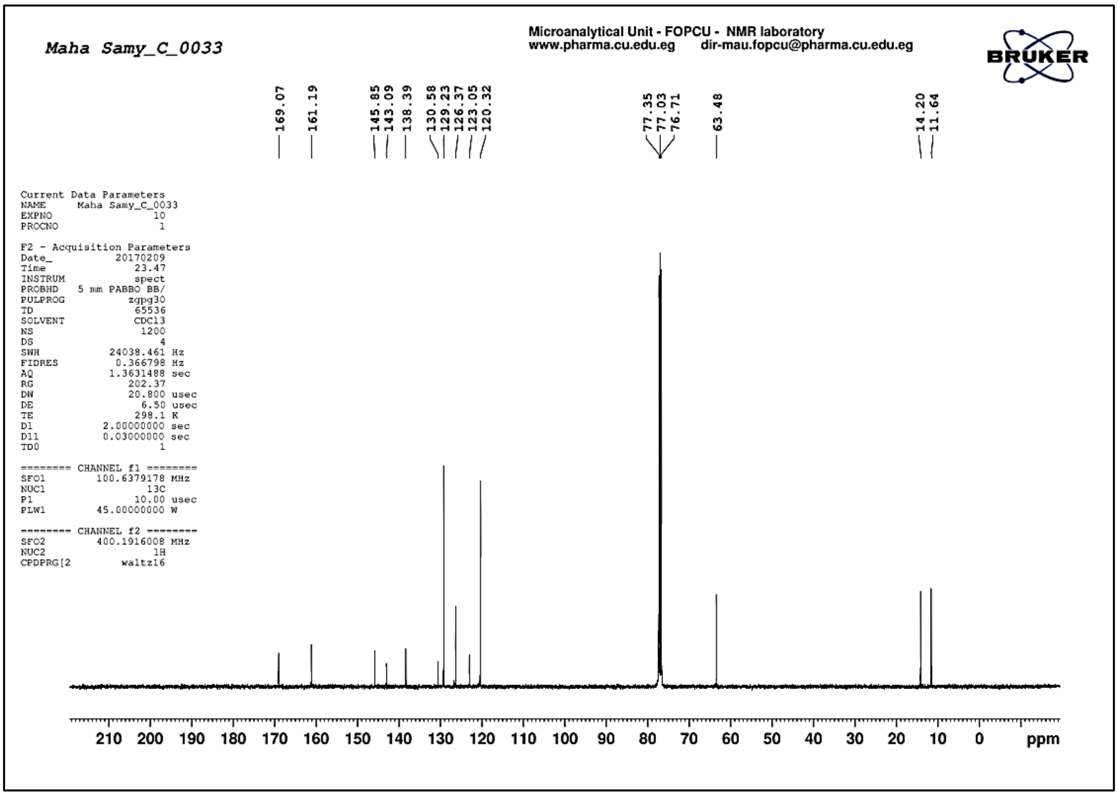

(Fig. 11), [13]*C-NMR spectrum of ethyl-6-hydroxy-3-methyl-1-phenyl-1H-pyrazolo[3,4-b]pyrazine-5-carboxylate (***9***).CDCl*$_3$

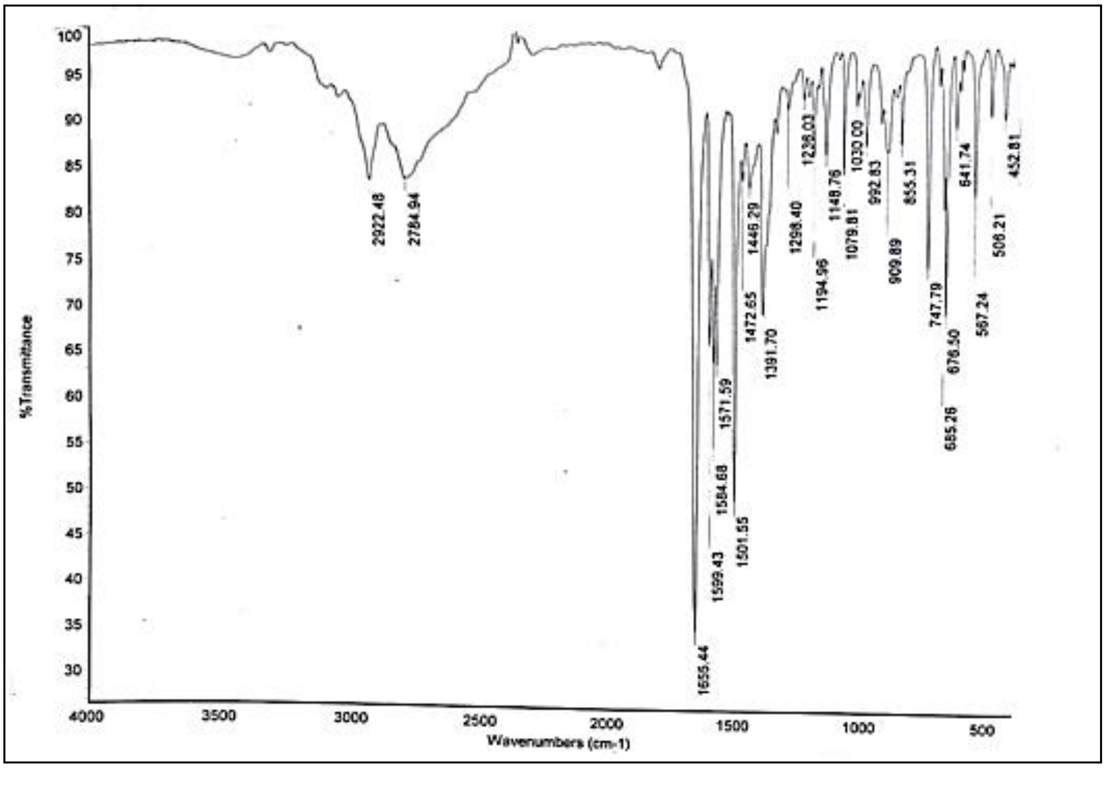

(Fig. 12), IR spectrum of 5-acetyl-3-methyl-1-phenyl-1*H*,6*H*-imidazo[4,5-*c*]pyrazole

(10).

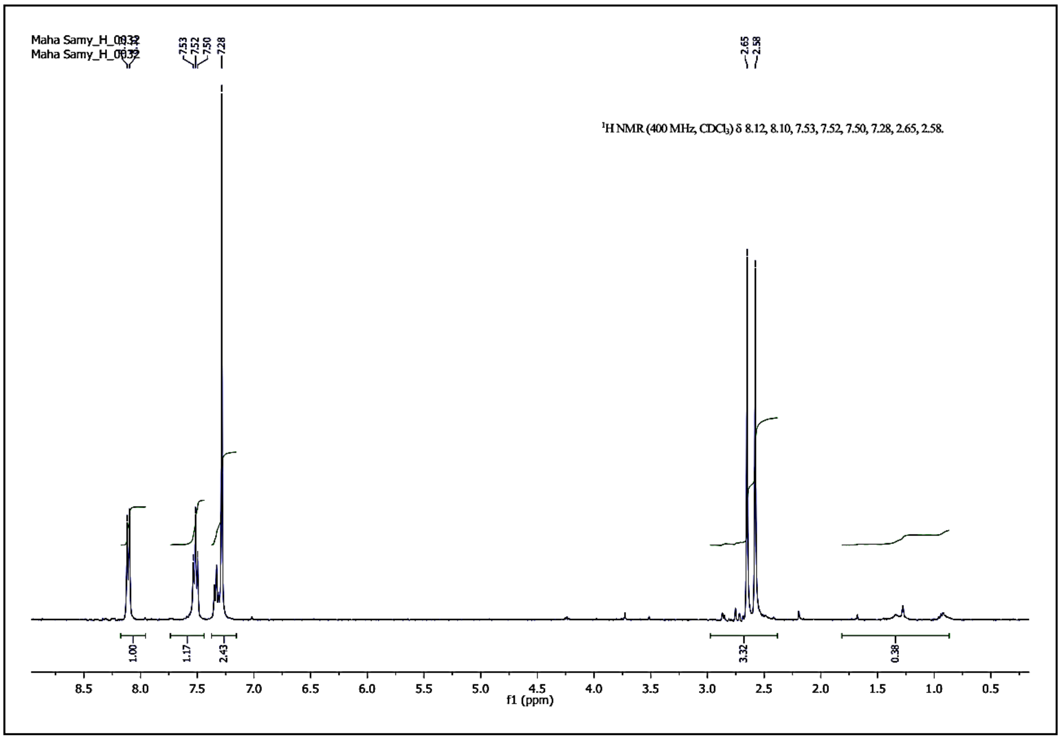

(Fig. 13), ^{1}H-NMR spectrum of 5-acetyl-3-methyl-1-phenyl-1*H*,6*H*-imidazo[4,5-*c*]pyrazole **(10)**. DMSO-d_6.

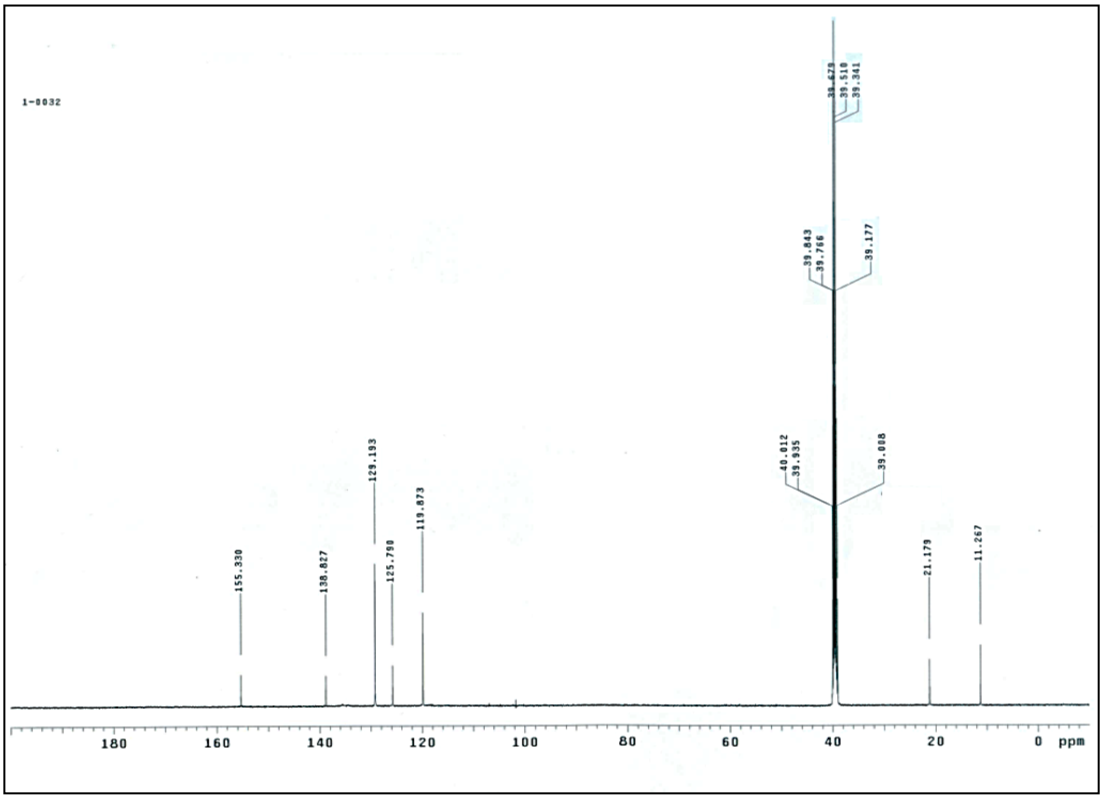

(Fig. 14), ^{13}C-NMR spectrum of 5-acetyl-3-methyl-1-phenyl-1*H*,6*H*-imidazo[4,5-*c*] pyrazole **(10)**. DMSO-d_6.

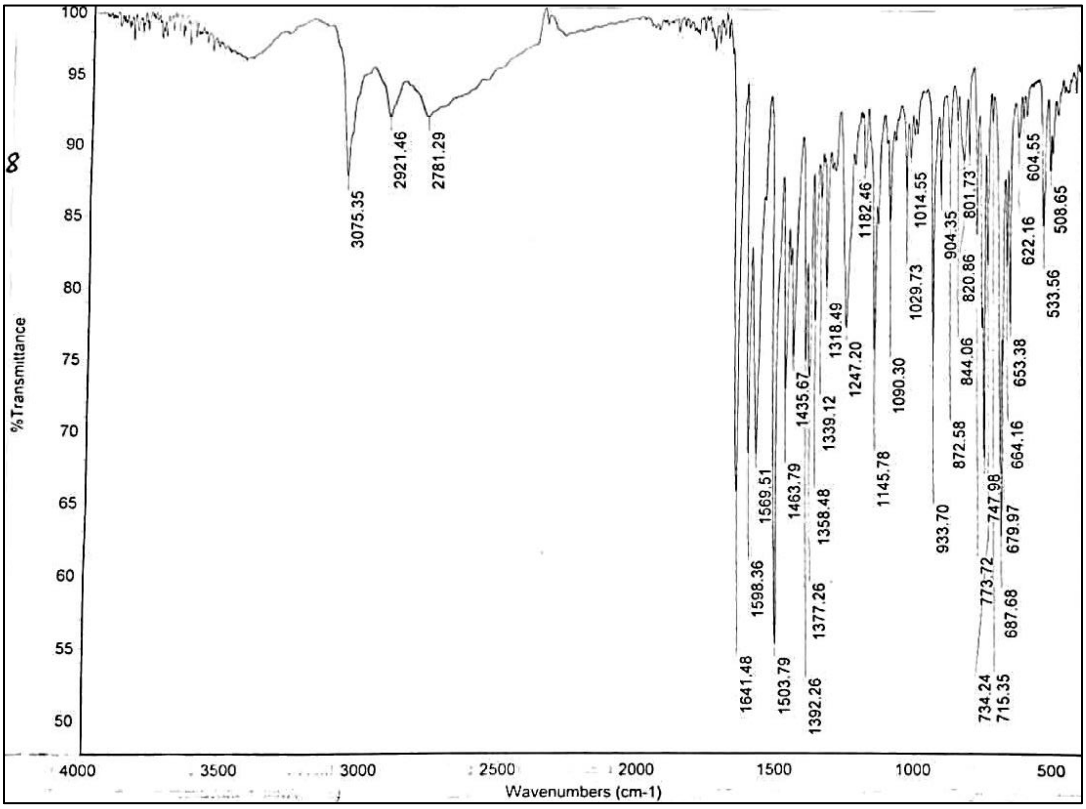

(Fig. 15), *IR spectrum of 5-benzoyl-3-methyl-1-phenyl-1H,6H-imidazo[4,5-c]pyrazole (**11**)*

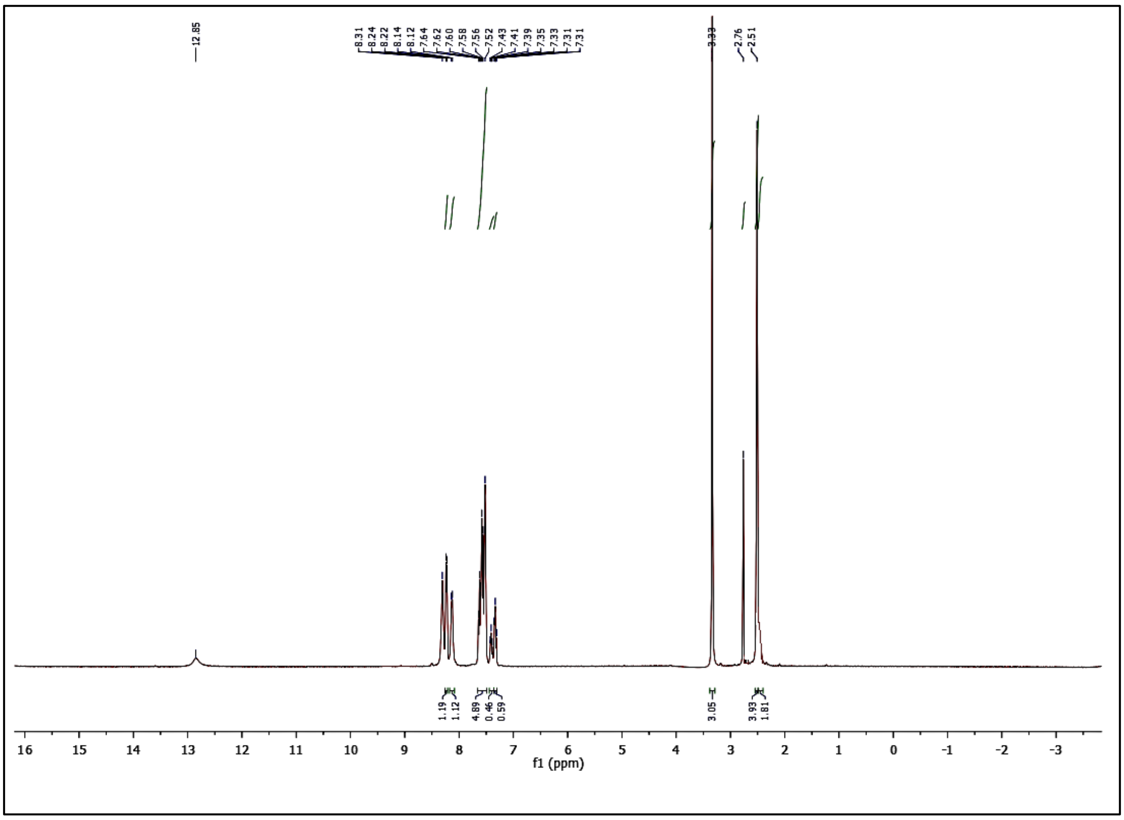

(Fig. 16), *^{1}H-NMR spectrum of 5-benzoyl-3-methyl-1-phenyl-1H,6H-imidazo[4,5-c] pyrazole (**11**). DMSO-d_6.*

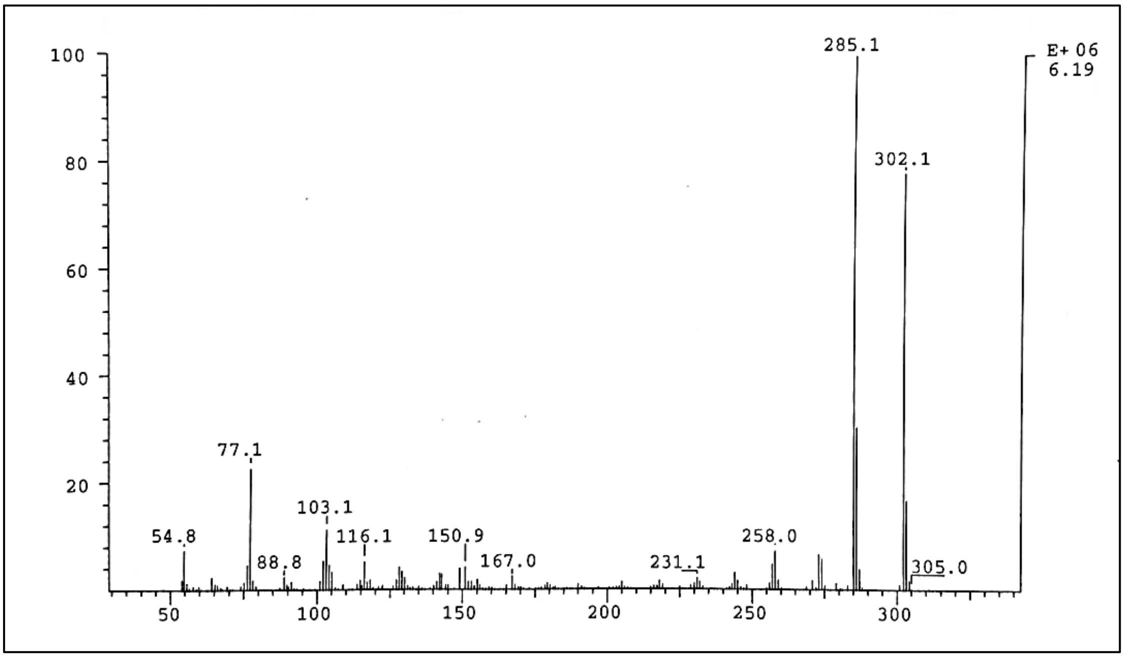

(Fig. 17), *Mass spectrum of 5-benzoyl-3-methyl-1-phenyl-1H,6H-imidazo[4,5-c]pyrazole (**11**).*

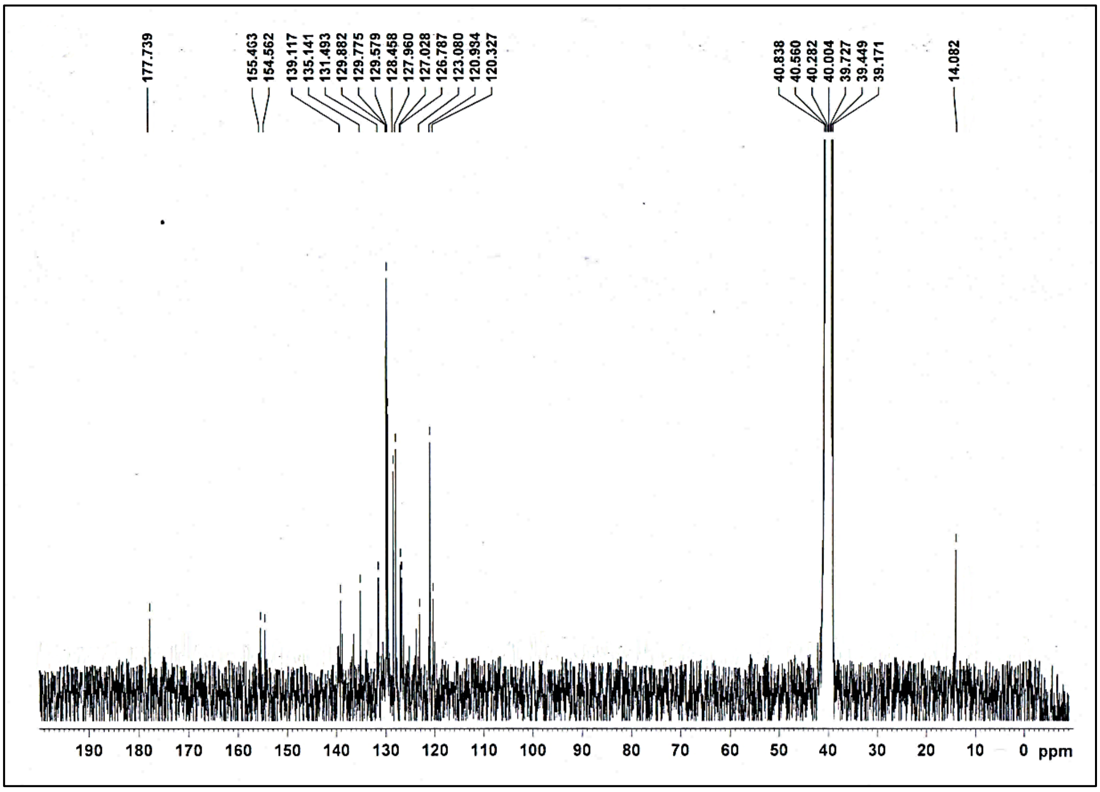

(Fig. 18), *^{13}C-NMR spectrum of 5-benzoyl-3-methyl-1-phenyl-1H,6H-imidazo[4,5-c] pyrazole (**11**). DMSO-d_6.*

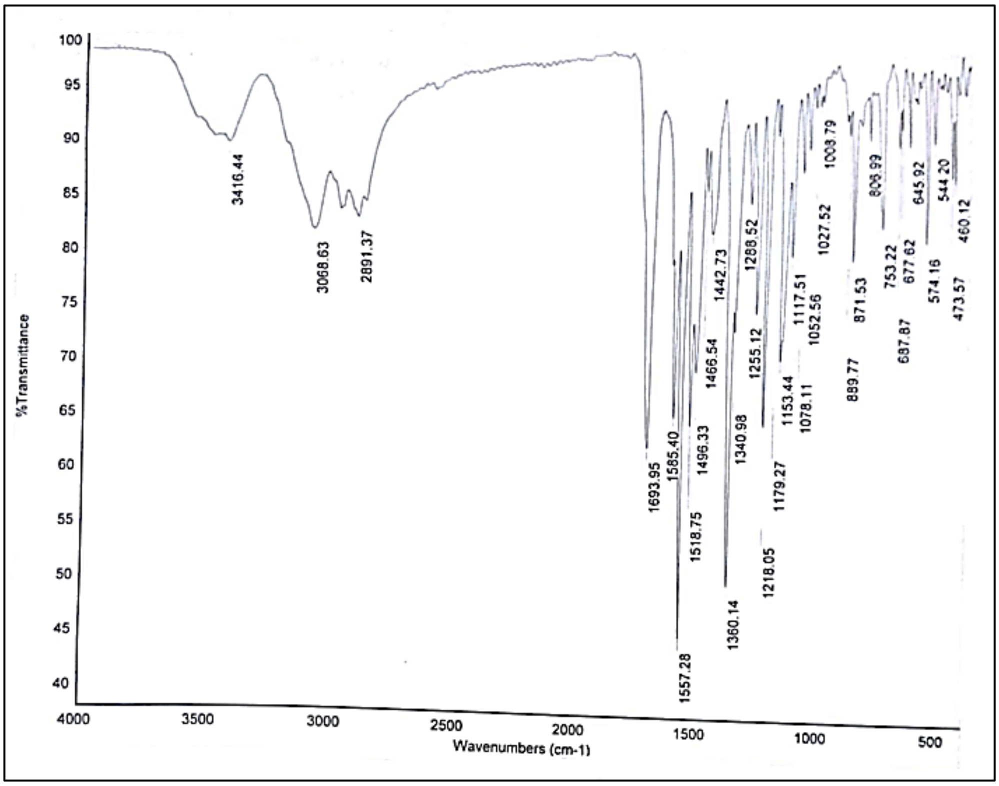

(Fig. 19), *IR spectrum of 3-methyl-1-phenyl-7-thioxo-7,8-dihydro-1H-pyrazolo[4,3-g] pteridin-5(6H)-one (**12**).DMSO-d*$_6$

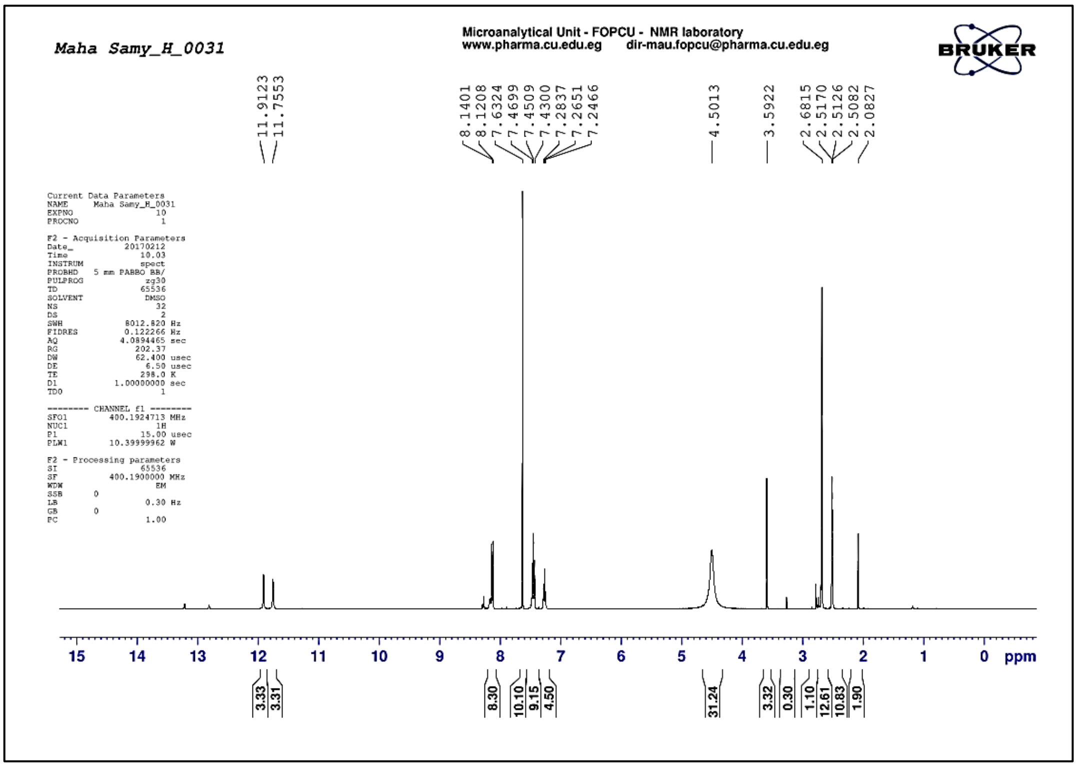

(Fig. 20), *^{13}C-NMR spectrum of 3-methyl-1-phenyl-7-thioxo-7,8-dihydro-1H-pyrazolo [4,3-g]pteridin-5(6H)-one (**12**).DMSO-d*$_6$.

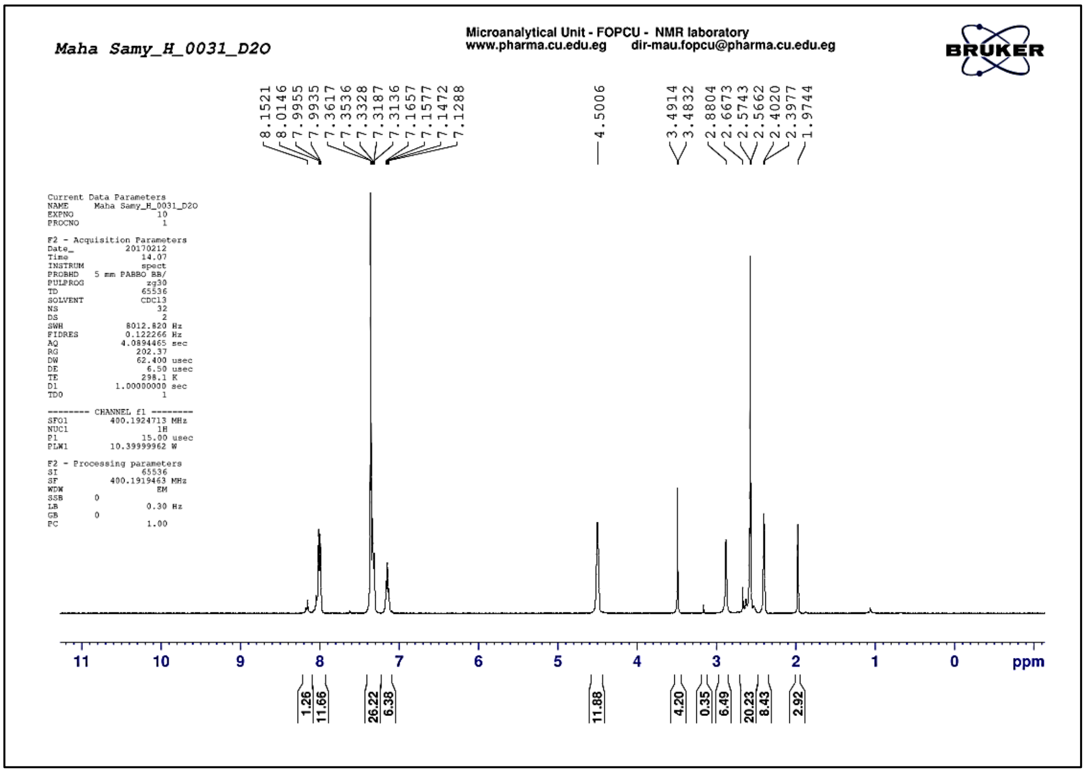

(Fig. 21), *^{1}H-NMR spectrum of 3-methyl-1-phenyl-7-thioxo-7,8-dihydro-1H-pyrazolo [4,3-g]pteridin-5(6H)-one (**12**).DMSO-d_6.D_2O*

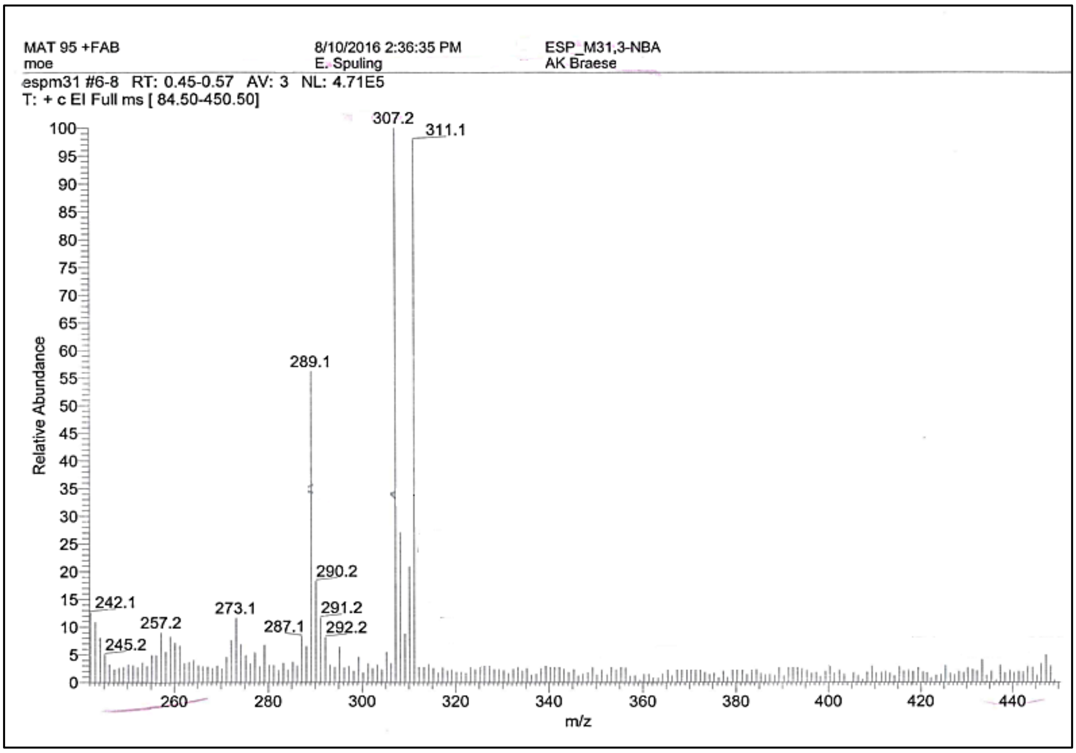

(Fig. 22), *Mass spectrum of 3-methyl-1-phenyl-7-thioxo-7,8-dihydro-1H-pyrazolo [4,3-g]pteridin-5(6H)-one (**12**)*

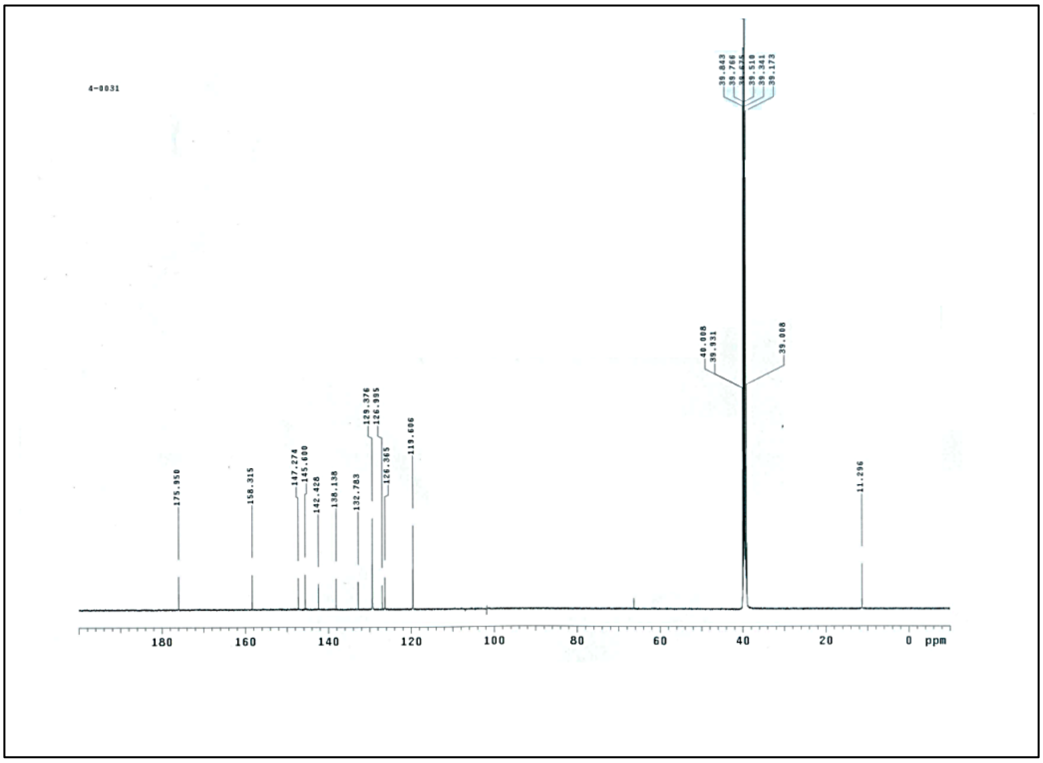

(Fig. 23), *^{13}C-NMR spectrum of 3-methyl-1-phenyl-7-thioxo-7,8-dihydro-1H-pyrazolo [4,3-g]pteridin-5(6H)-one (**12**).DMSO-d$_6$*

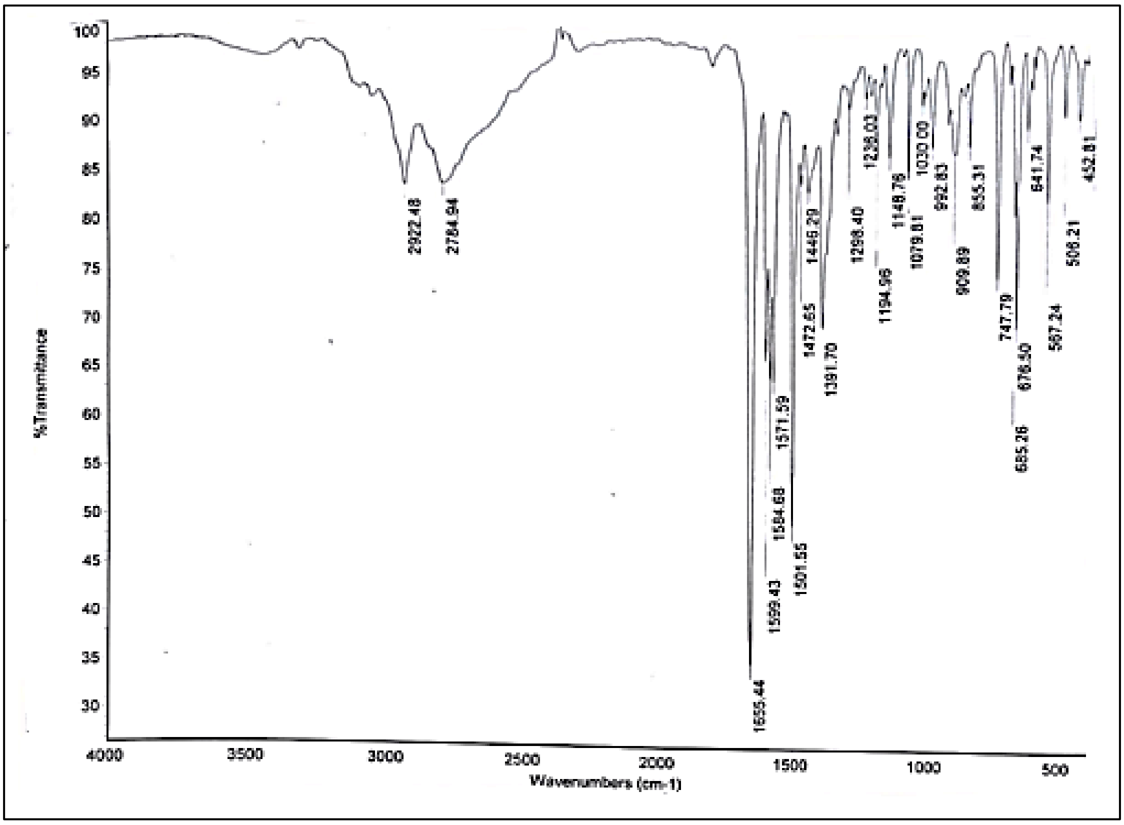

(Fig. 24), *IR spectrum of 6-ethyl-7-(ethylthio)-3-methyl-1-phenyl-1H-pyrazolo[4,3-g] pteridin-5(6H)-one (**13**).*

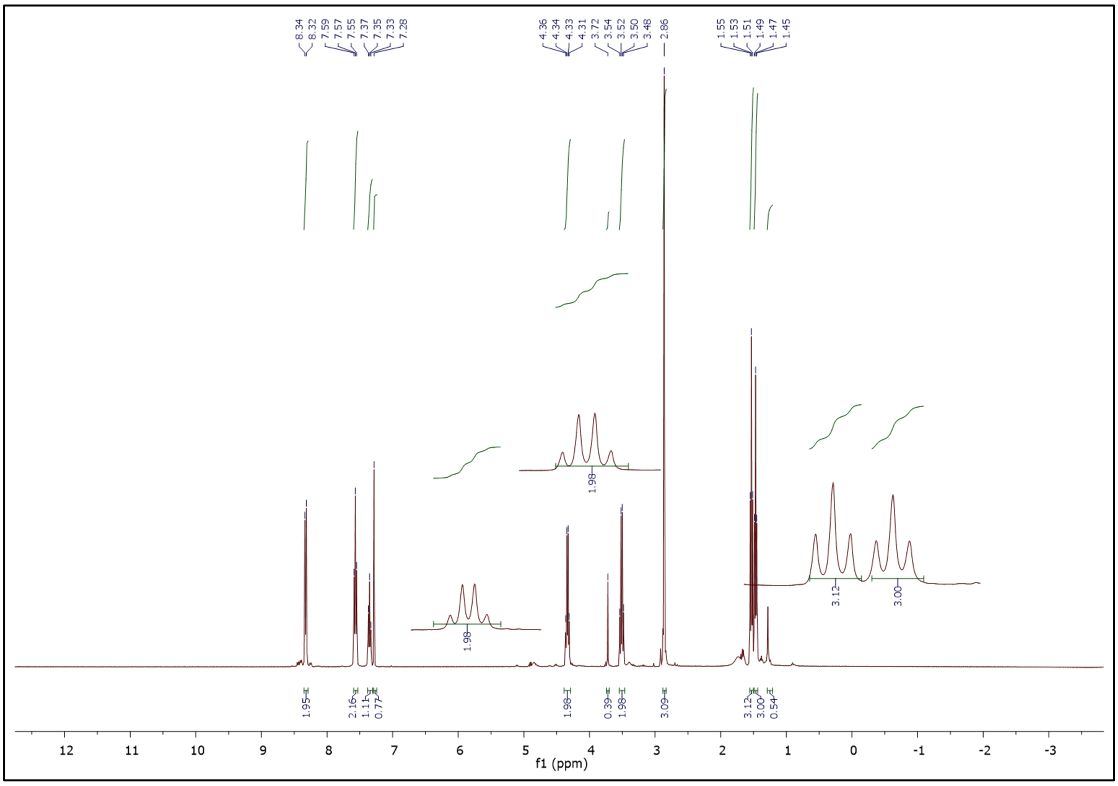

(Fig. 25), *^{1}H-NMR spectrum of 6-ethyl-7-(ethylthio)-3-methyl-1-phenyl-1H-pyrazolo [4,3-g]pteridin-5(6H)-one (**13**) CDCl3.*

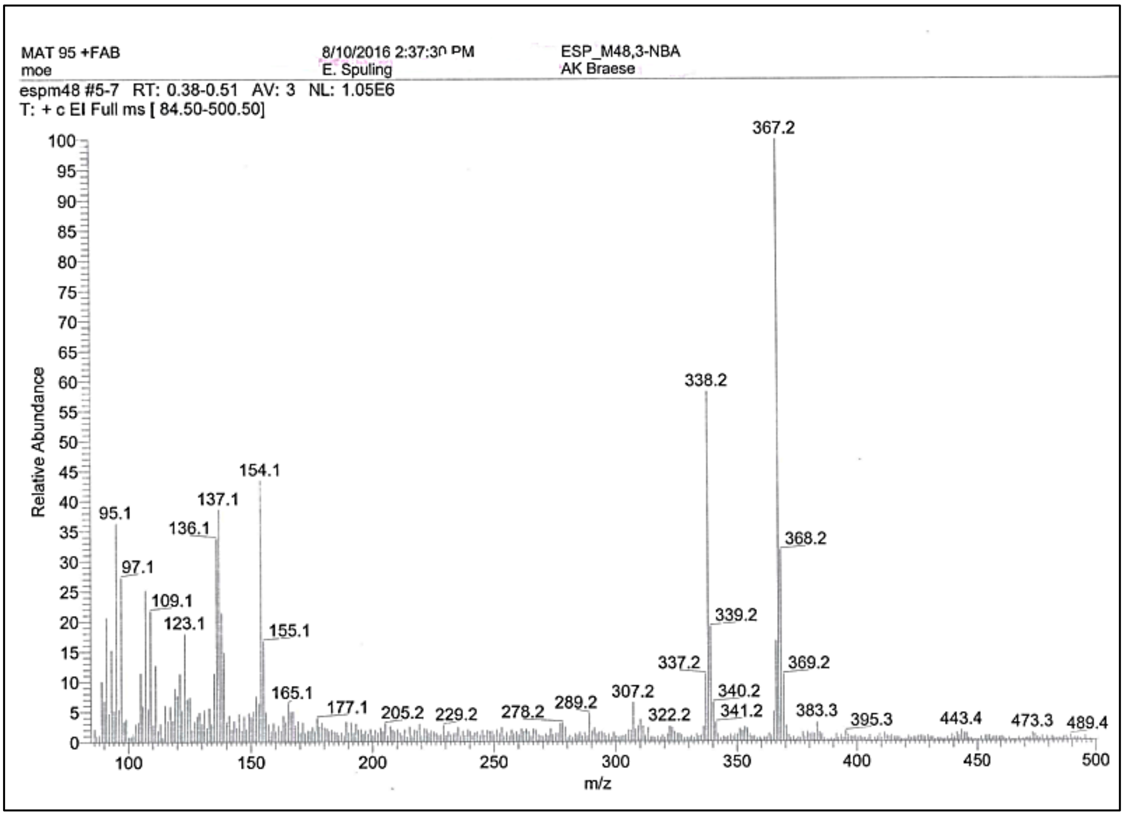

(Fig. 26), *Mass spectrum of 6-ethyl-7-(ethylthio)-3-methyl-1-phenyl-1H-pyrazolo[4,3-g]pteridin-5(6H)-one (**13**).*

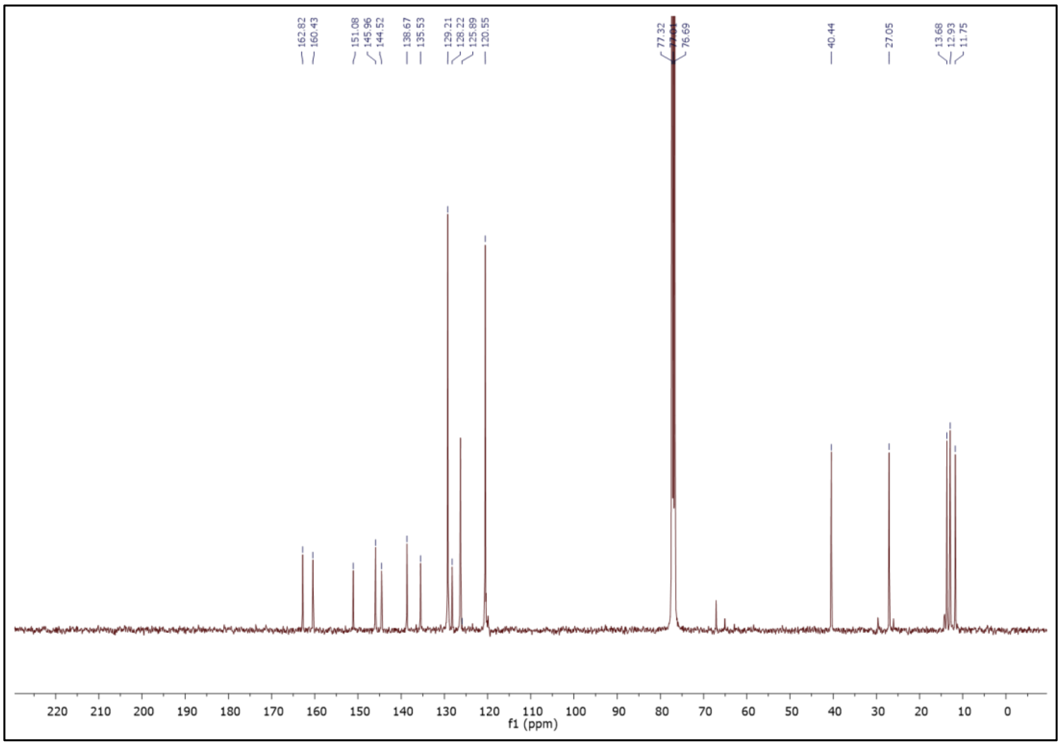

(Fig. 27), *^{13}C-NMR spectrum of 6-ethyl-7-(ethylthio)-3-methyl-1-phenyl-1H-pyrazolo [4,3-g]pteridin-5(6H)-one (**13**). $CDCl_3$*

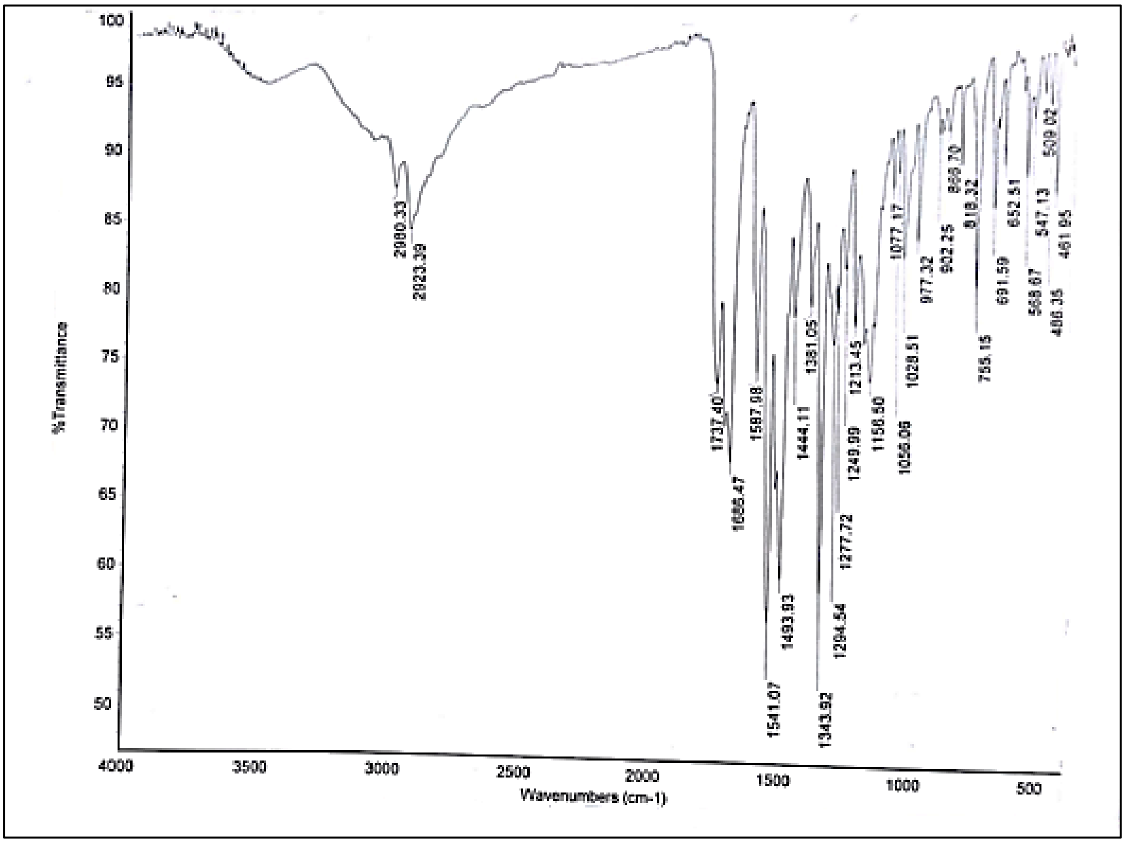

(Fig. 28), *IR spectrum of ethyl 2-(3-methyl-5-oxo-1-phenyl-5,6-dihydro-1H-pyrazolo [4,3-g]pteridin-7-ylthio)acetate.(**14**)*

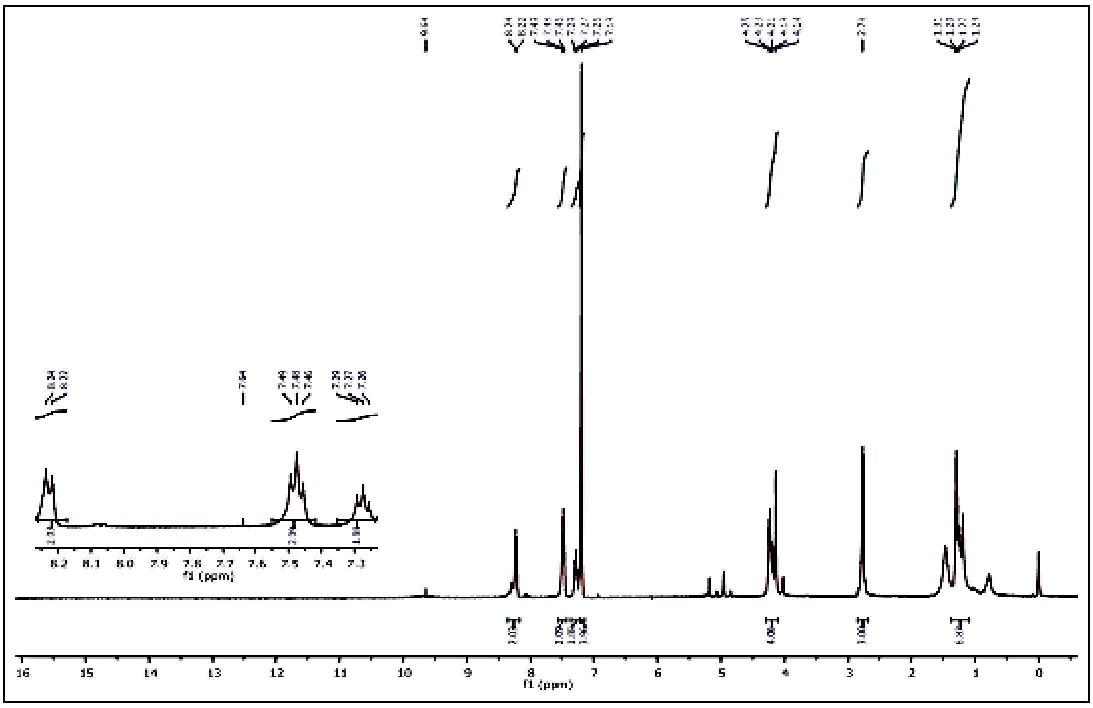

(Fig. 29), *^{1}H-NMR spectrum of ethyl 2-(3-methyl-5-oxo-1-phenyl-5,6-dihydro-1H-pyrazolo[4,3-g]pteridin-7-ylthio)acetate.(**14**).CDCl₃*

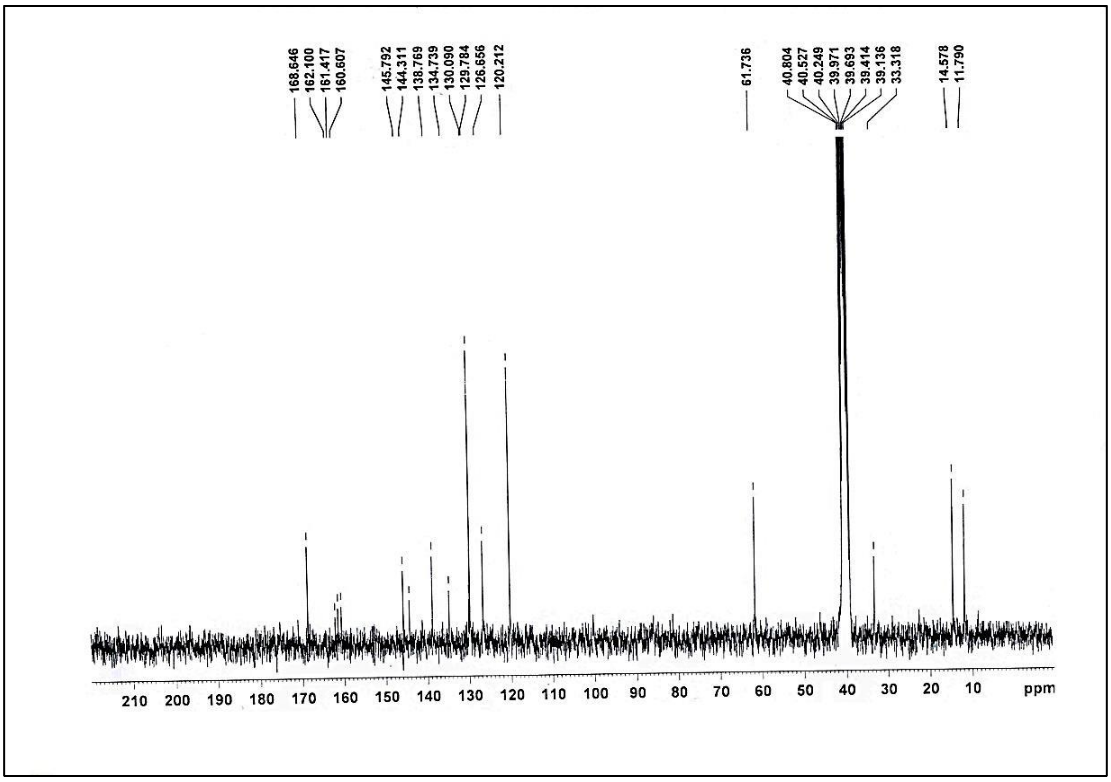

(Fig. 30), *^{13}C-NMR spectrum of ethyl 2-(3-methyl-5-oxo-1-phenyl-5,6-dihydro-1H-pyrazolo[4,3-g]pteridin-7-ylthio)acetate.(**14**).CDCl₃*

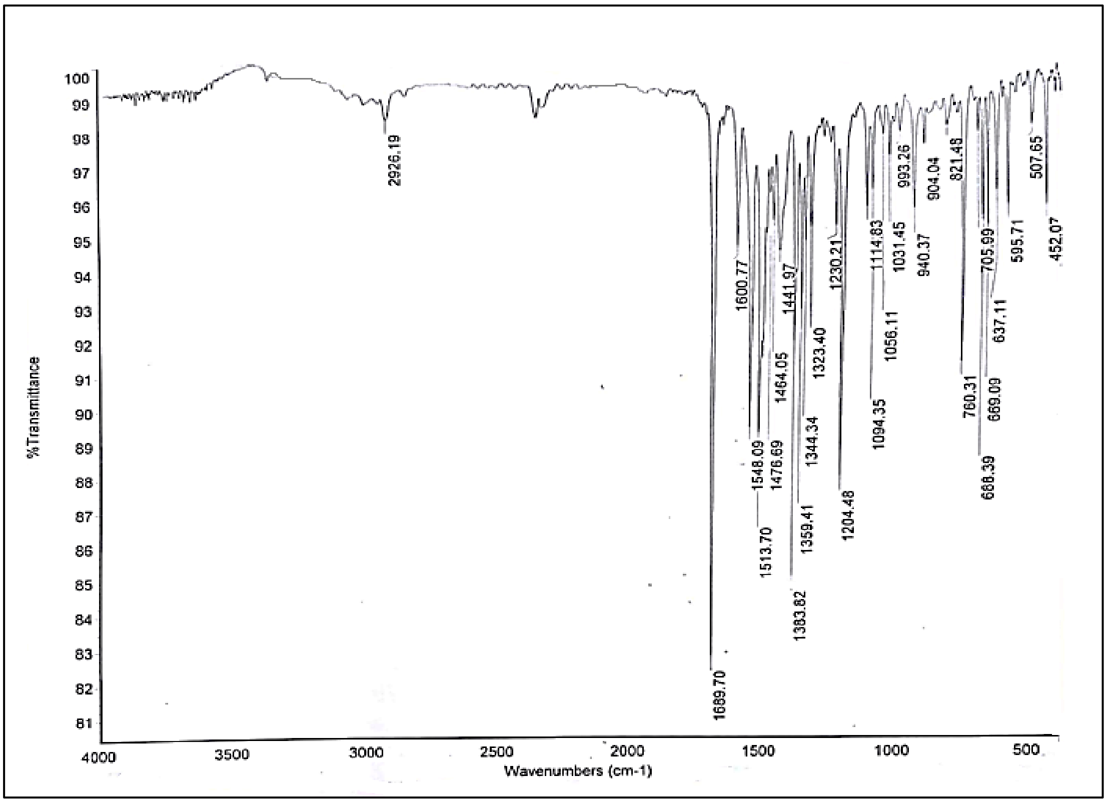

(Fig. 31), IR spectrum of 5-acetyl-3,6-dimethyl-1-phenyl-1H-pyrazolo[3,4-b]pyrazine (15)

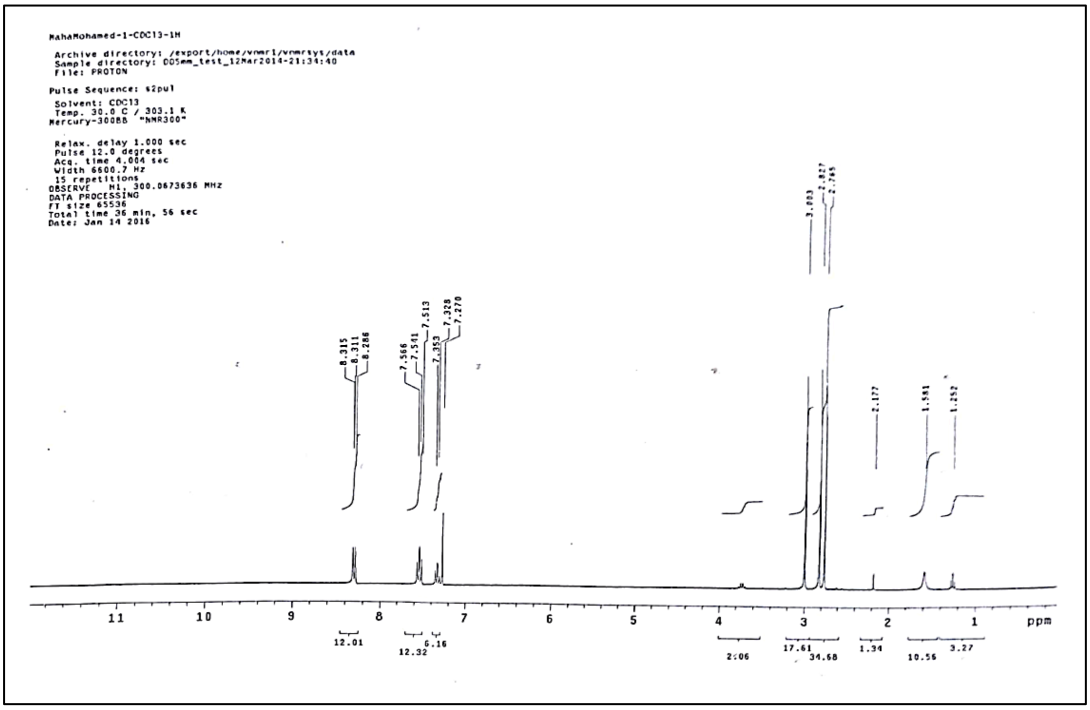

*(Fig. 32), ^{1}H-NMR spectrum of 5-acetyl-3,6-dimethyl-1-phenyl-1H-pyrazolo[3,4-b] pyrazine (**15**).$CDCl_3$.*

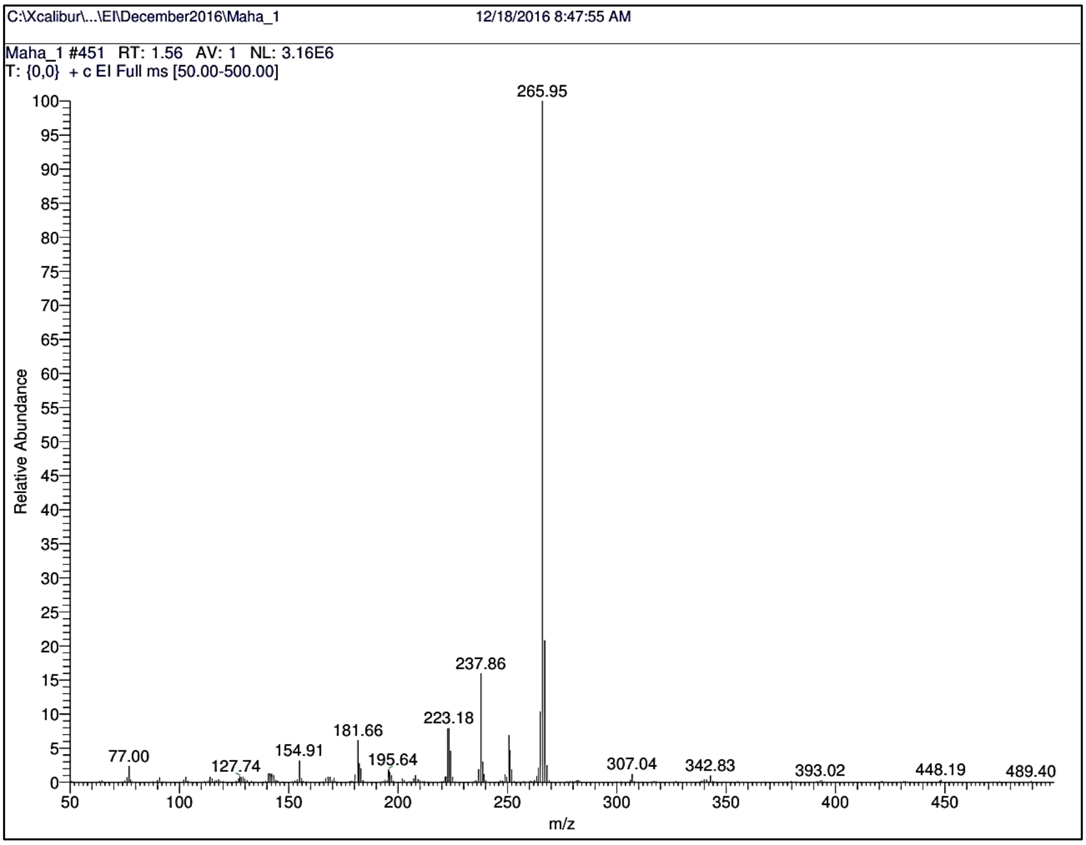

(Fig. 33), *Mass spectrum of 5-acetyl-3,6-dimethyl-1-phenyl-1H-pyrazolo[3,4-b] pyrazine (**15**).*

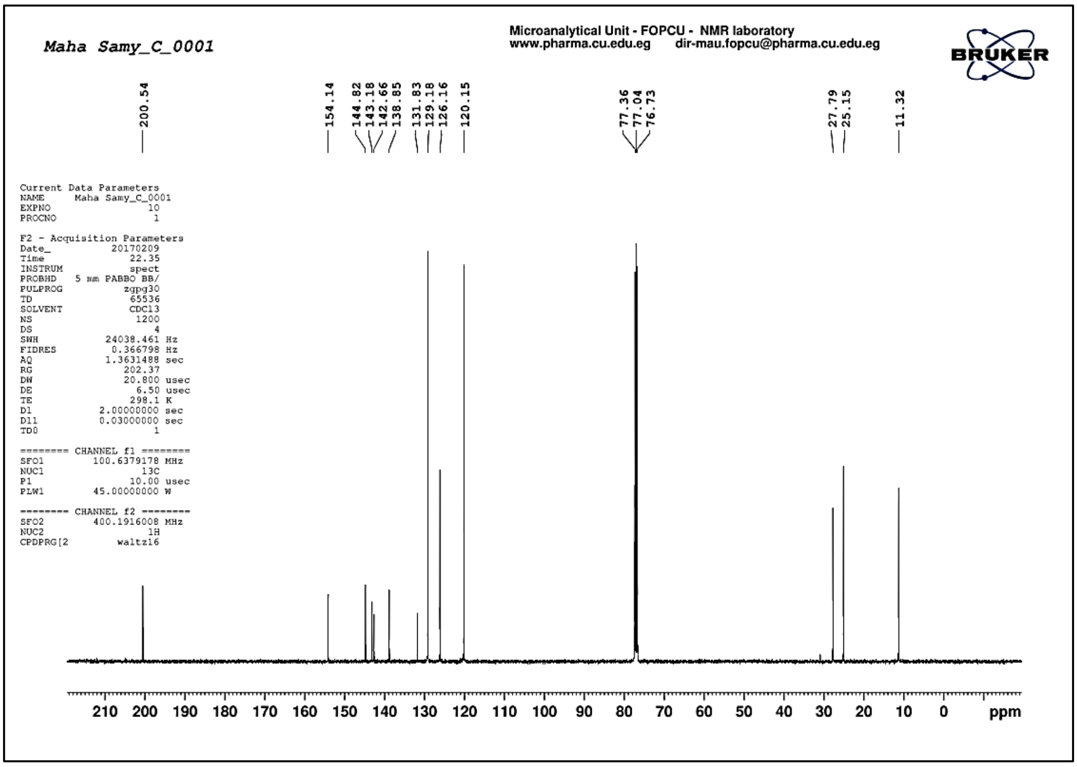

(Fig. 34), *^{13}C-NMR spectrum of 5-acetyl-3,6-dimethyl-1-phenyl-1H-pyrazolo[3,4-b] pyrazine (**15**).$CDCl_3$.*

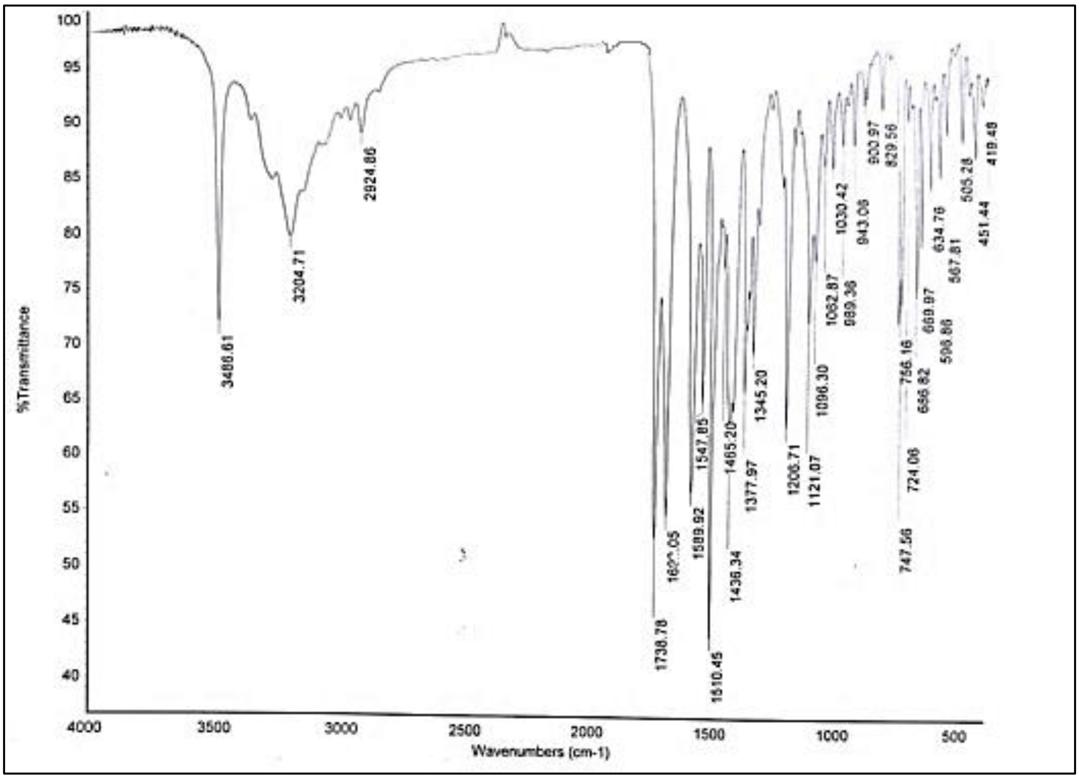

(Fig. 35), *IR spectrum of (3,6-dimethyl-1-phenyl-5-acetyl-1H-pyrazolo[3,4- b] pyrazine)semicarbazone (**16**).*

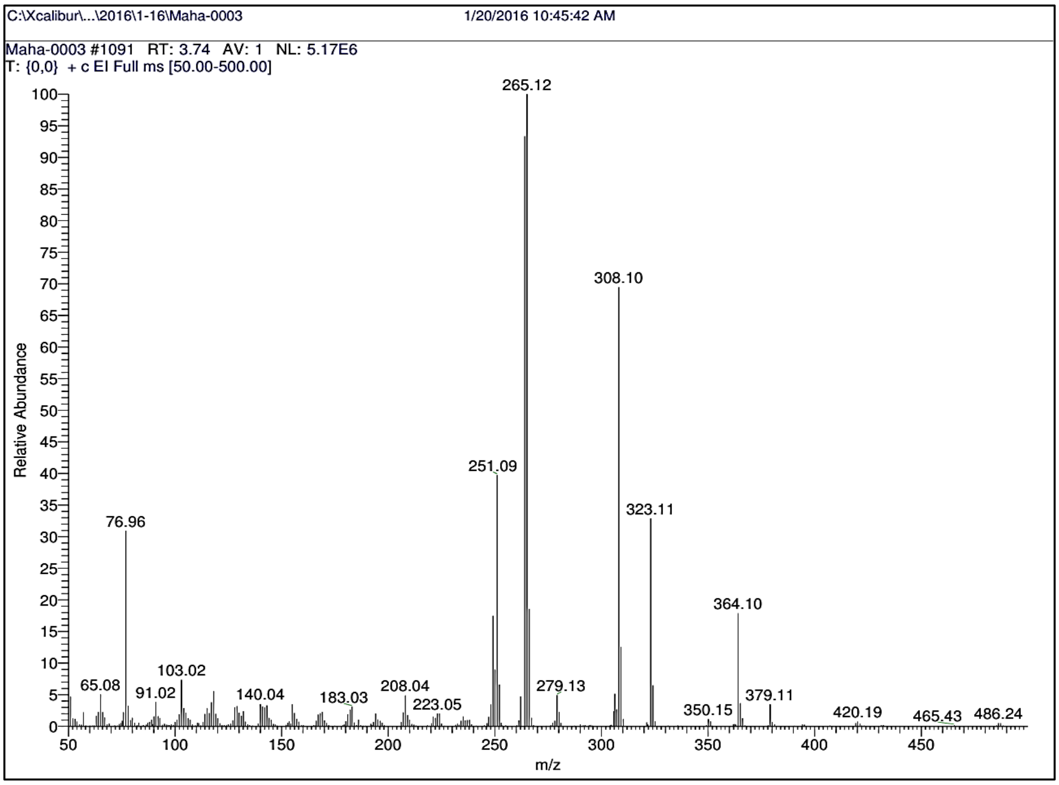

(Fig. 36), *Mass spectrum of (3,6-dimethyl-1-phenyl-5-acetyl-1H-pyrazolo[3,4- b] pyrazine)semicarbazone (**16**).*

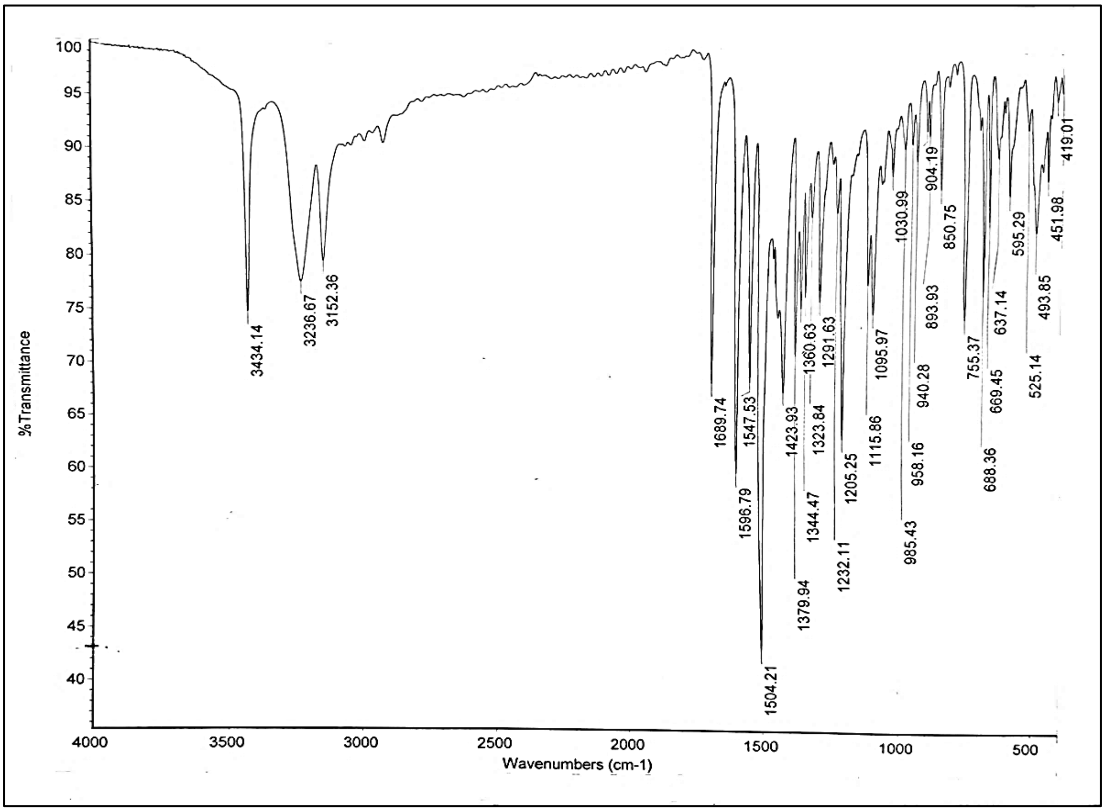

(Fig. 37), *IR spectrum of (3,6-dimethyl-1-phenyl-5-acetyl-1H-pyrazolo[3,4-b] pyrazine)thiosemicarbazone (**17**).*

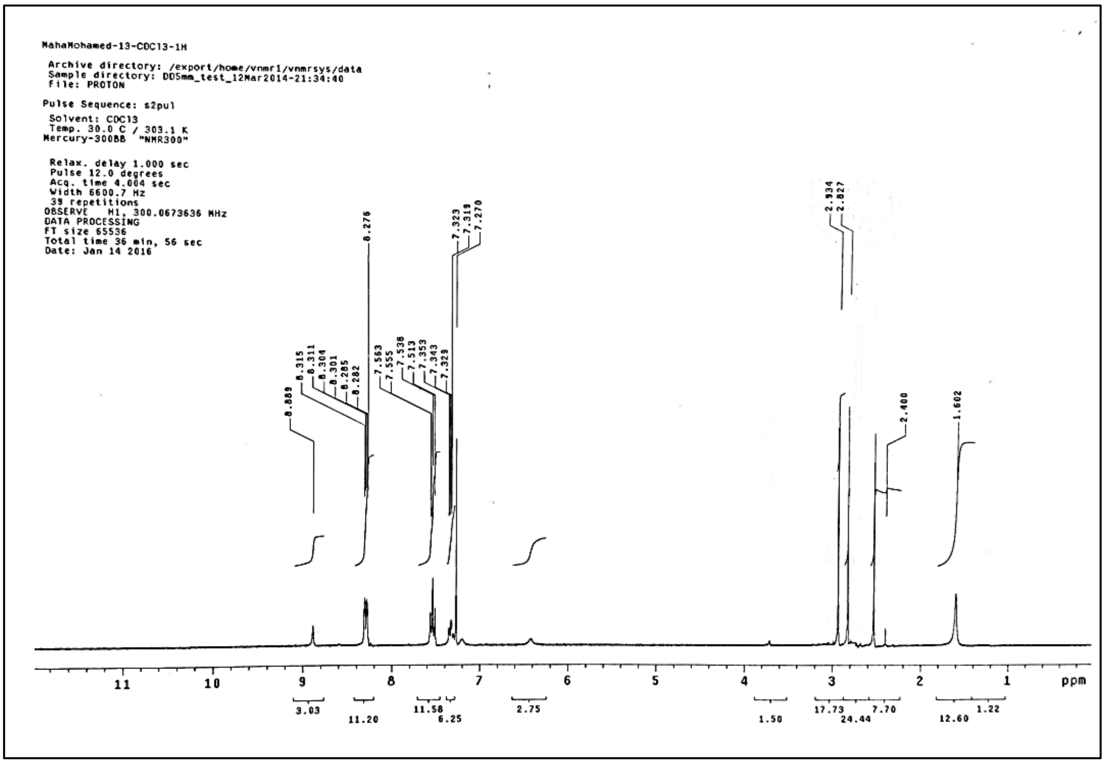

(Fig. 38), *^{1}H-NMR spectrum of (3,6-dimethyl-1-phenyl-5-acetyl-1H-pyrazolo[3,4b]pyrazine)thiosemicarbazone (**17**).CDCl*$_3$

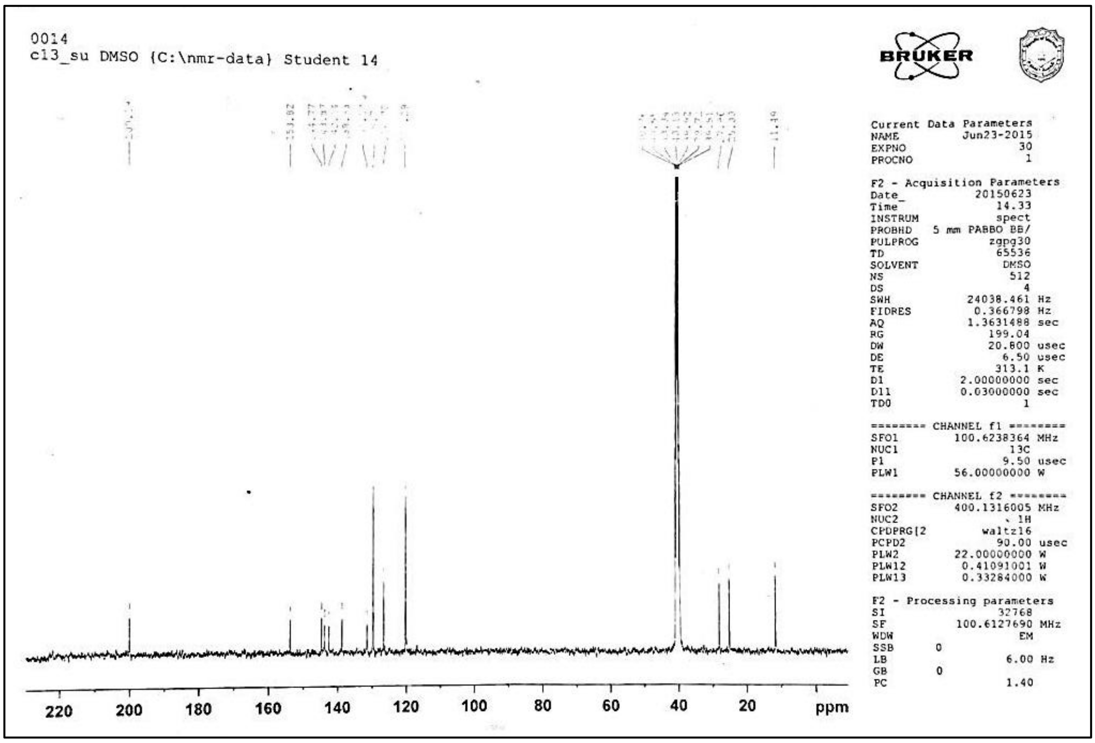

(Fig. 39), *^{13}C-NMR spectrum of (3,6-dimethyl-1-phenyl-5-acetyl-1H-pyrazolo[3,4b]pyrazine)thiosemicarbazone (**17**). $CDCl_3$*

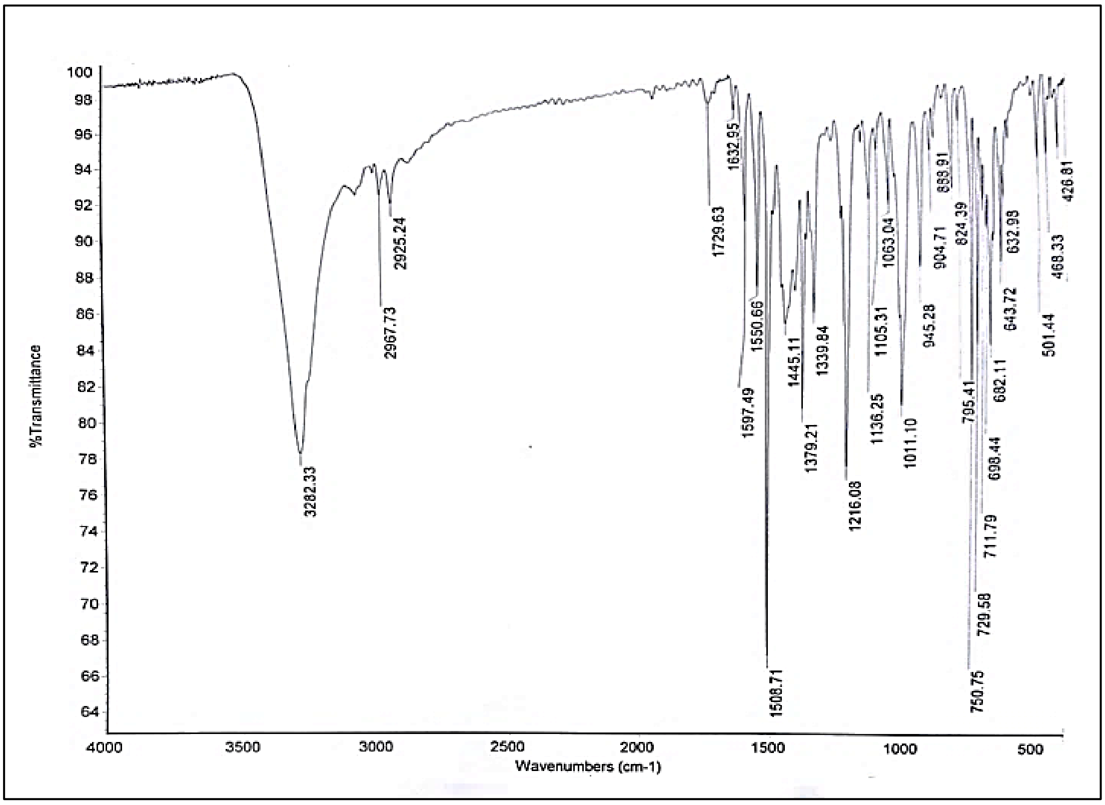

(Fig. 40), *IR spectrum of 3,6-dimethyl-1-phenyl-1H-pyrazolo[3,4-b]pyrazin-5-ethanone oxime (**18**)*

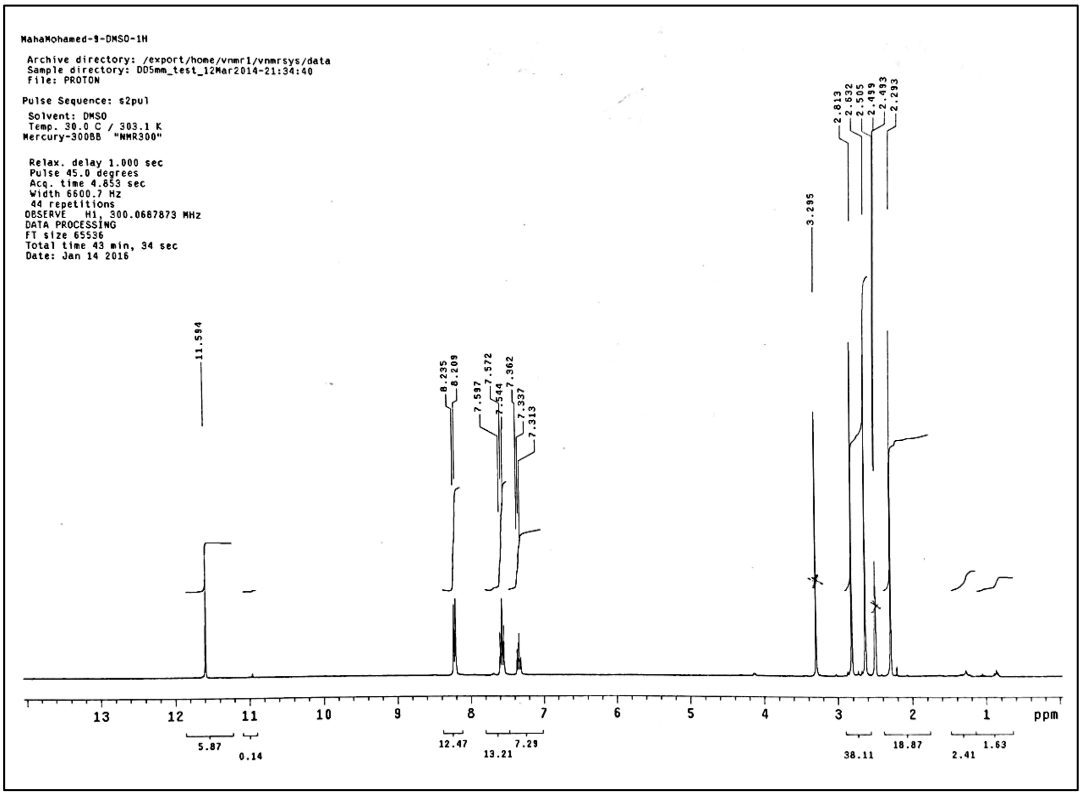

(Fig. 41), *¹H-NMR spectrum of 3,6-dimethyl-1-phenyl-1H-pyrazolo[3,4-b]pyrazin-5-ethanone oxime (**18**) DMSO-d$_6$*

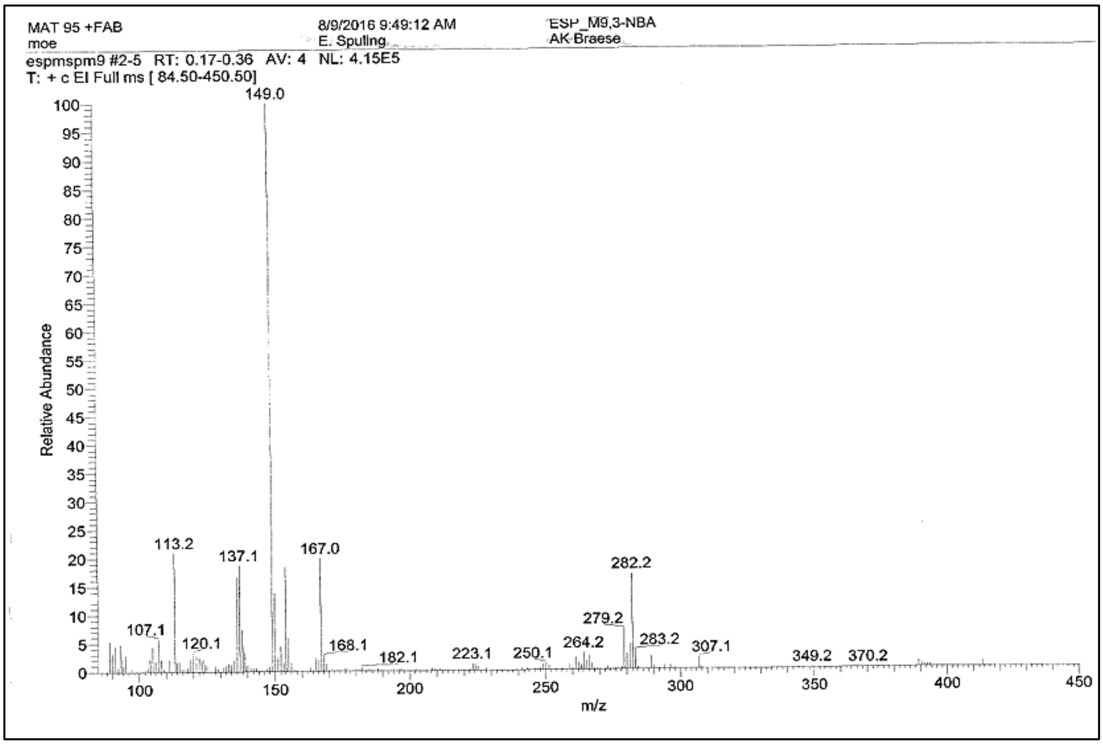

(Fig. 42), *Mass spectrum of 3,6-dimethyl-1-phenyl-1H-pyrazolo[3,4-b]pyrazin-5-ethanone oxime (**18**)*

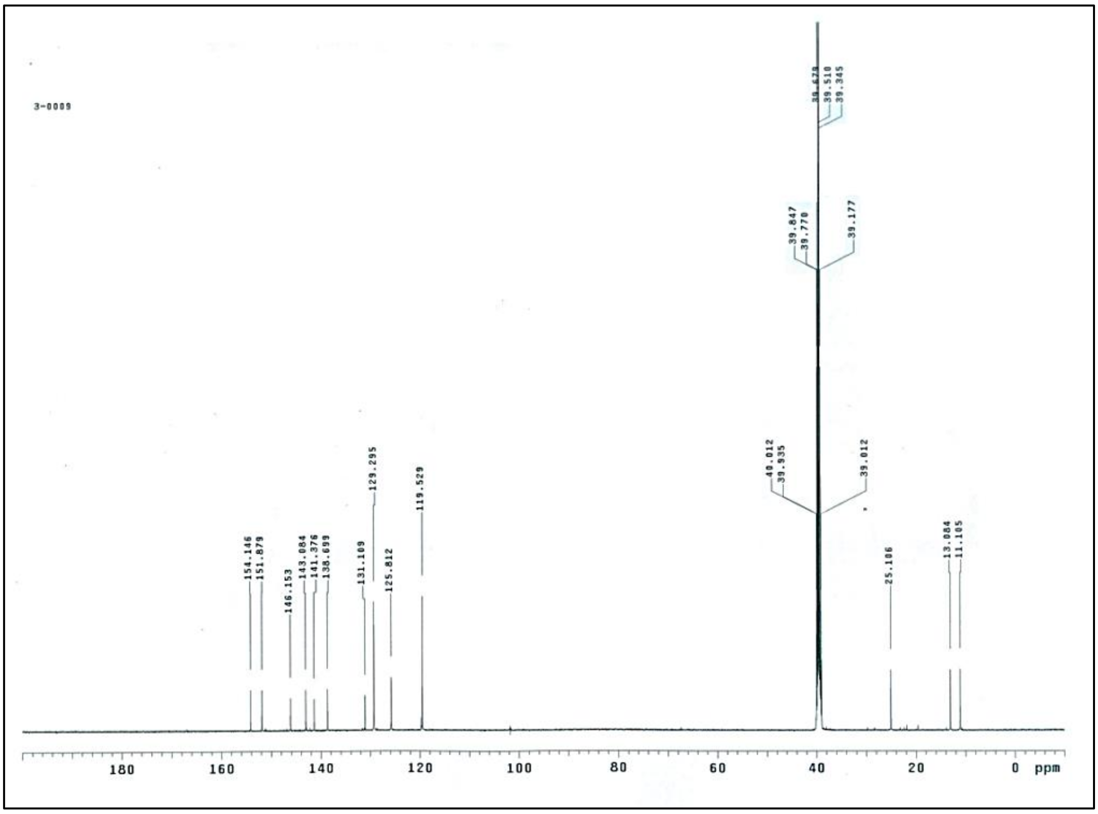

(Fig. 43), *^{13}C-NMR spectrum of 3,6-dimethyl-1-phenyl-1H-pyrazolo[3,4-b]pyrazin-5-ethanone oxime (**18**).DMSO-d_6*

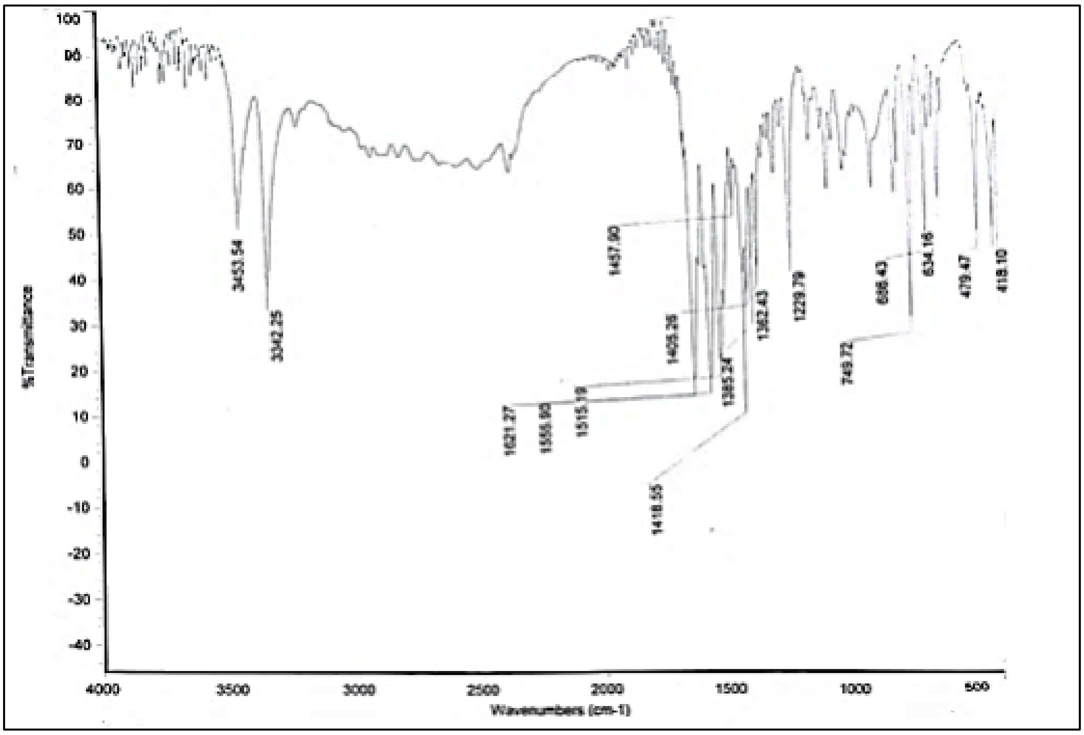

(Fig. 44)*, IR spectrum of (5-acetyl-3,6-dimethyl-1-phenyl-1H-pyrazolo[3,4-b] pyrazine)hydrazone (**19**).$CDCl_3$*

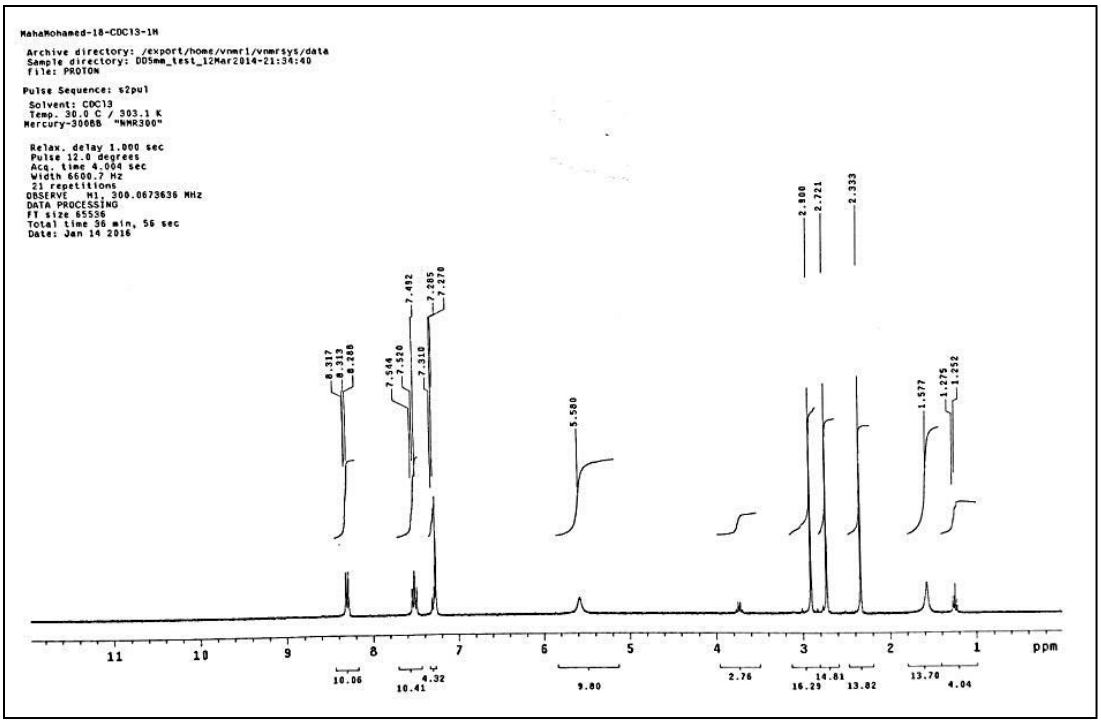

***(Fig. 45)**, ^{1}H-NMR spectrum of (5-acetyl-3,6-dimethyl-1-phenyl-1H-pyrazolo[3,4-b] pyrazine)hydrazone (**19**).CDCl$_3$*

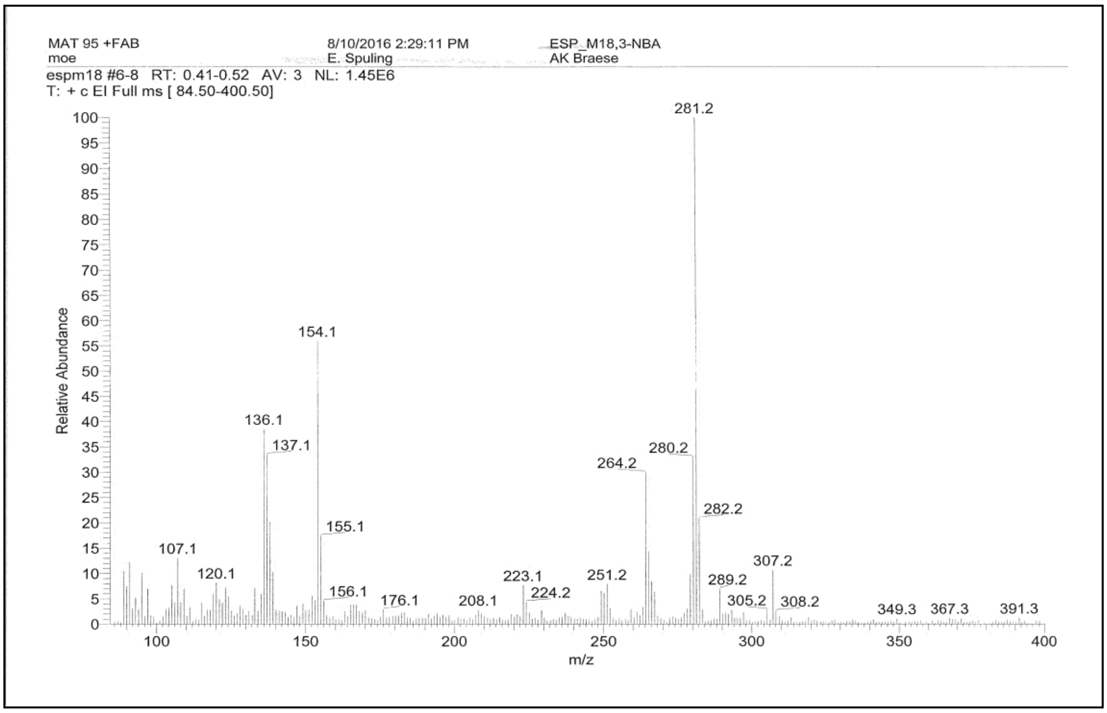

***(Fig. 46)**, Mass spectrum of (5-acetyl-3,6-dimethyl-1-phenyl-1H-pyrazolo[3,4-b] pyrazine)hydrazone (**19**).*

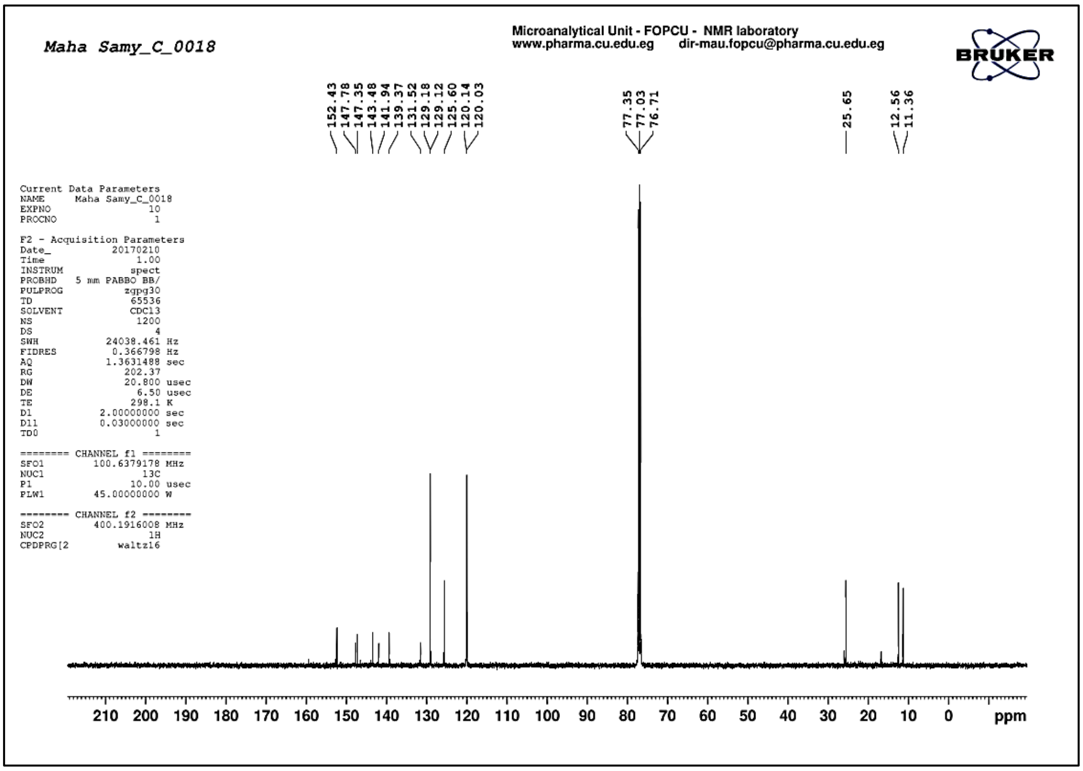

(Fig. 47), *^{13}C-NMR spectrum of (5-acetyl-3,6-dimethyl-1-phenyl-1H-pyrazolo[3,4-b] pyrazine)hydrazone (**19**).$CDCl_3$*

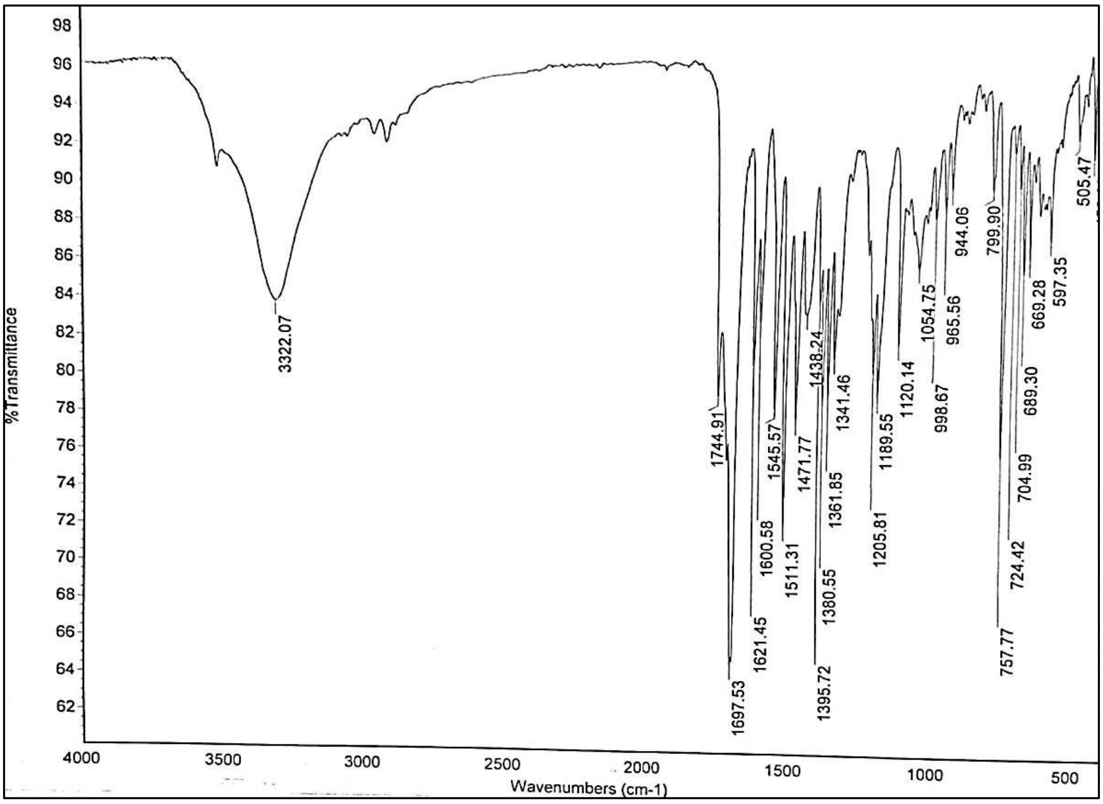

(Fig. 48), *^{1}H-NMR spectrum of 5-(3-hydroxy-2,3-dihydroindol-2-on-3-ylacetyl)-3,6-dimethyl-1-phenyl-1H-pyrazolo[3,4-b]pyrazine (**20**).*

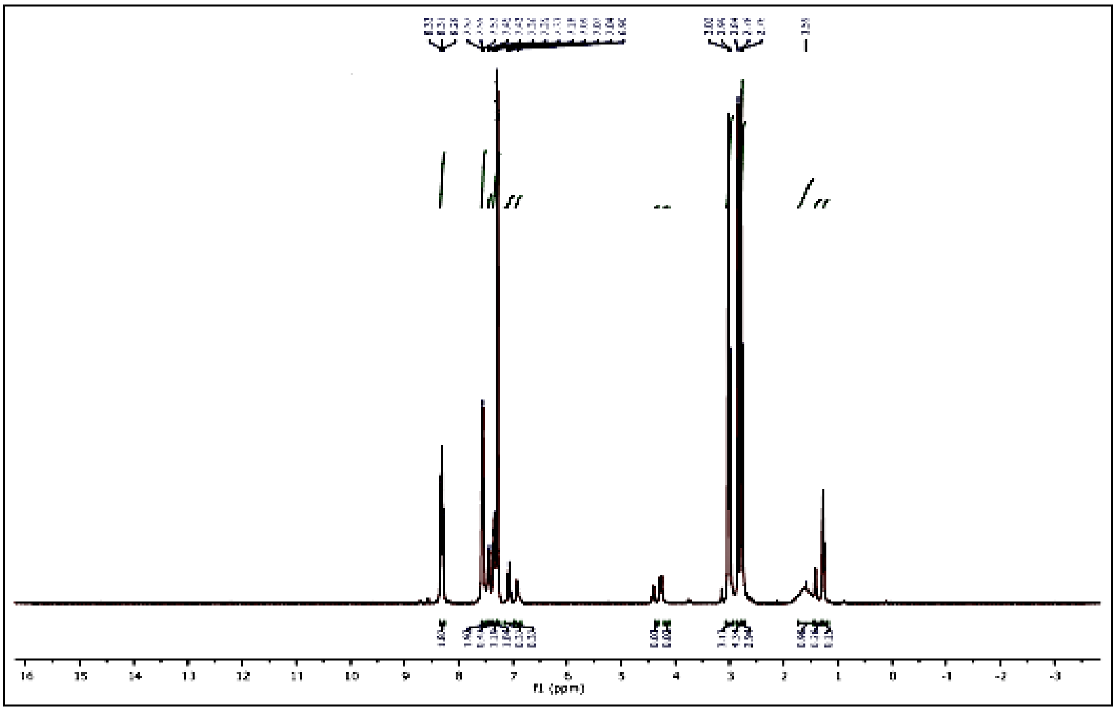

(Fig. 49), *^{1}H-NMR spectrum of 5-(3-hydroxy-2,3-dihydroindol-2-on-3-ylacetyl)-3,6-dimethyl-1-phenyl-1H-pyrazolo[3,4-b]pyrazine (**20**).CDCl$_3$*

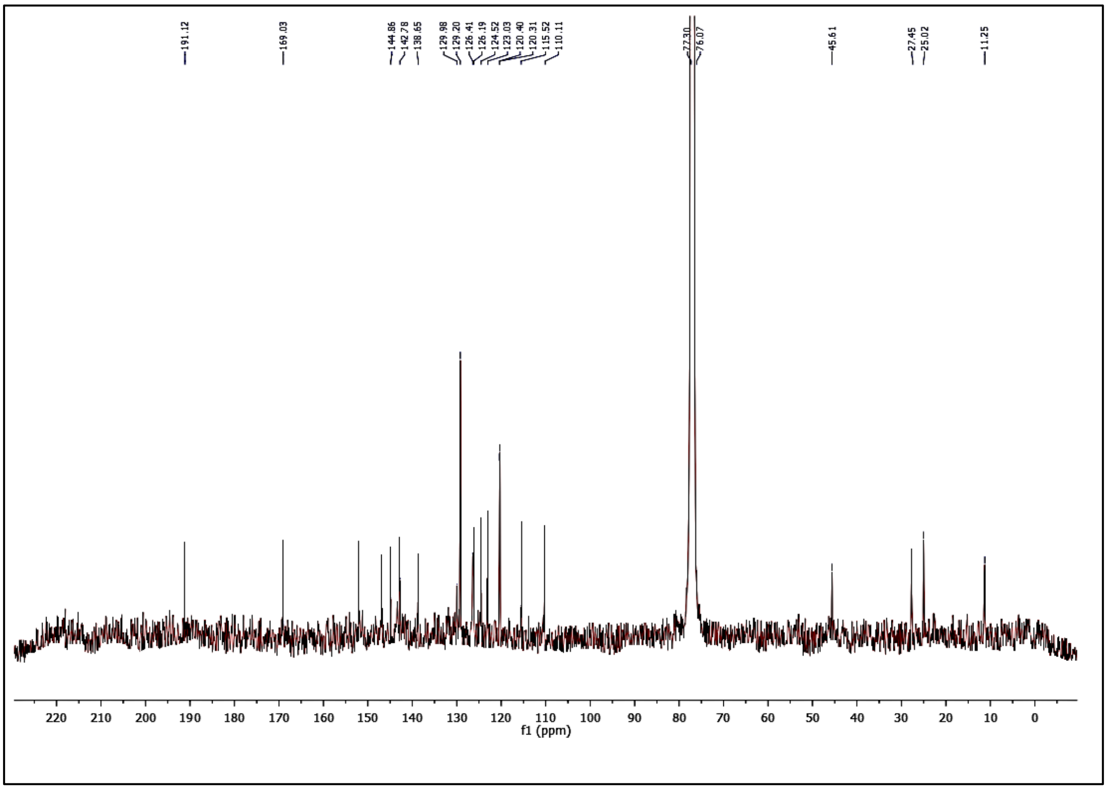

(Fig. 50), *^{13}C-NMR spectrum of 5-(3-hydroxy-2,3-dihydroindol-2-on-3-ylacetyl)-3,6-dimethyl-1-phenyl-1H-pyrazolo[3,4-b]pyrazine (**20**).CDCl$_3$*

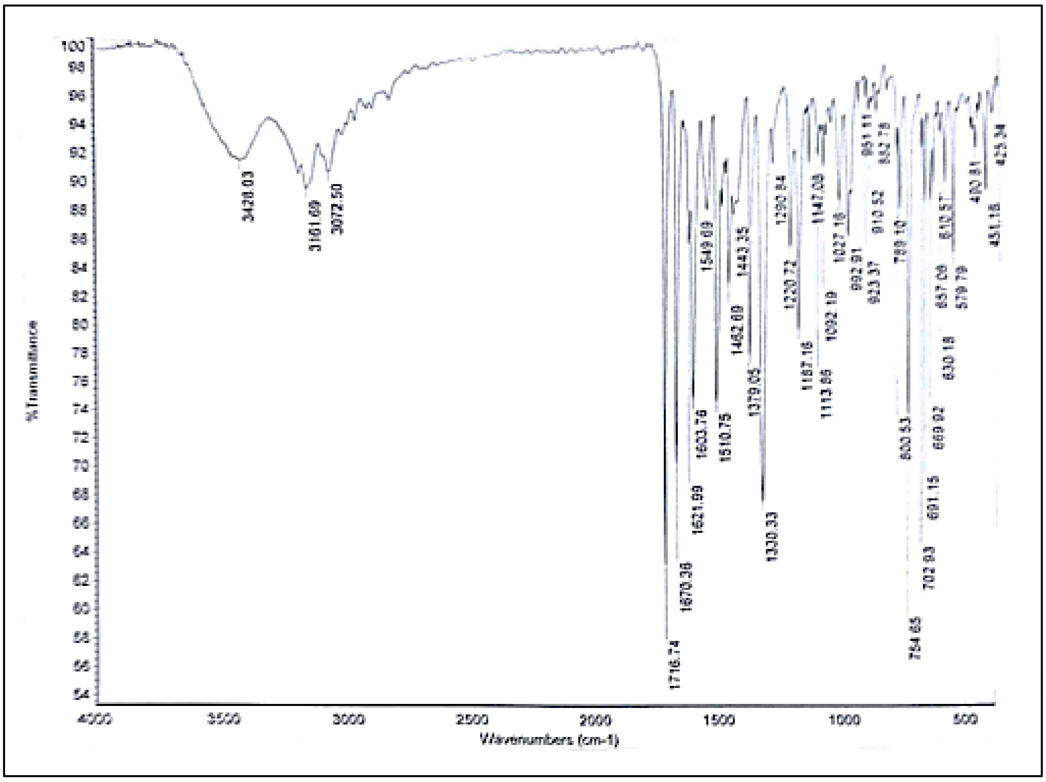

(Fig. 51)*, IR spectrum of 2,3-dihydroindol-2-on-3-ylideneacetyl-3,6-dimethyl-1-phenyl-1H-pyrazolo[3,4-b]pyrazine (***21***)*

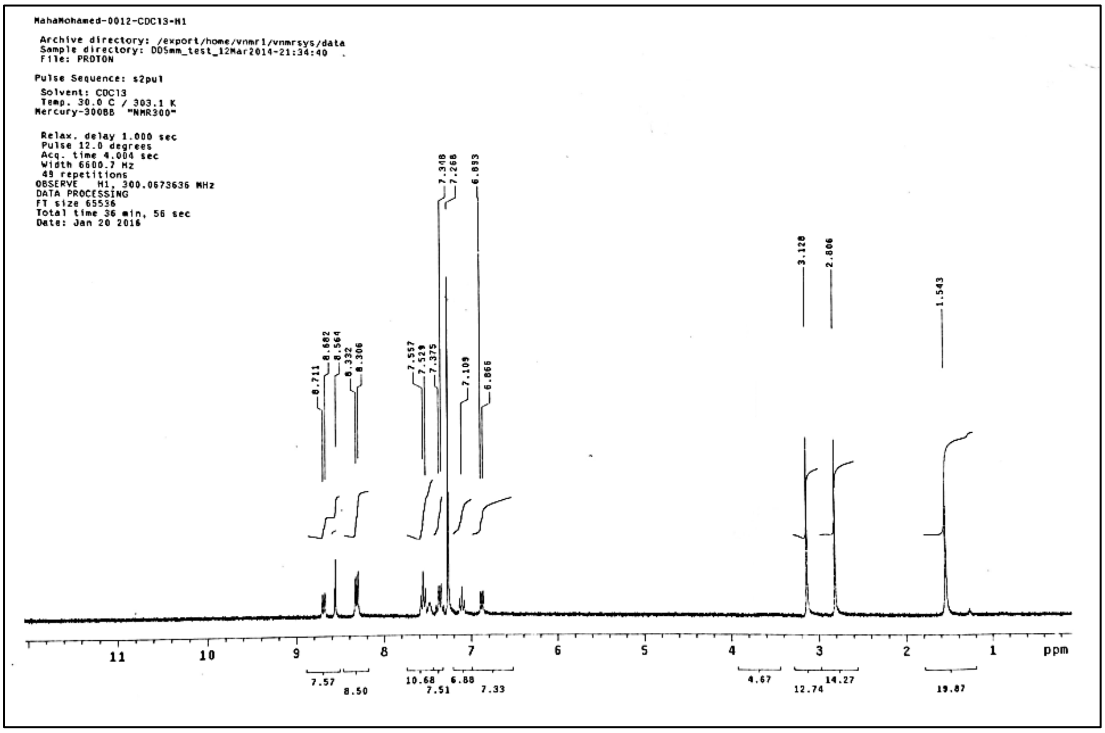

(Fig. 52)*, ^{1}H-NMR spectrum of 2,3-dihydroindol-2-on-3-ylideneacetyl-3,6-dimethyl-1-phenyl-1H-pyrazolo[3,4-b] pyrazine (***21***).CDCl*$_3$

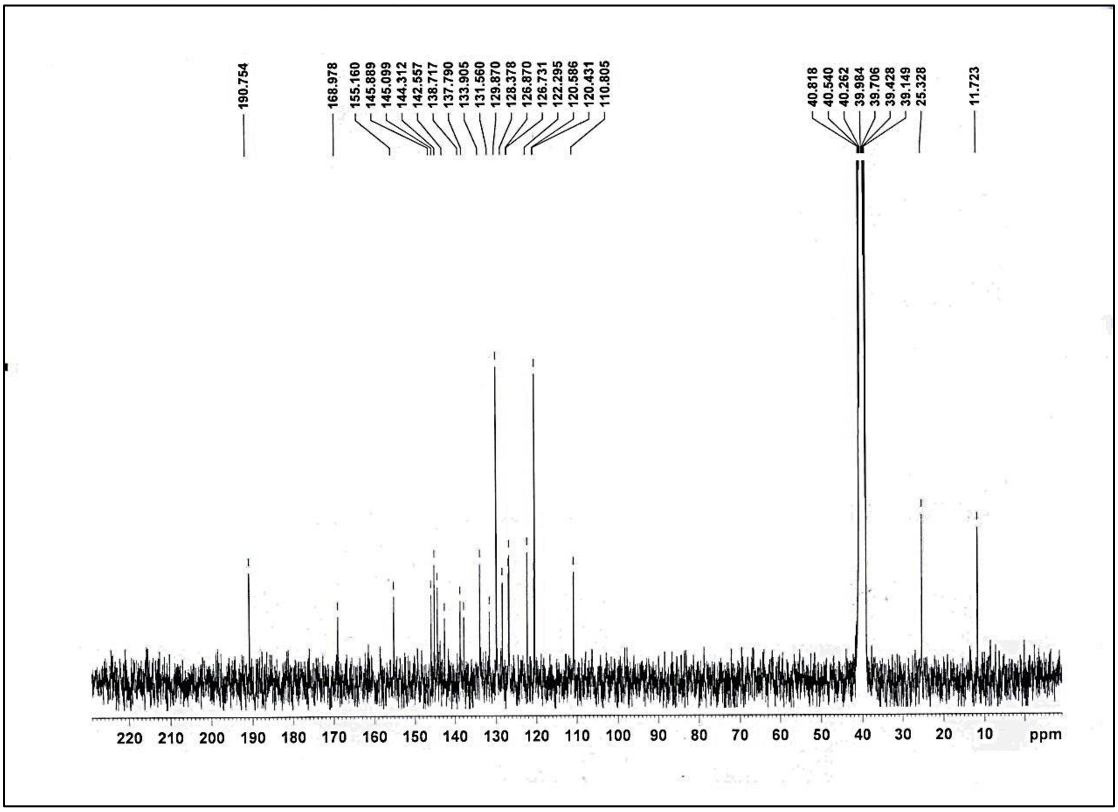

(Fig. 53), *^{13}C-NMR spectrum of 2,3-dihydroindol-2-on-3-ylideneacetyl-3,6-dimethyl-1-phenyl-1H-pyrazolo[3,4-b] pyrazine (**21**).DMSO-d_6*

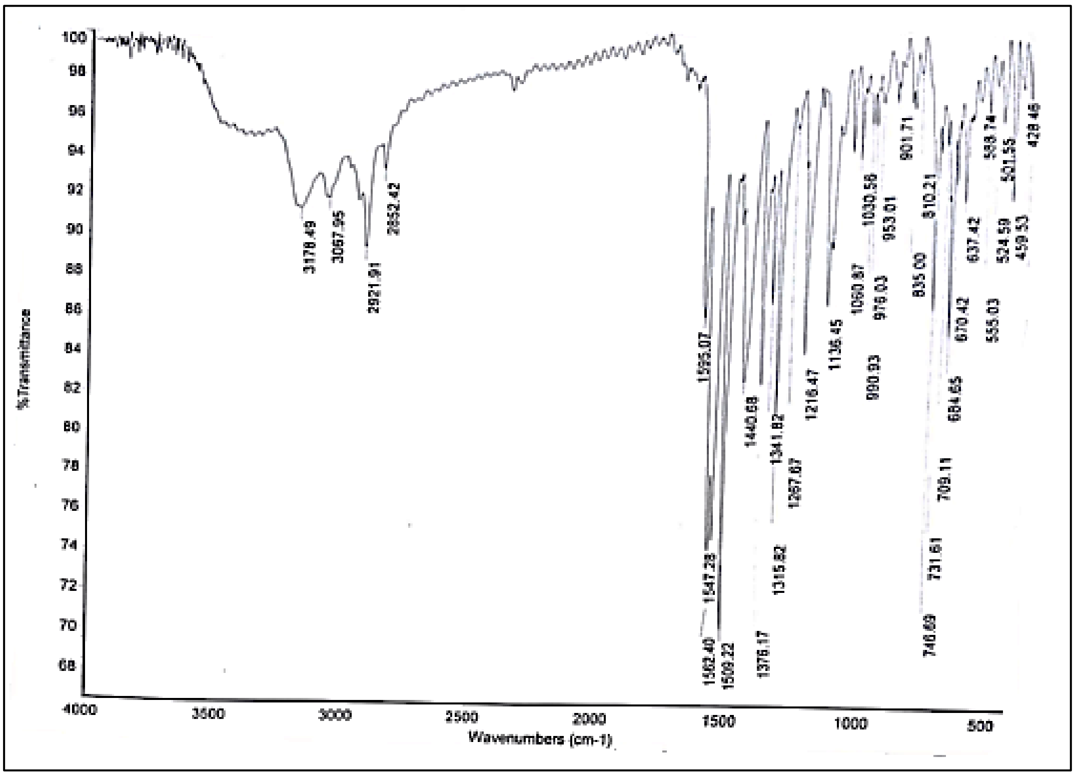

(Fig. 54), *IR spectrum of N-(4-methyl-2,3-dihydrothiazol-2-ylidene)-(5-acetyl-3,6-dimethyl-1-phenyl-1H-pyrazolo[3,4-b]pyrazine)hydrazine (**22**).*

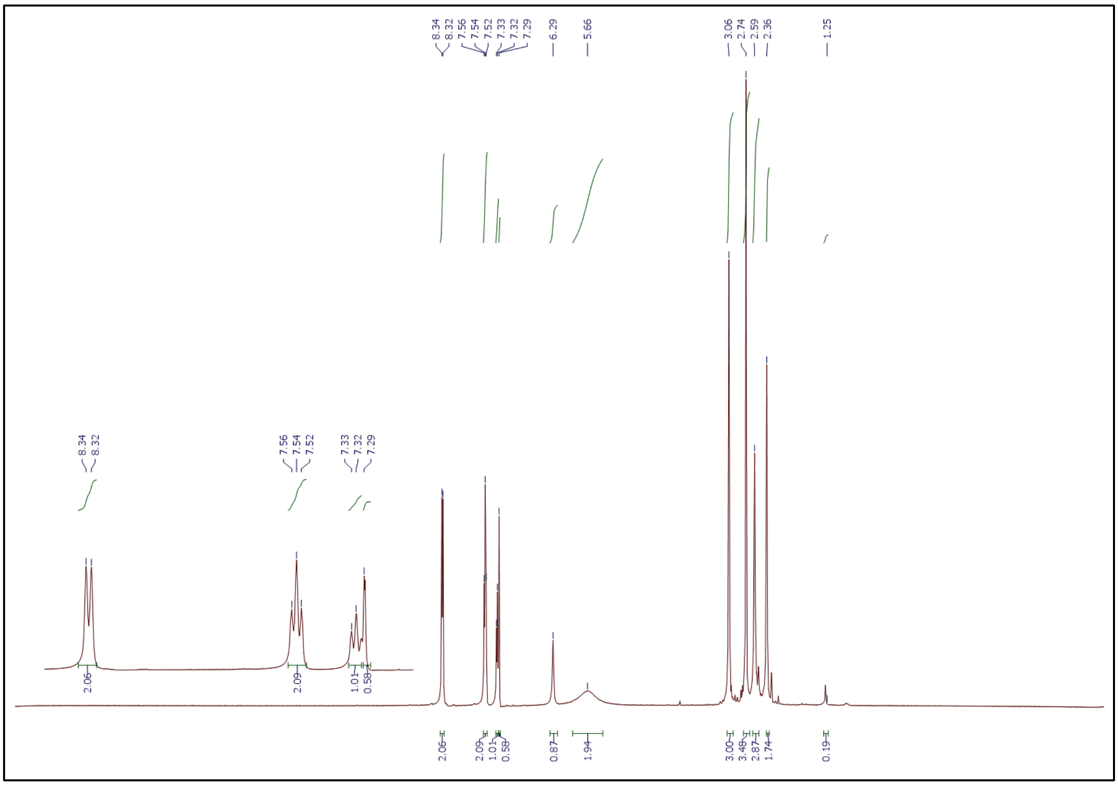

(Fig. 55), *^{1}H-NMR spectrum of N-(4-methyl-2,3-dihydrothiazol-2-ylidene)-(5-acetyl-3,6-dimethyl-1-phenyl-1H-pyrazolo[3,4-b]pyrazine)hydrazine (**22**).CDCl$_3$.*

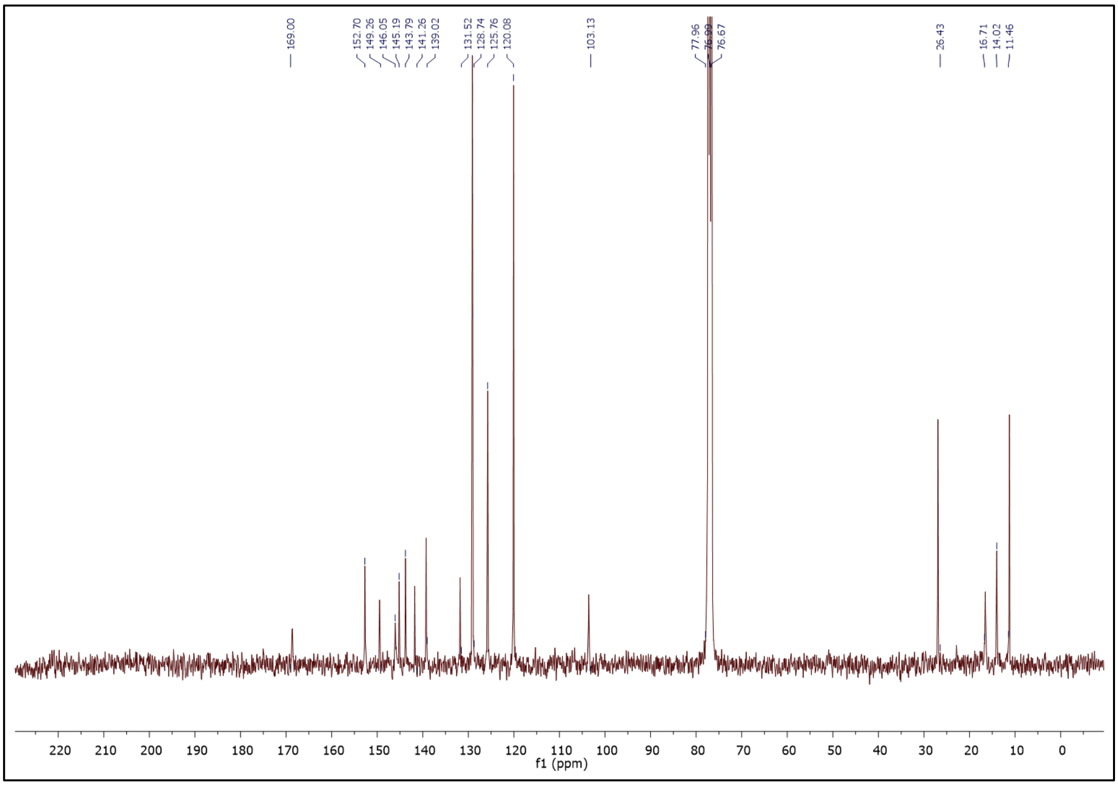

(Fig. 56), *^{13}C-NMR spectrum of N-(4-methyl-2,3-dihydrothiazol-2-ylidene)-(5-acetyl-3,6-dimethyl-1-phenyl-1H-pyrazolo[3,4-b]pyrazine)hydrazine (**22**).CDCl$_3$.*

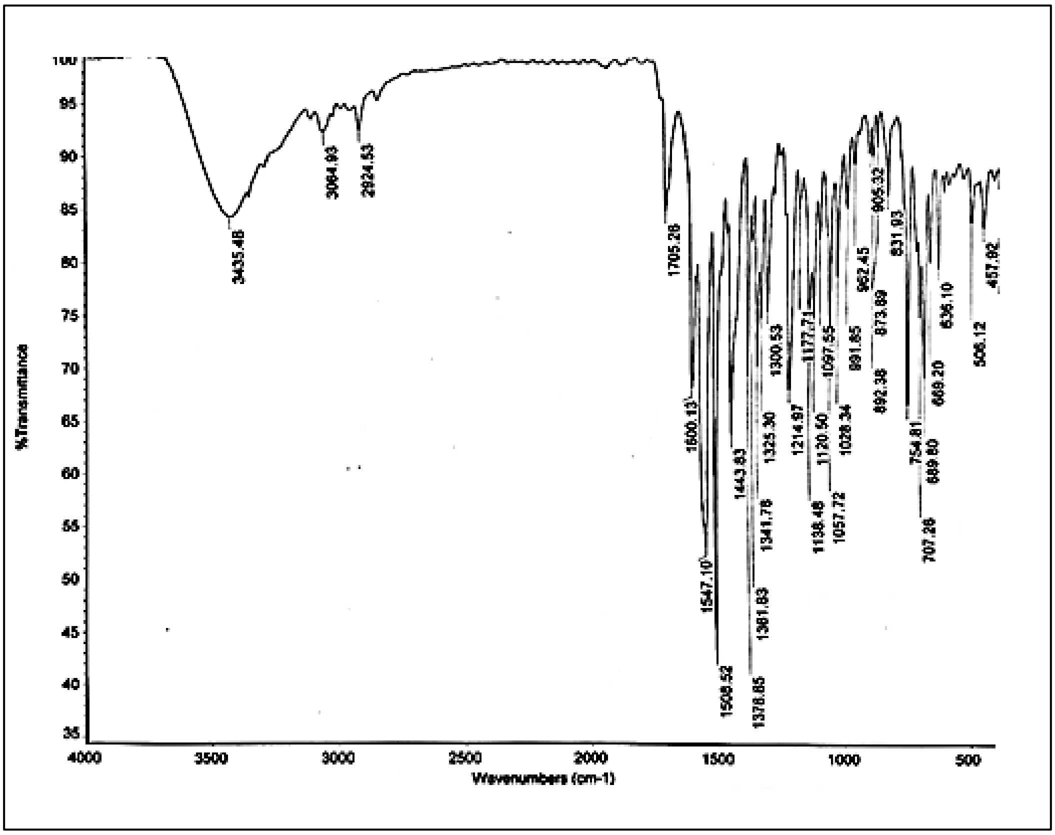

***(Fig. 57),** IR spectrum of N-(4-phenyl-2,3-dihydrothiazol-2-ylidene)-(5-acetyl-3,6-dimethyl-1-phenyl-1H-pyrazolo[3,4-b]pyrazine)hydrazine (**23**).*

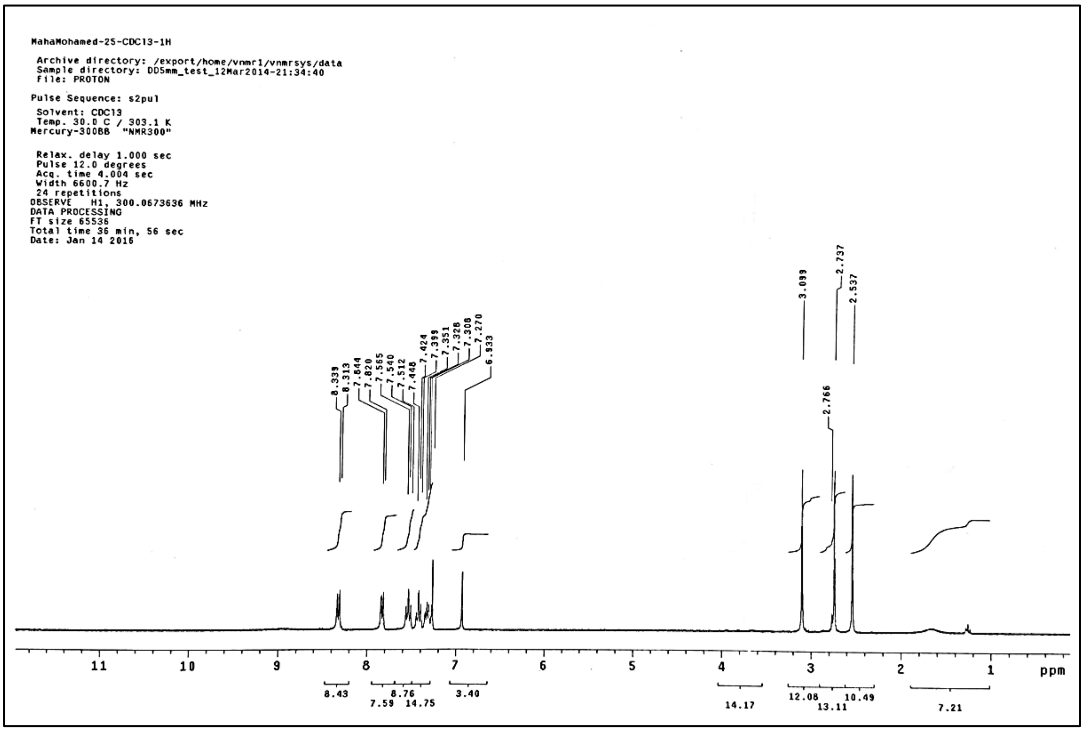

***(Fig. 58),** ¹H-NMR spectrum of N-(4-phenyl-2,3-dihydrothiazol-2-ylidene)-(5-acetyl-3,6-dimethyl-1-phenyl-1H-pyrazolo[3,4-b]pyrazine)hydrazine (**23**).$CDCl_3$.*

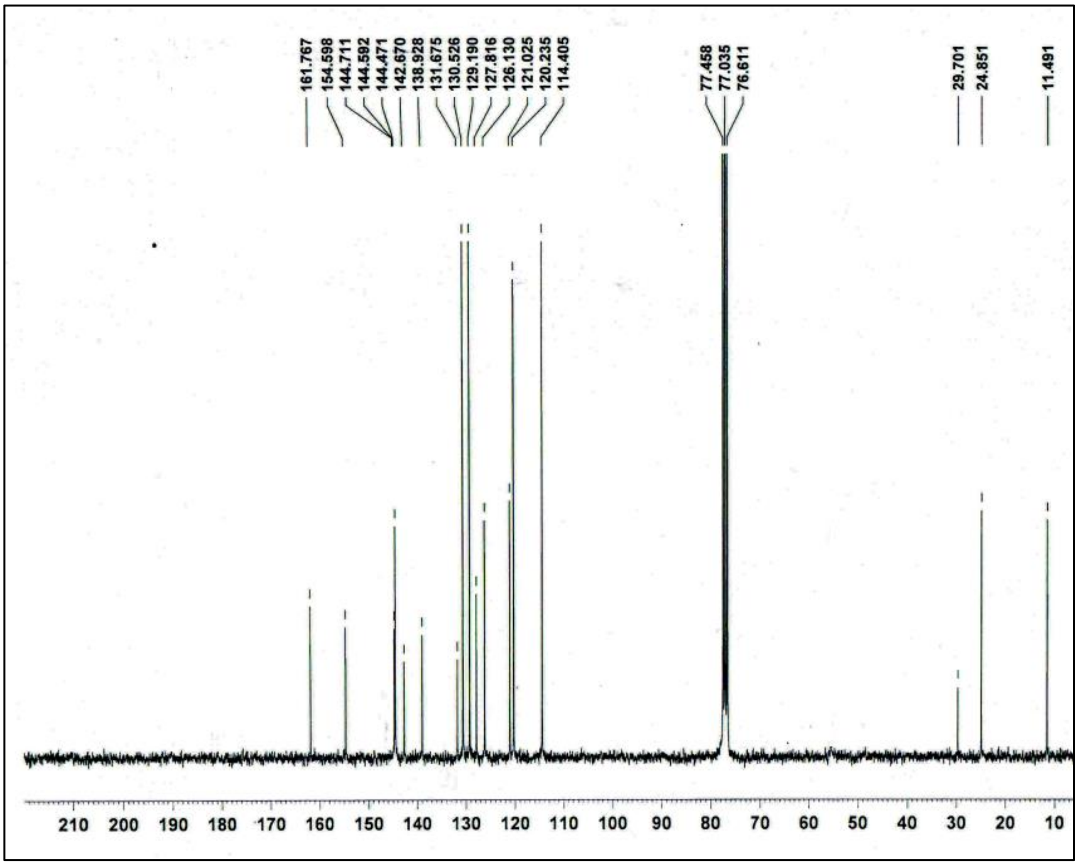

(Fig. 59), *^{13}C-NMR spectrum of N-(4-phenyl-2,3-dihydrothiazol-2-ylidene)-(5-acetyl-3,6-dimethyl-1-phenyl-1H-pyrazolo[3,4-b]pyrazine)hydrazine (**23**).CDCl$_3$.*

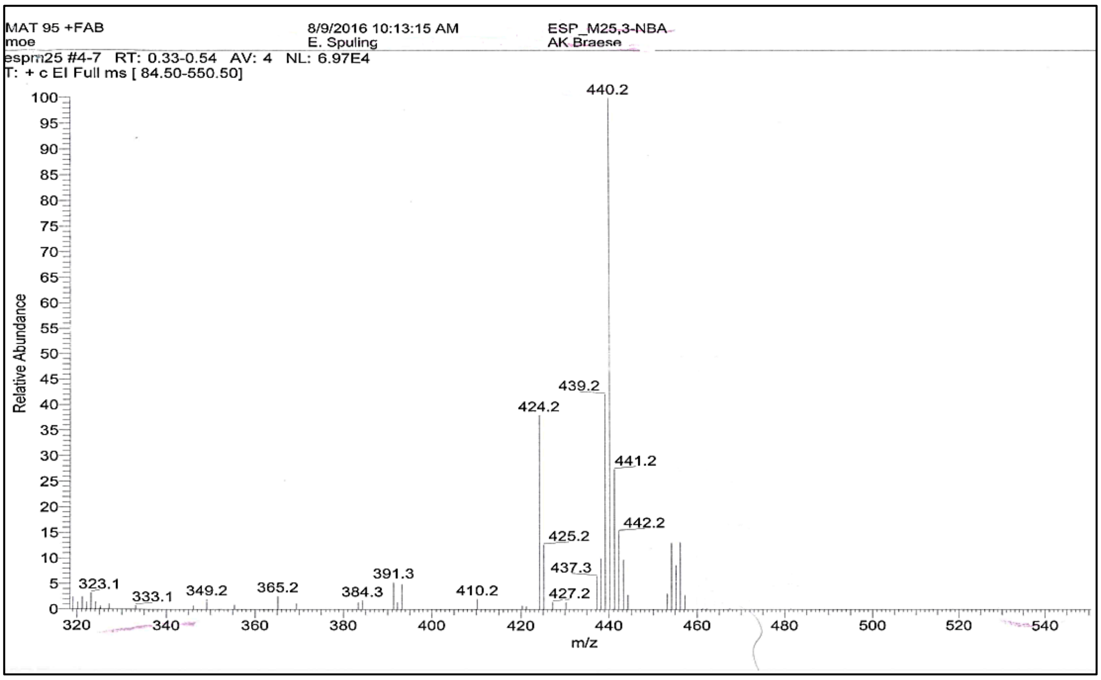

(Fig. 60), *Mass spectrum of N-(4-phenyl-2,3-dihydrothiazol-2-ylidene)-(5-acetyl-3,6-dimethyl-1-phenyl-1H-pyrazolo[3,4-b]pyrazine)hydrazine (**23**).*

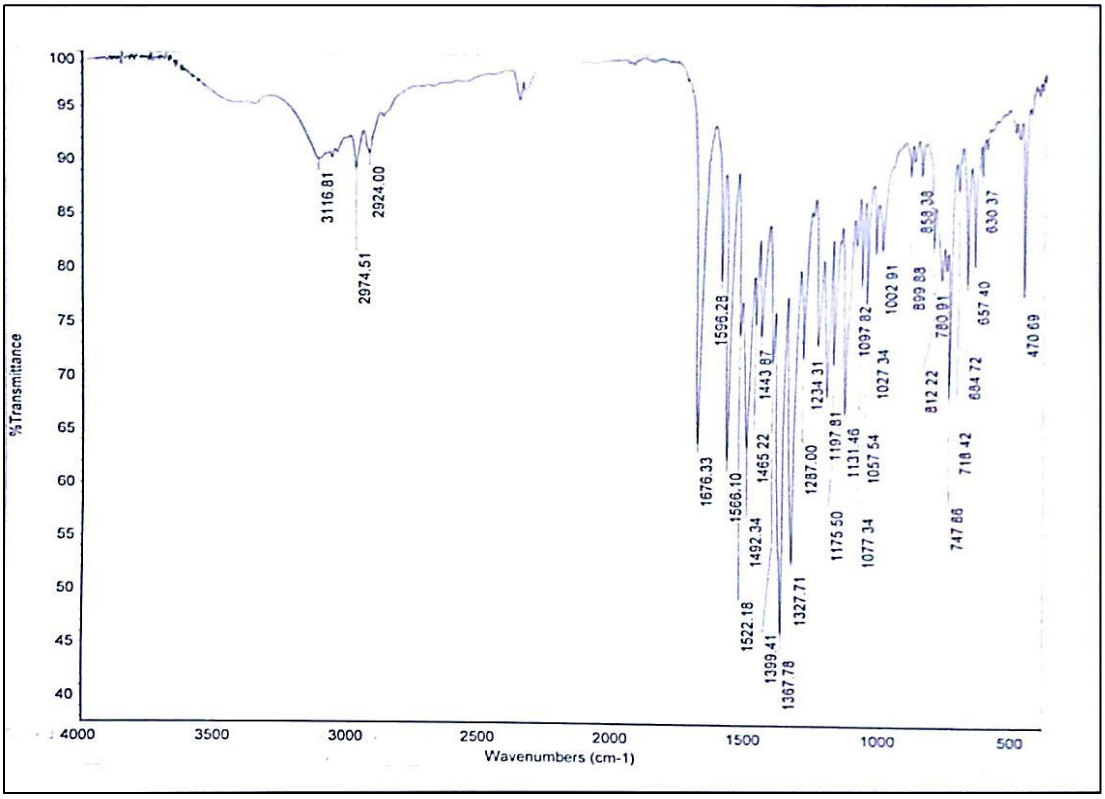

(Fig. 61), *IR spectrum of N-(thiazolidin-4-on-2-ylidene)-(5-acetyl-3,6-dimethyl-1-phenyl-1H-pyrazolo[3,4-b] pyrazine)hydrazine (**24**).*

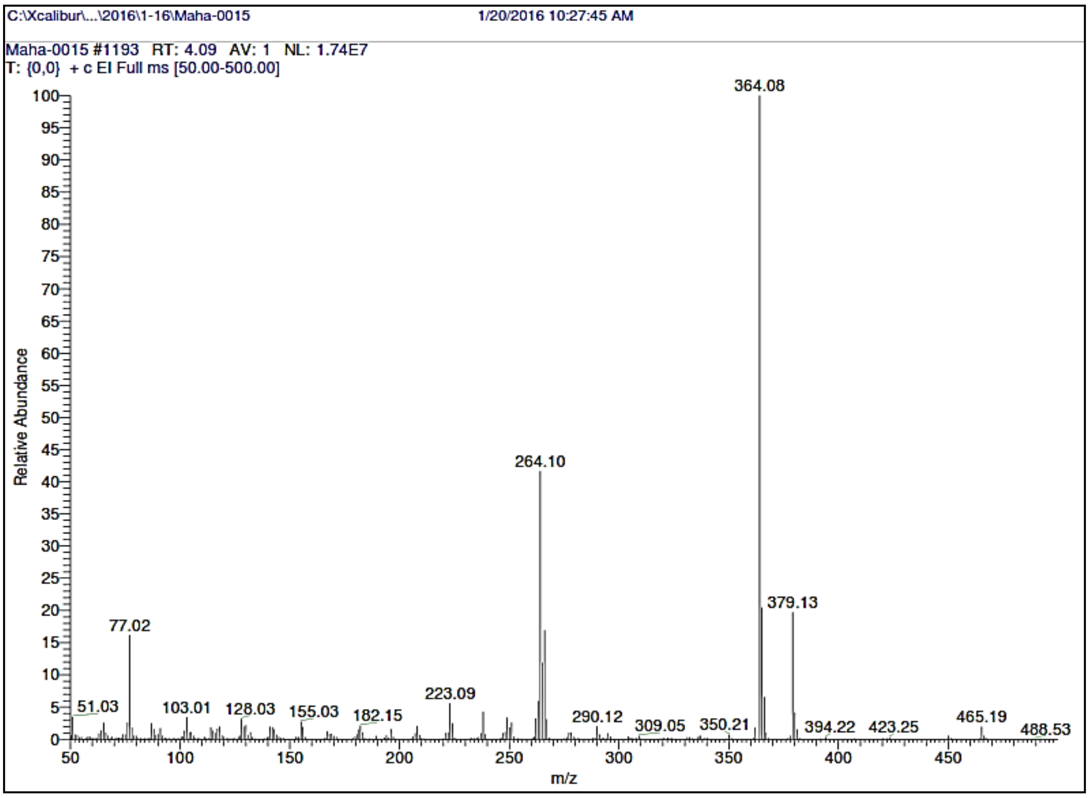

(Fig. 62), *Mass spectrum of N-(thiazolidin-4-on-2-ylidene)-(5-acetyl-3,6-dimethyl-1-phenyl-1H-pyrazolo[3,4-b]pyrazine)hydrazine (**24**).*

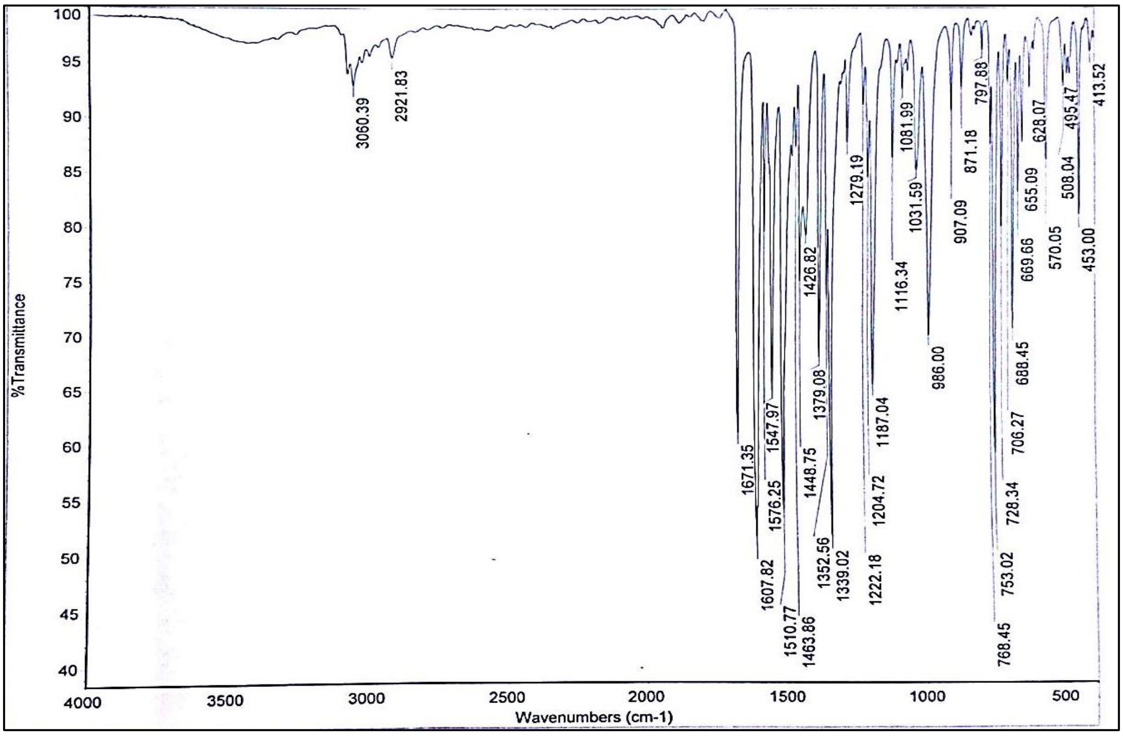

*(Fig. 63), IR spectrum of 5-benzylideneacetyl-3,6-dimethyl-1-phenyl-1H-pyrazolo[3,4-b]pyrazine (**25a**).*

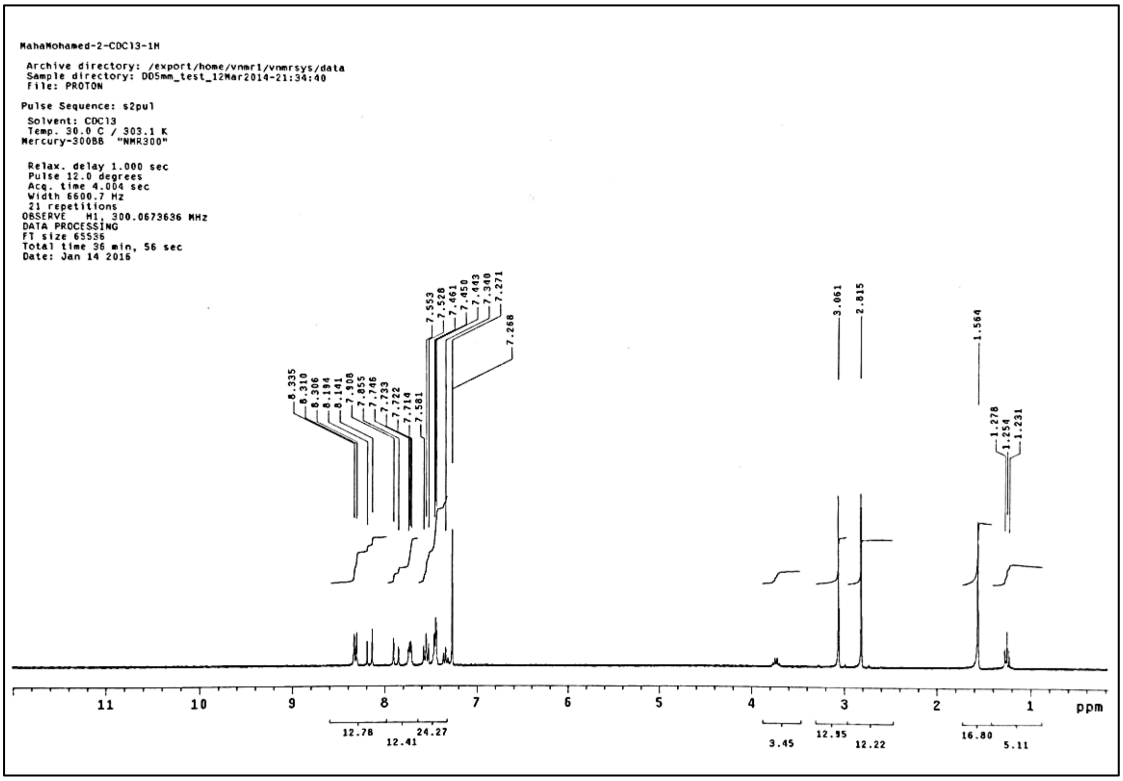

*(Fig. 64), ^{1}H-NMR spectrum of 5-benzylideneacetyl-3,6-dimethyl-1-phenyl-1H-pyrazolo[3,4-b]pyrazine (**25a**).$CDCl_3$.*

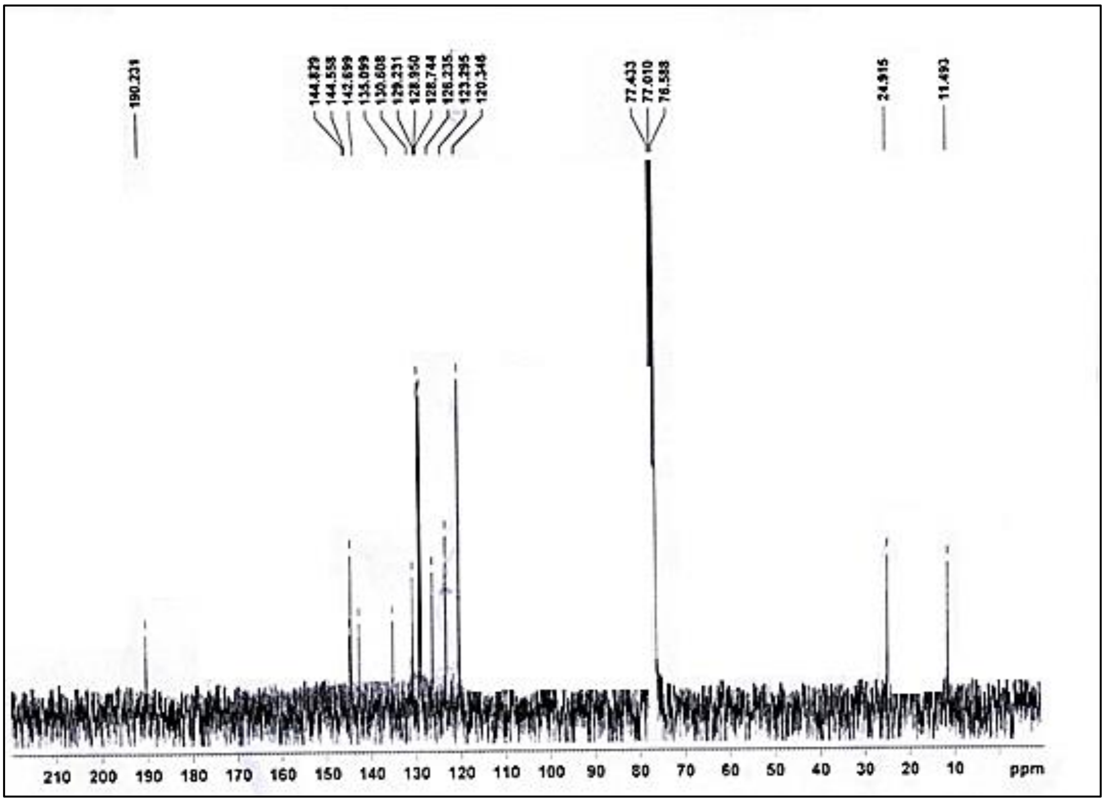

(Fig. 65), *^{13}C-NMR spectrum of 5-benzylideneacetyl-3,6-dimethyl-1-phenyl-1H-pyrazolo[3,4-b]pyrazine (**25a**).CDCl$_3$*

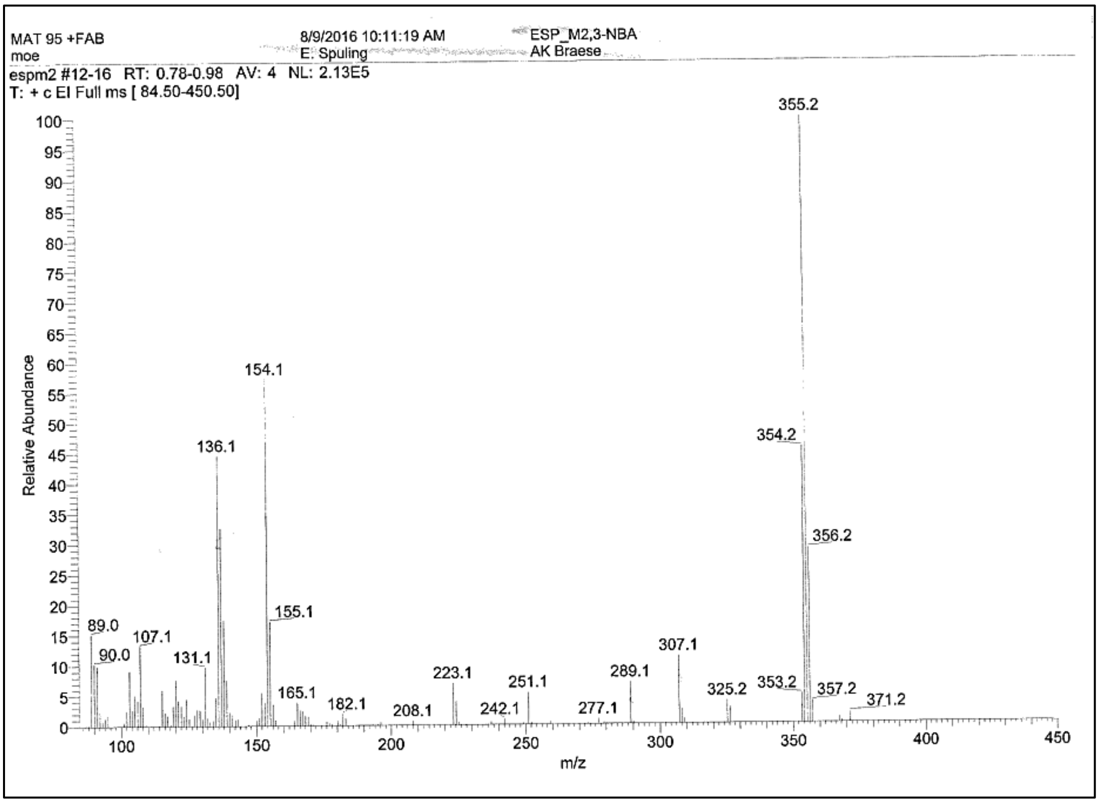

(Fig. 66), *Mass spectrum of 5-benzylideneacetyl-3,6-dimethyl-1-phenyl-1H-pyrazolo[3,4-b]pyrazine (**25a**).*

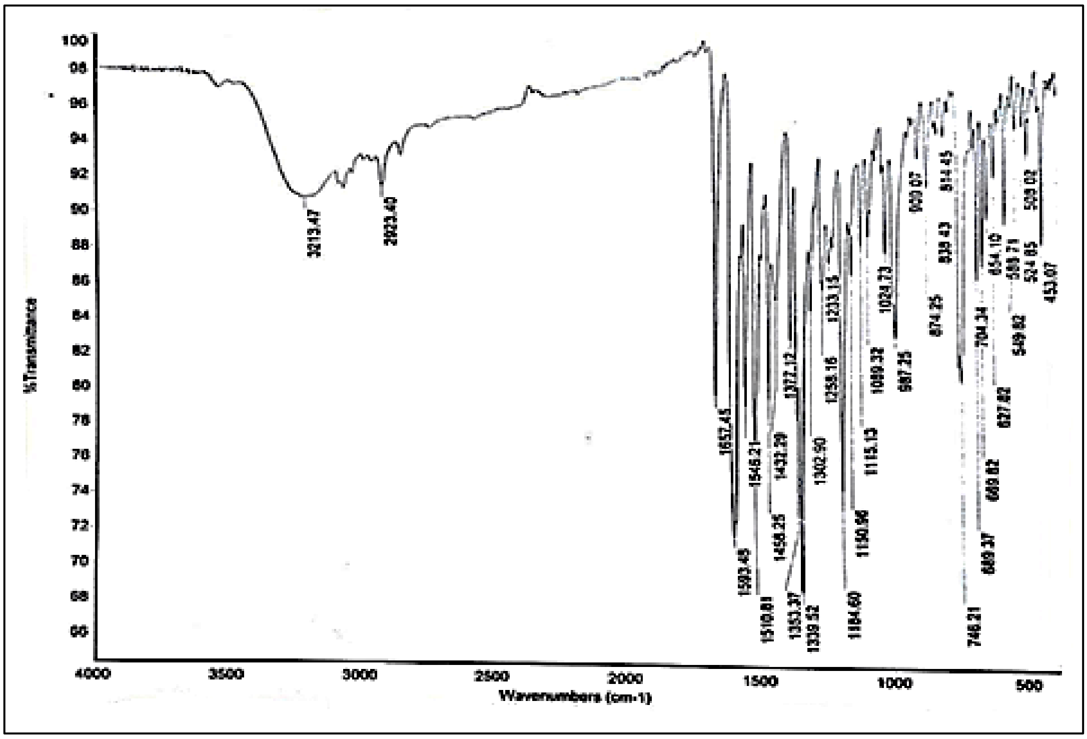

(Fig. 67), *IR spectrum of 3,6-dimethyl-5-(2-hydroxylbenzylideneacetyl)-1-phenyl-1H-pyrazolo[3,4-b] pyrazine (**25b**).*

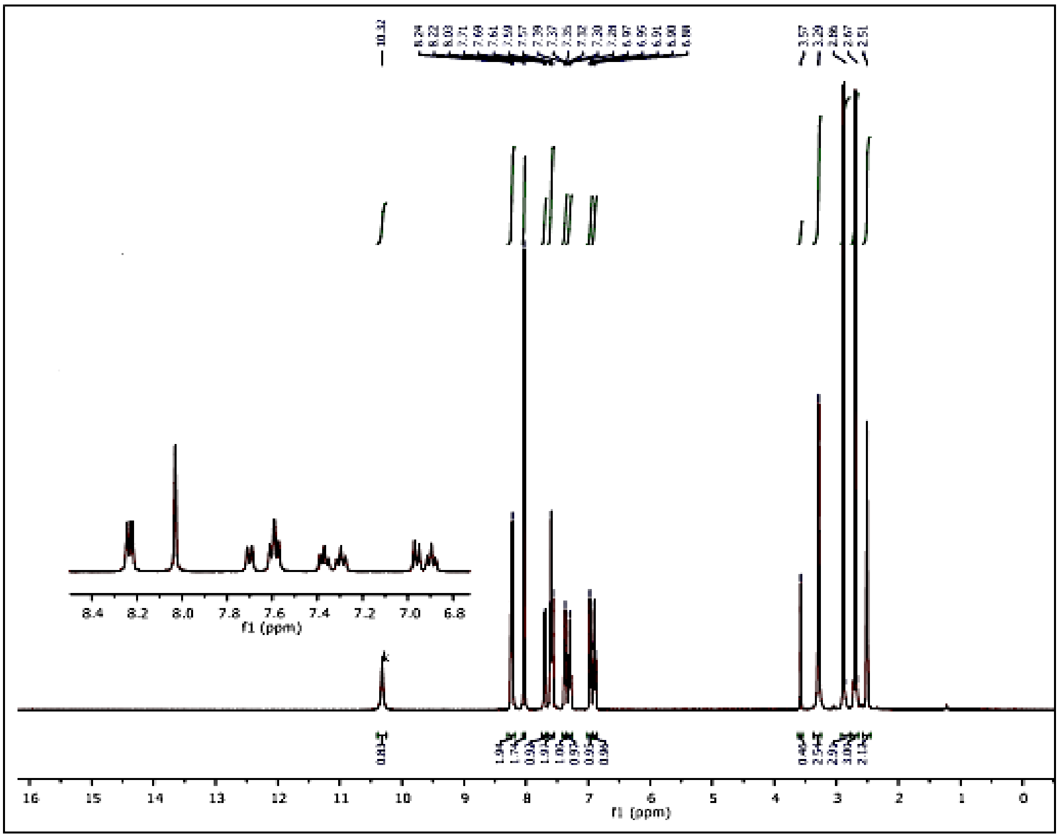

(Fig. 68), *^{1}H-NMR spectrum of 3,6-dimethyl-5-(2-hydroxylbenzylideneacetyl)-1-phenyl-1H-pyrazolo[3,4-b] pyrazine (**25b**).* DMSO-d_6

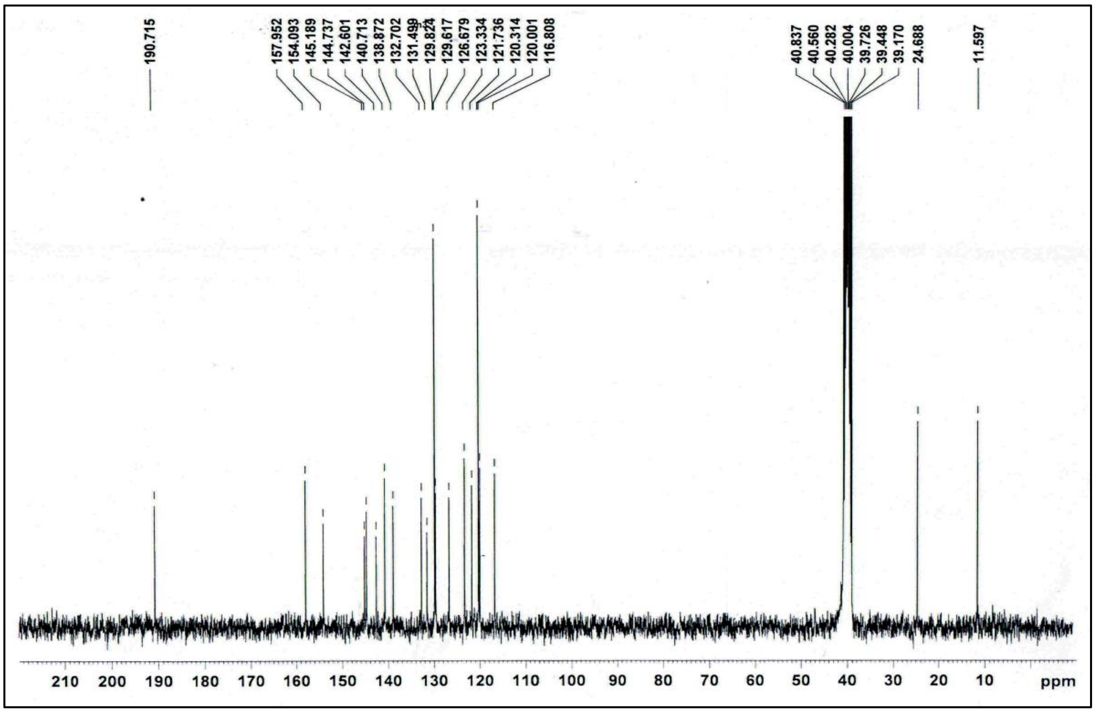

(Fig. 69), *^{13}C-NMR spectrum of 3,6-dimethyl-5-(2-hydroxylbenzylideneacetyl)-1-phenyl-1H-pyrazolo[3,4-b]pyrazine (**25b**).* DMSO-d_6

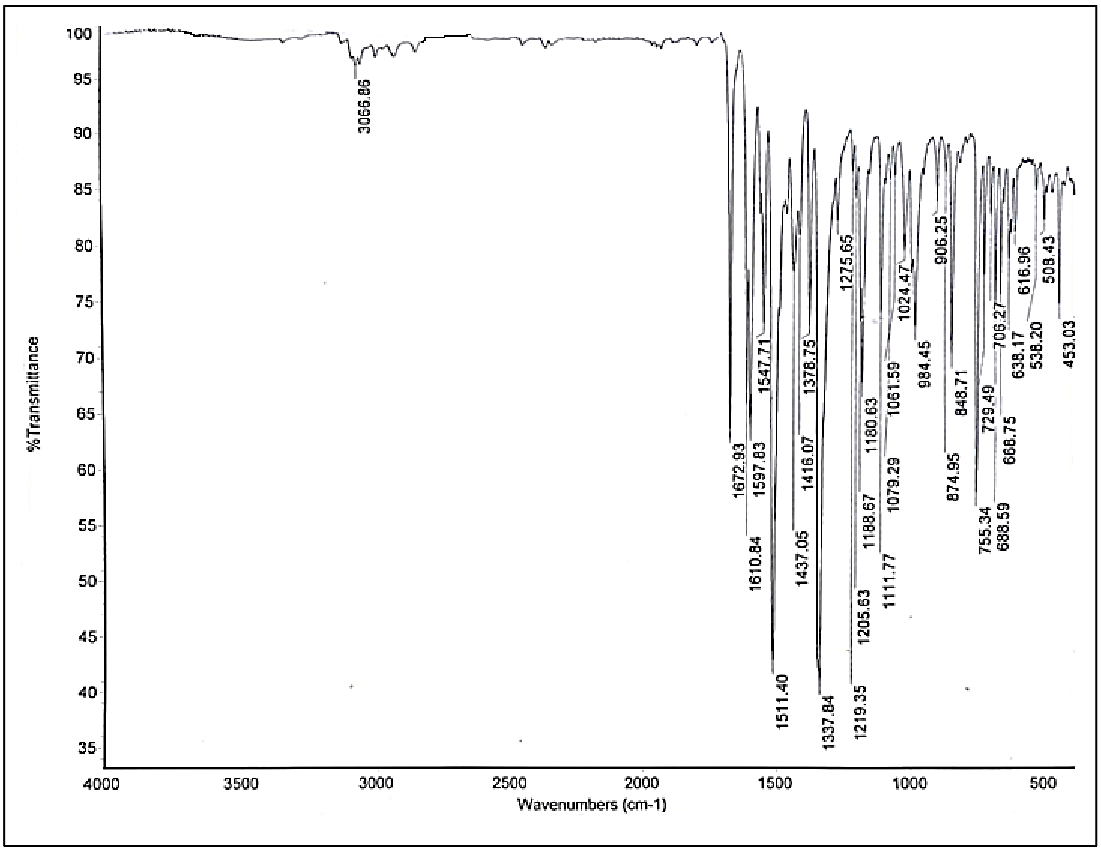

(Fig. 70), *IR spectrum of 3,6-dimethyl-5-(2-nitrobenzylideneacetyl)-1-phenyl-1H-pyrazolo[3,4-b]pyrazine (**25c**).*

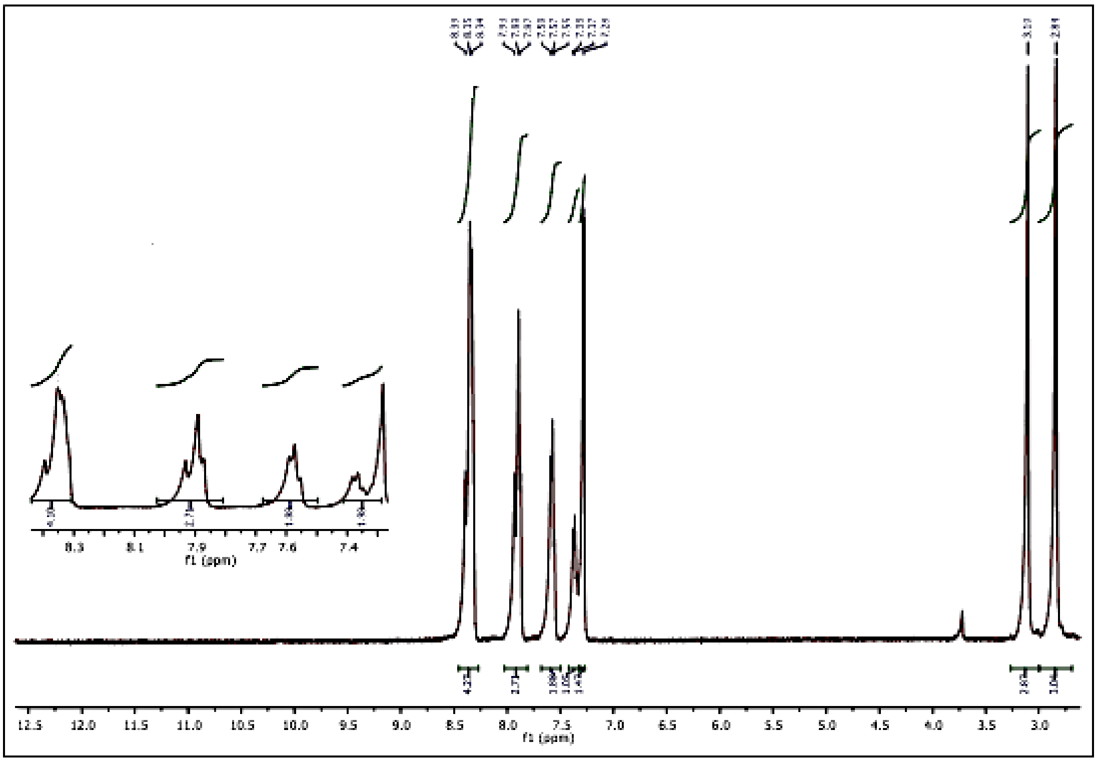

(Fig. 71), *^{1}H-NMR spectrum of 3,6-dimethyl-5-(2-nitrobenzylideneacetyl)-1-phenyl-1H-pyrazolo[3,4-b]pyrazine (**25c**).CDCl$_3$.*

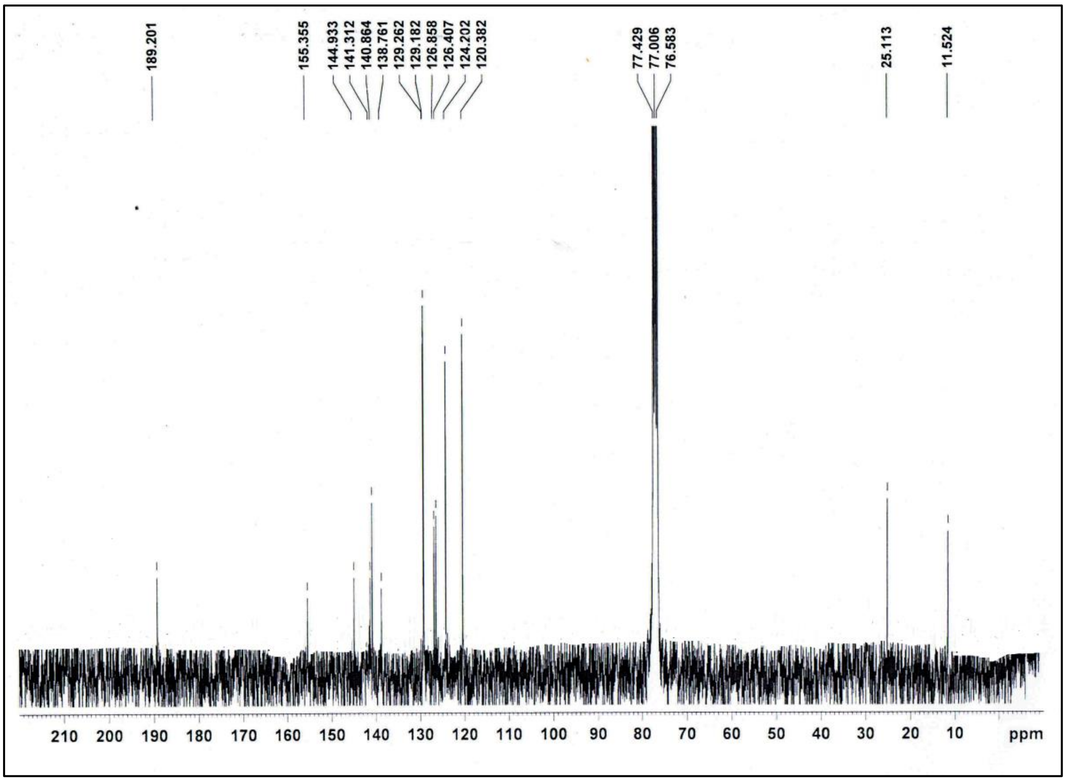

(Fig. 72), *^{13}C-NMR spectrum of 3,6-dimethyl-5-(2-nitrobenzylideneacetyl)-1-phenyl-1H-pyrazolo[3,4-b]pyrazine (**25c**).CDCl$_3$.*

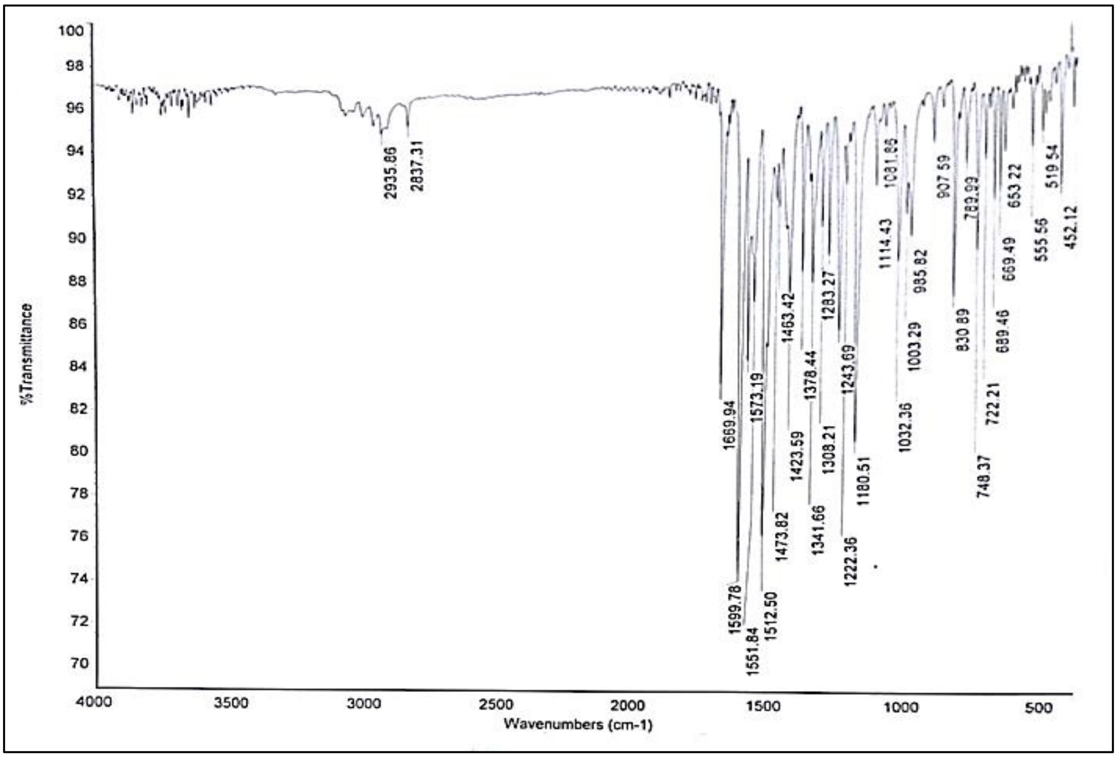

(Fig. 73), *IR spectrum of 3,6-dimethyl-5-(4-nitrobenzylideneacetyl)-1-phenyl-1H-pyrazolo[3,4-b]pyrazine (**25d**).*

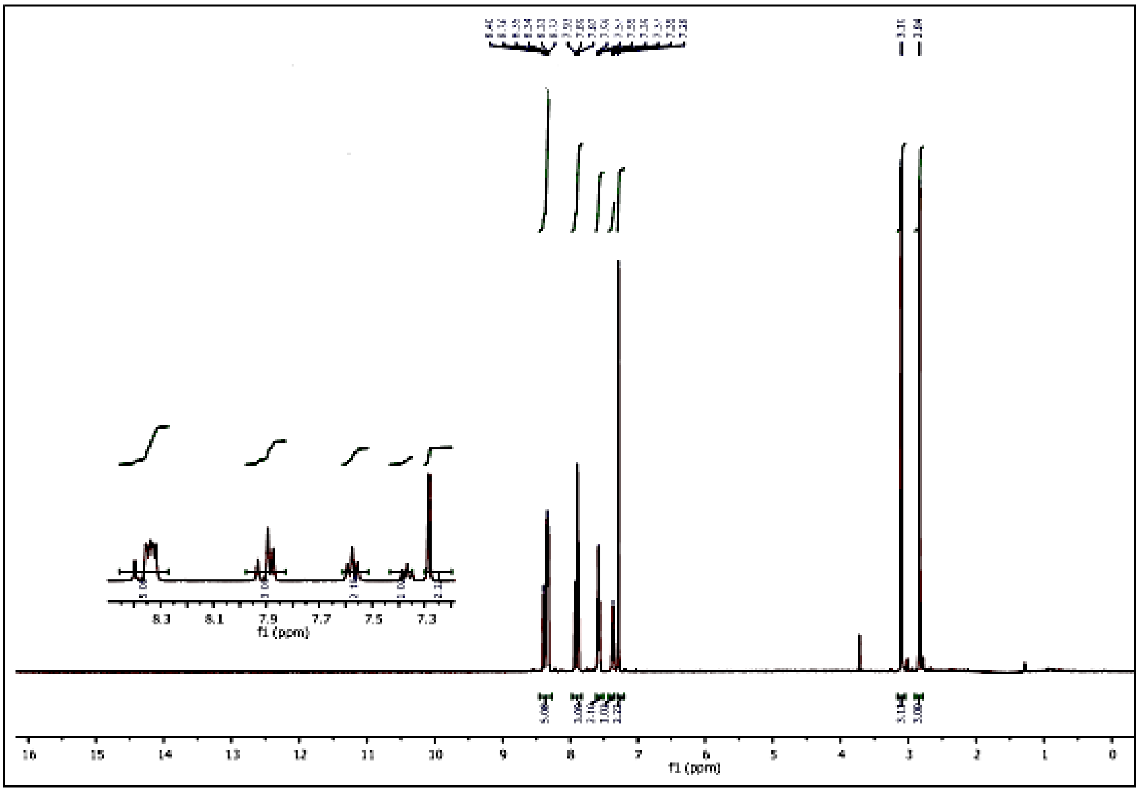

(Fig. 74), *^{1}H-NMR spectrum of 3,6-dimethyl-5-(4-nitrobenzylideneacetyl)-1-phenyl-1H-pyrazolo[3,4-b]pyrazine (**25d**).$CDCl_3$.*

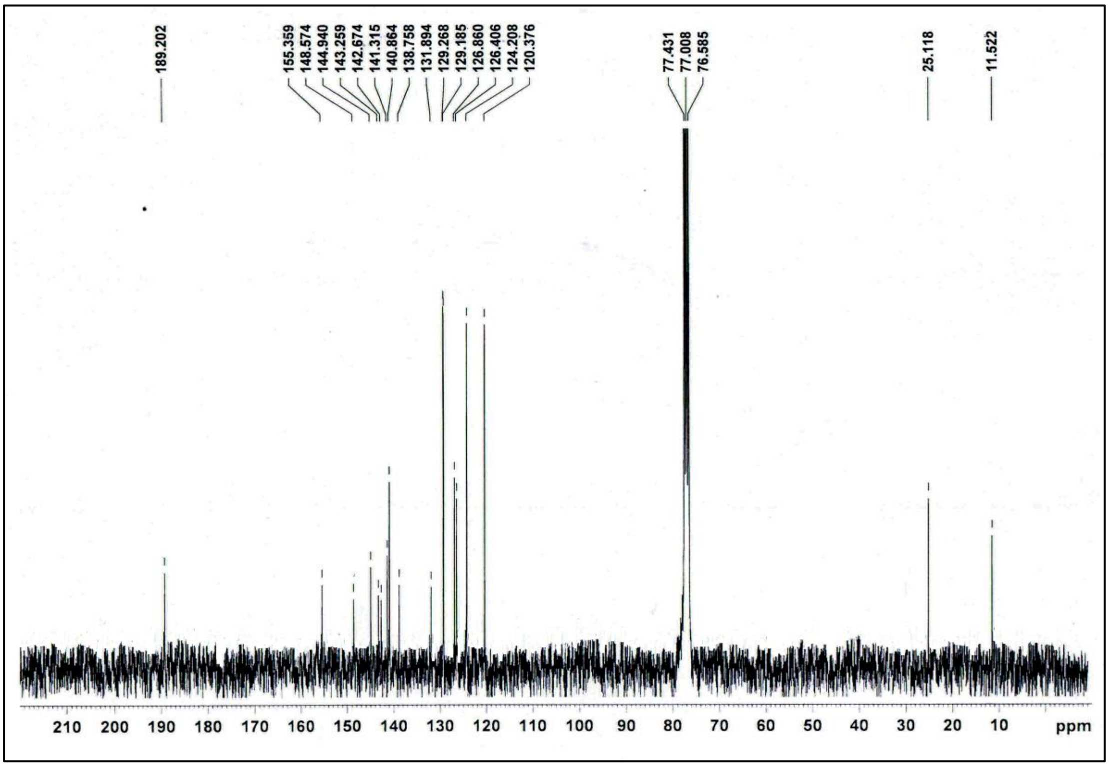

(Fig. 75), *^{13}C-NMR spectrum of 3,6-dimethyl-5-(4-nitrobenzylideneacetyl)-1-phenyl-1H-pyrazolo[3,4-b]pyrazine (**25d**).CDCl*$_3$.

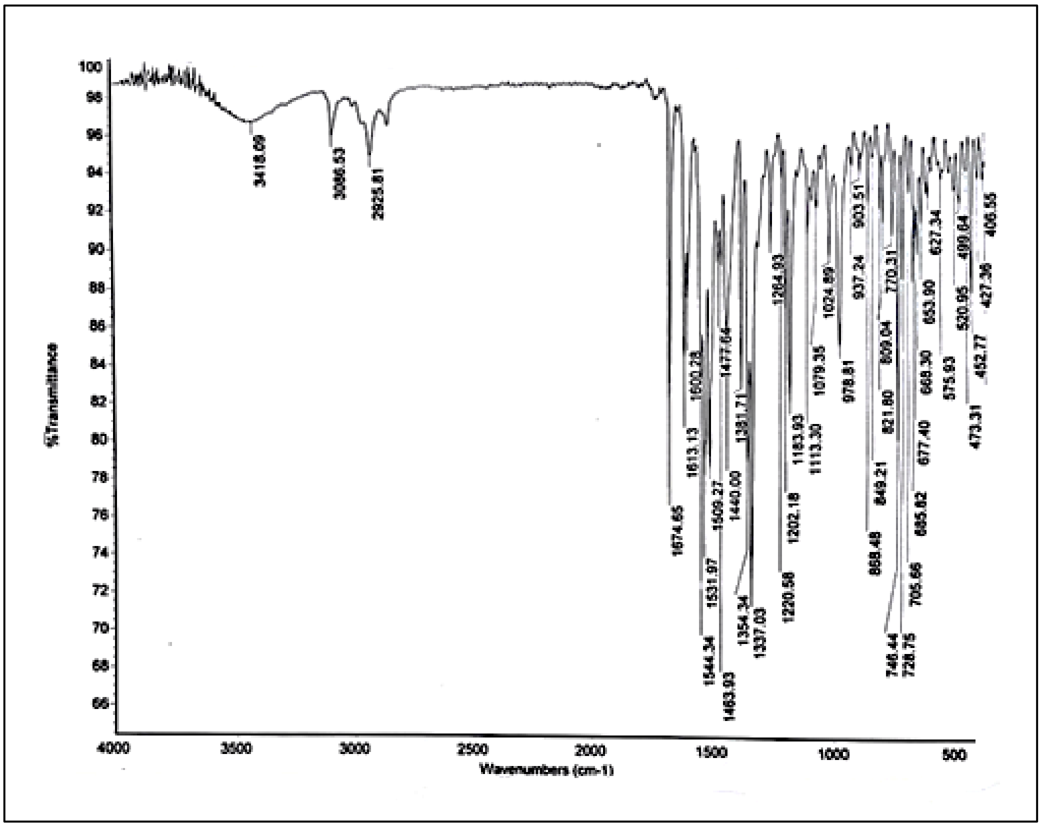

(Fig. 76), *IR spectrum of 3,6-dimethyl-5-(3-nitrobenzylideneacetyl)-1-phenyl-1H-pyrazolo[3,4-b]pyrazine (**25e**).*

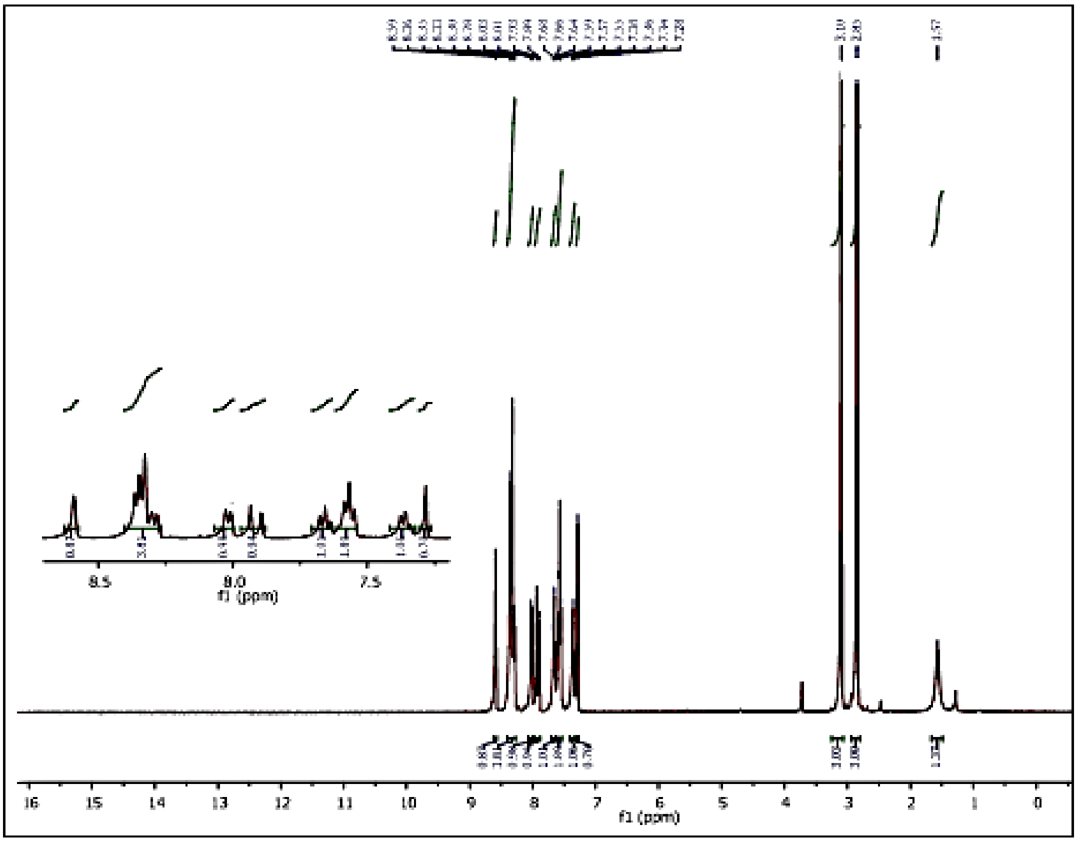

(Fig. 77), *^{1}H-NMR spectrum of 3,6-dimethyl-5-(3-nitrobenzylideneacetyl)-1-phenyl-1H-pyrazolo[3,4-b]pyrazine (**25e**).CDCl$_3$*

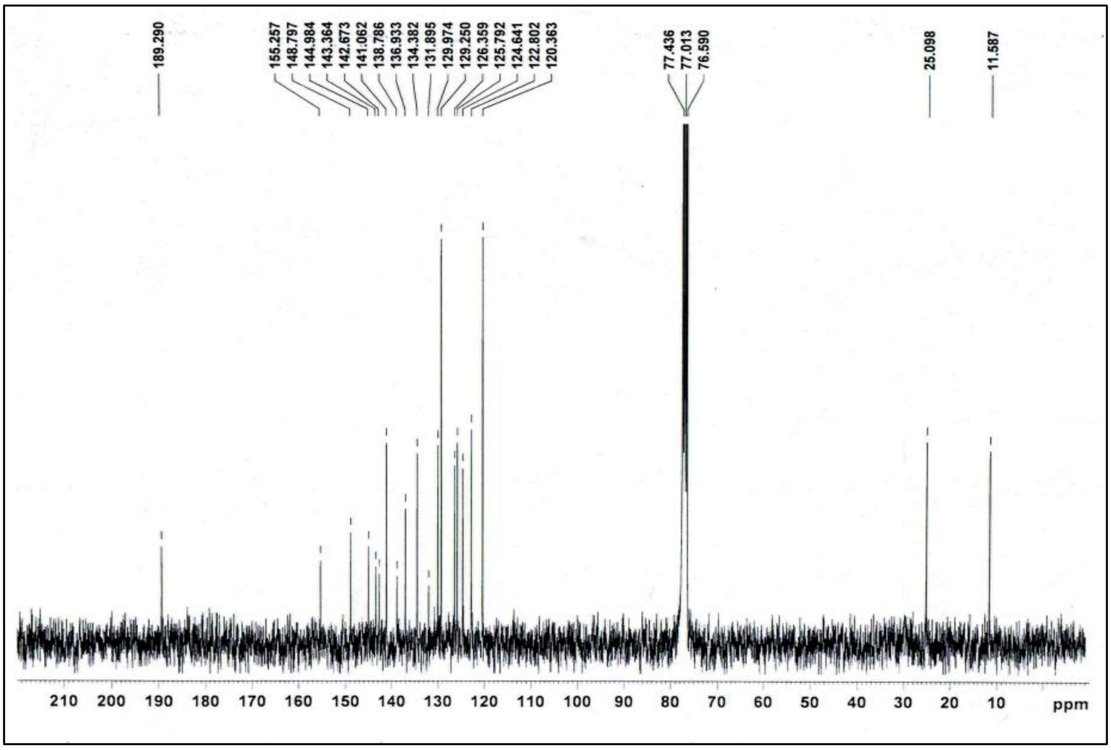

(Fig. 78), *^{13}C-NMR spectrum of 3,6-dimethyl-5-(3-nitrobenzylideneacetyl)-1-phenyl-1H-pyrazolo[3,4-b]pyrazine (**25e**).CDCl$_3$*

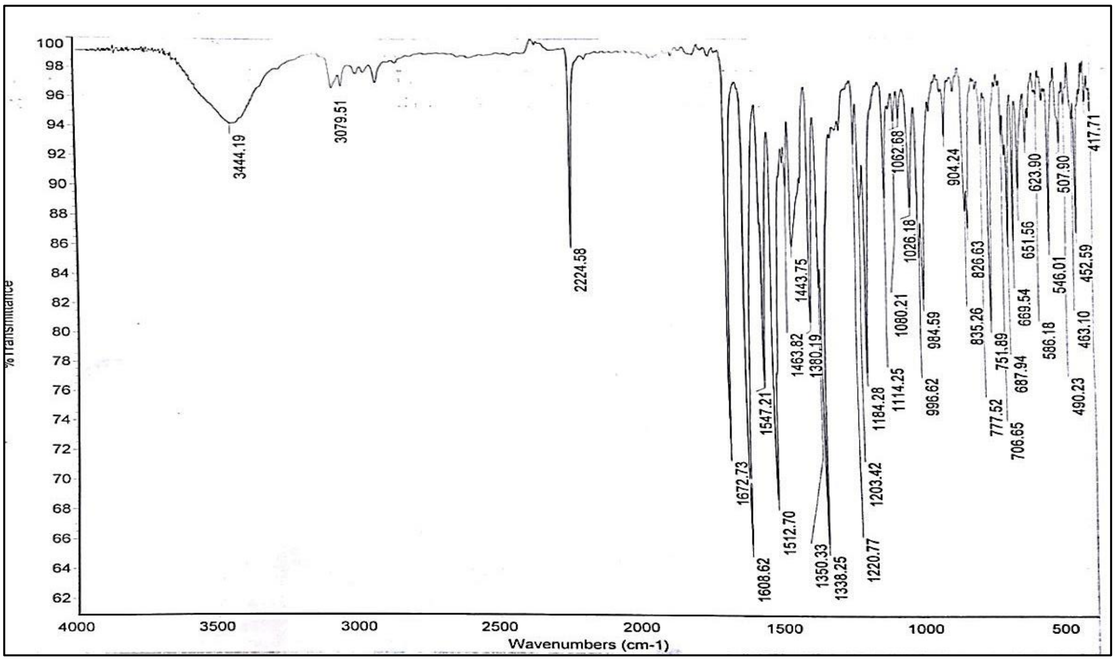

(Fig. 79), *IR spectrum of 3,6-dimethyl-5-(4-cyanobenzylideneacetyl)-1-phenyl-1H-pyrazolo[3,4-b]pyrazine (**25f**)*

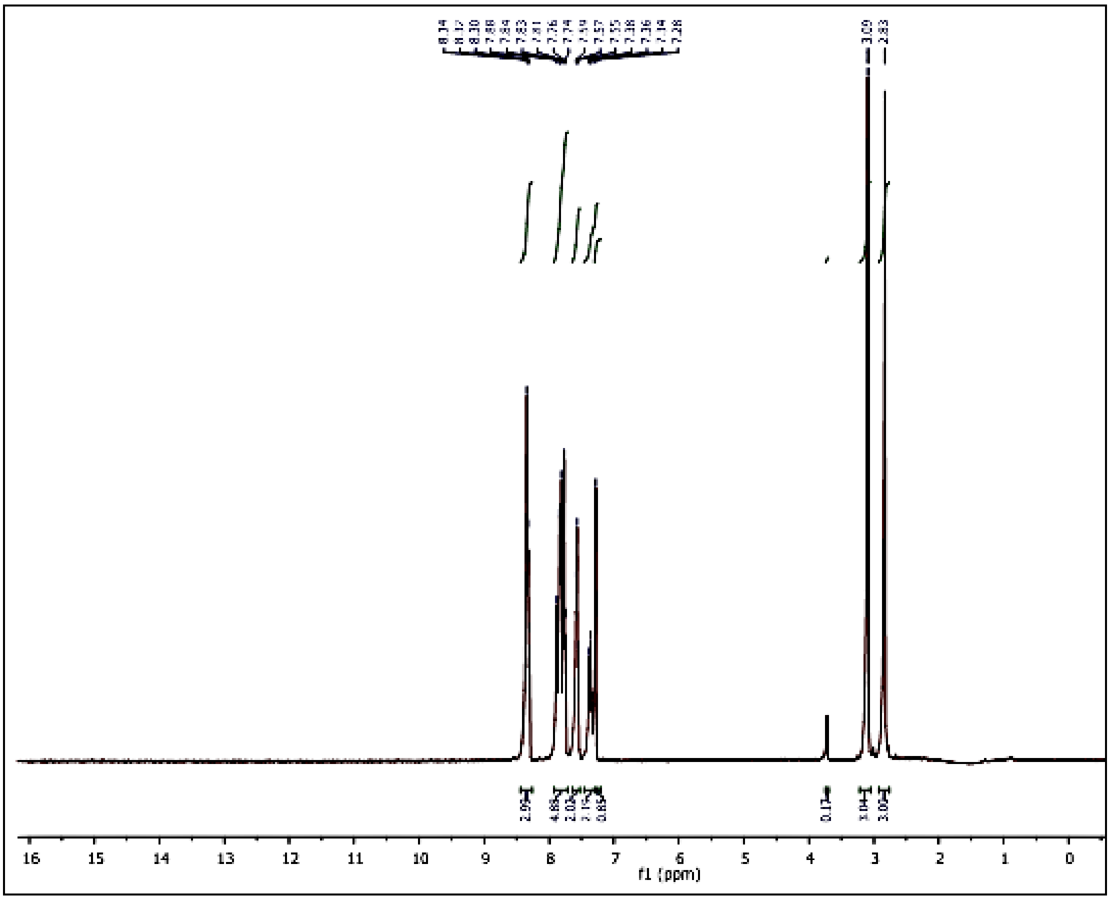

(Fig. 80), *^{1}H-NMR spectrum of 3,6-dimethyl-5-(4-cyanobenzylideneacetyl)-1-phenyl-1H-pyrazolo[3,4-b]pyrazine (**25f**).CDCl$_3$*

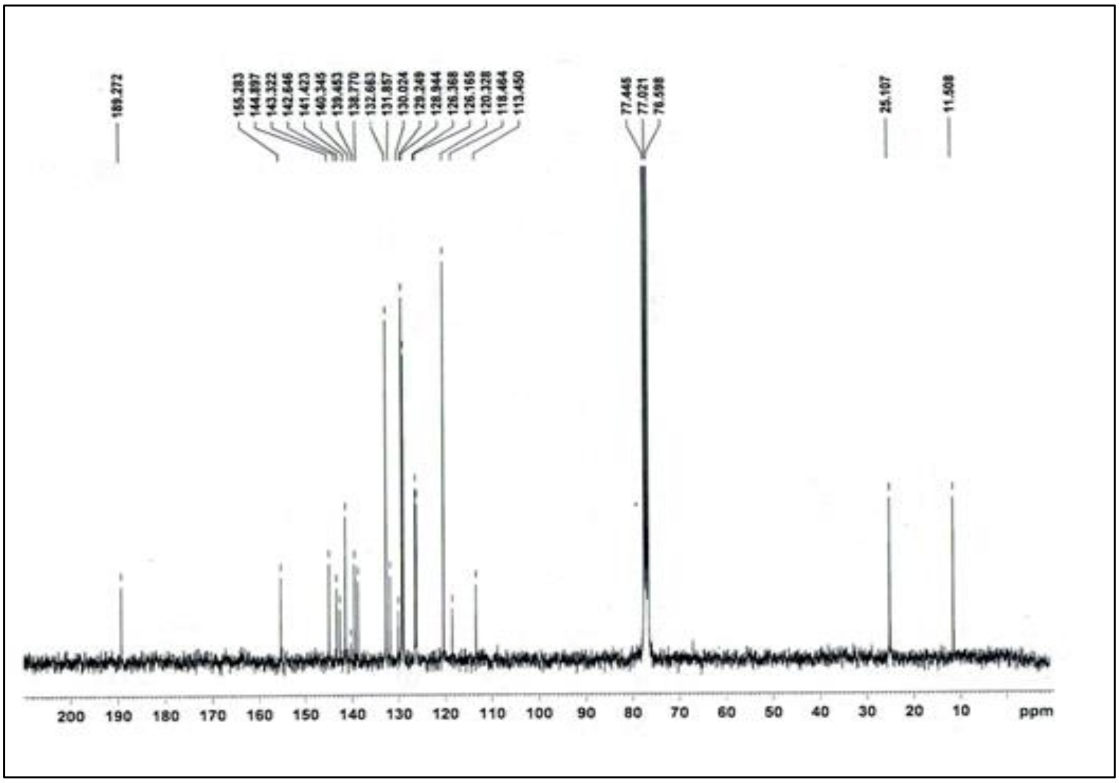

(Fig. 81), *^{13}C-NMR spectrum of 3,6-dimethyl-5-(4-cyanobenzylideneacetyl)-1-phenyl-1H-pyrazolo[3,4-b]pyrazine (**25f**).CDCl$_3$*

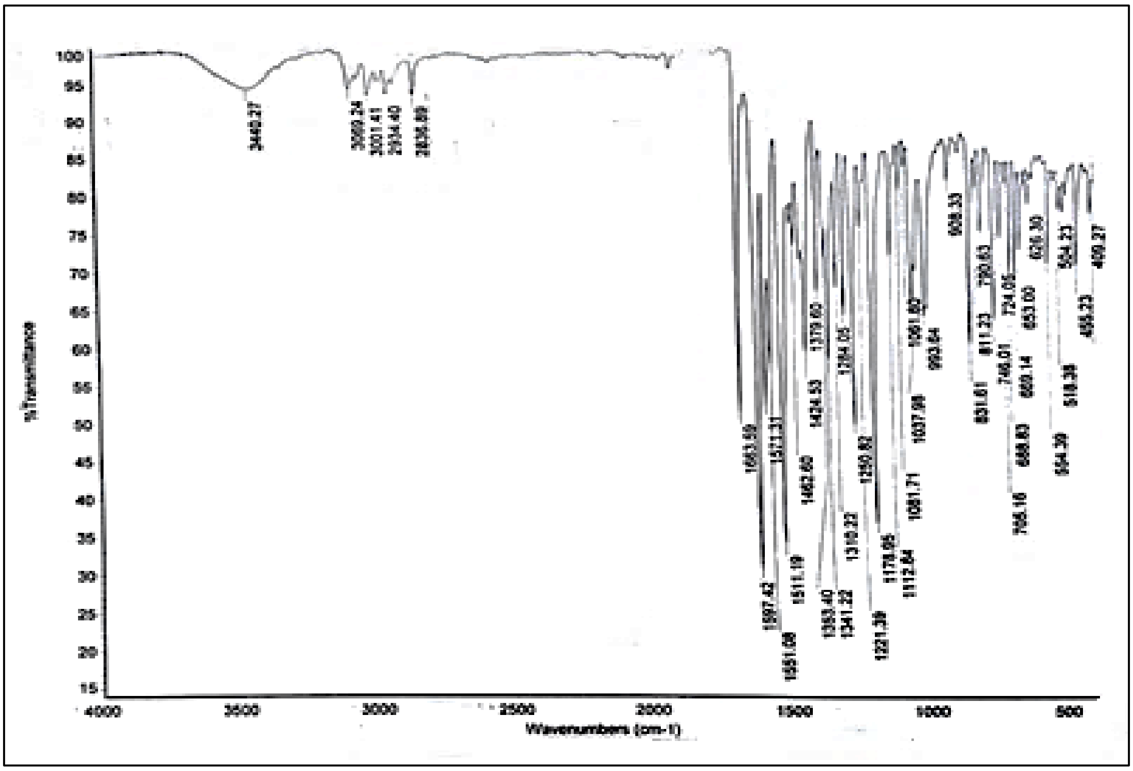

(Fig. 82), *IR spectrum of 3,6-dimethyl-5-(4-methoxybenzylideneacetyl)-1-phenyl-1H-pyrazolo[3,4-b]pyrazine (**25g**).*

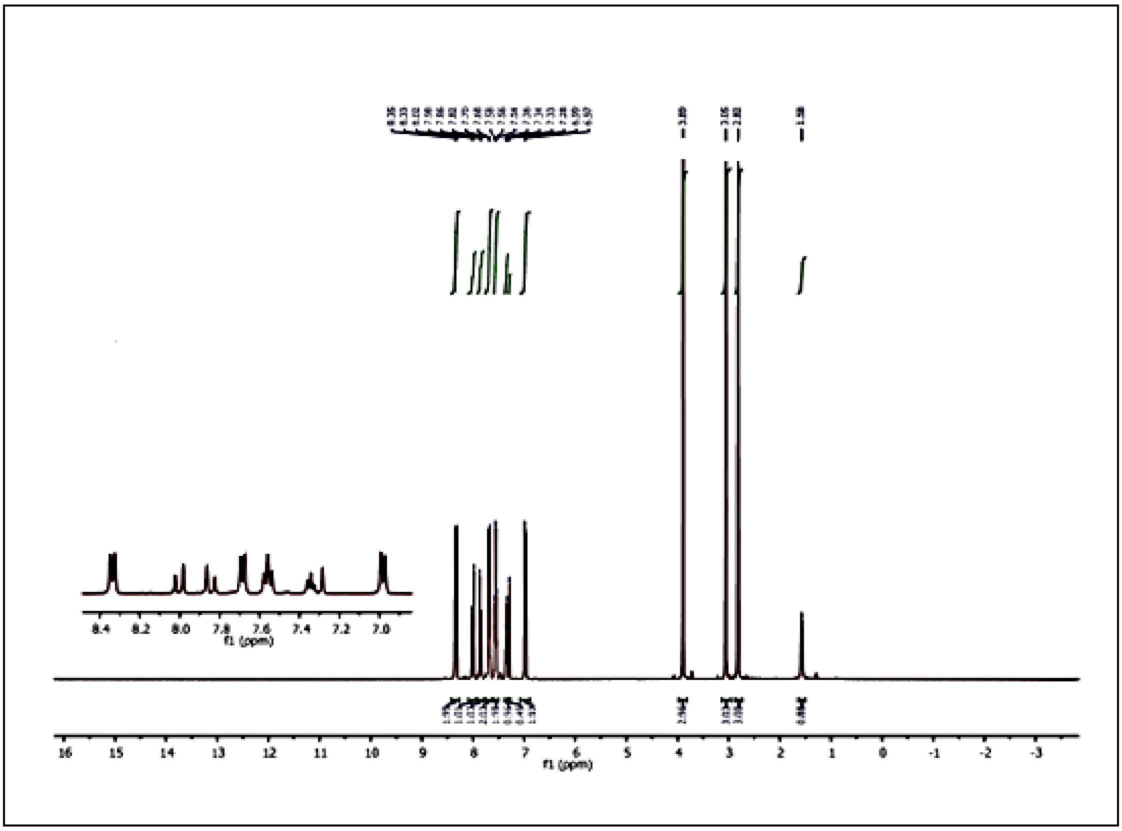

(Fig. 83), *^{1}H-NMR spectrum of 3,6-dimethyl-5-(4-methoxybenzylideneacetyl)-1-phenyl-1H-pyrazolo[3,4-b]pyrazine (**25g**).*CDCl$_3$

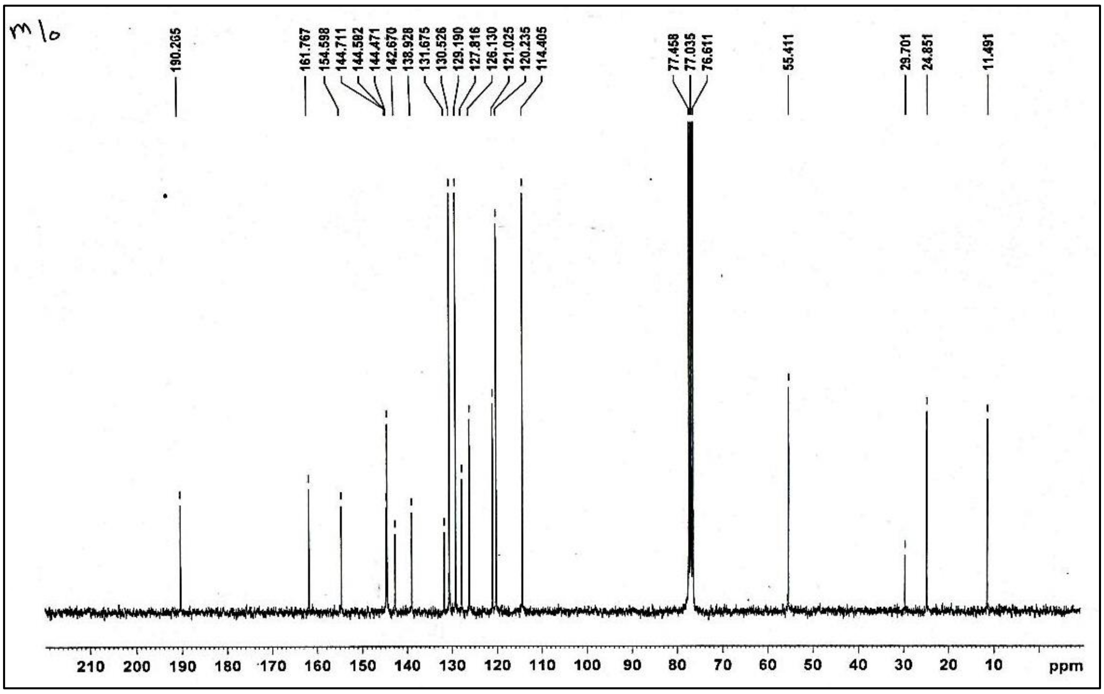

(Fig. 84), *^{13}C-NMR spectrum of 3,6-dimethyl-5-(4-methoxybenzylideneacetyl)-1-phenyl-1H-pyrazolo[3,4-b]pyrazine (**25g**).*CDCl$_3$

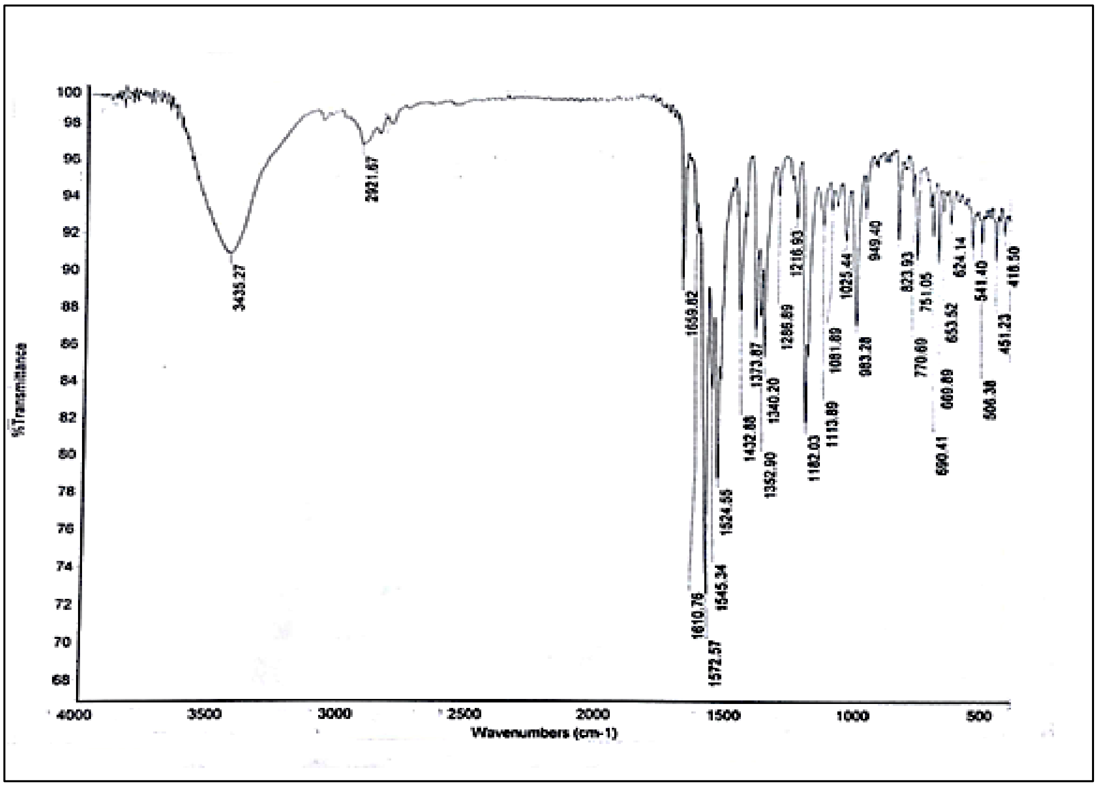

*(**Fig. 85**), IR spectrum of 3,6-dimethyl-5-(4-N,N-dimethylaminobenzylideneacetyl)-1-phenyl-1H-pyrazolo[3,4-b]pyrazine (**25h**).*

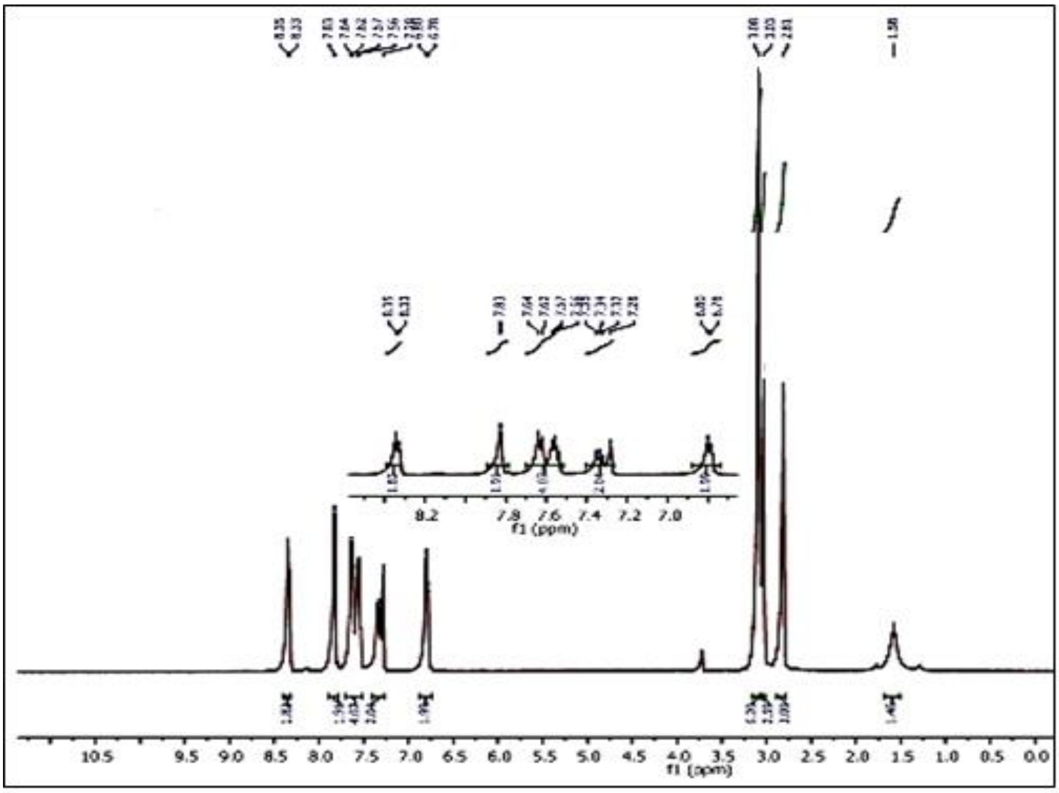

*(**Fig. 86**), ^{1}H-NMR spectrum of 3,6-dimethyl-5-(4-N,N-dimethylaminobenzylideneacetyl)-1-phenyl-1H-pyrazolo[3,4-b]pyrazine (**25h**).$CDCl_3$*

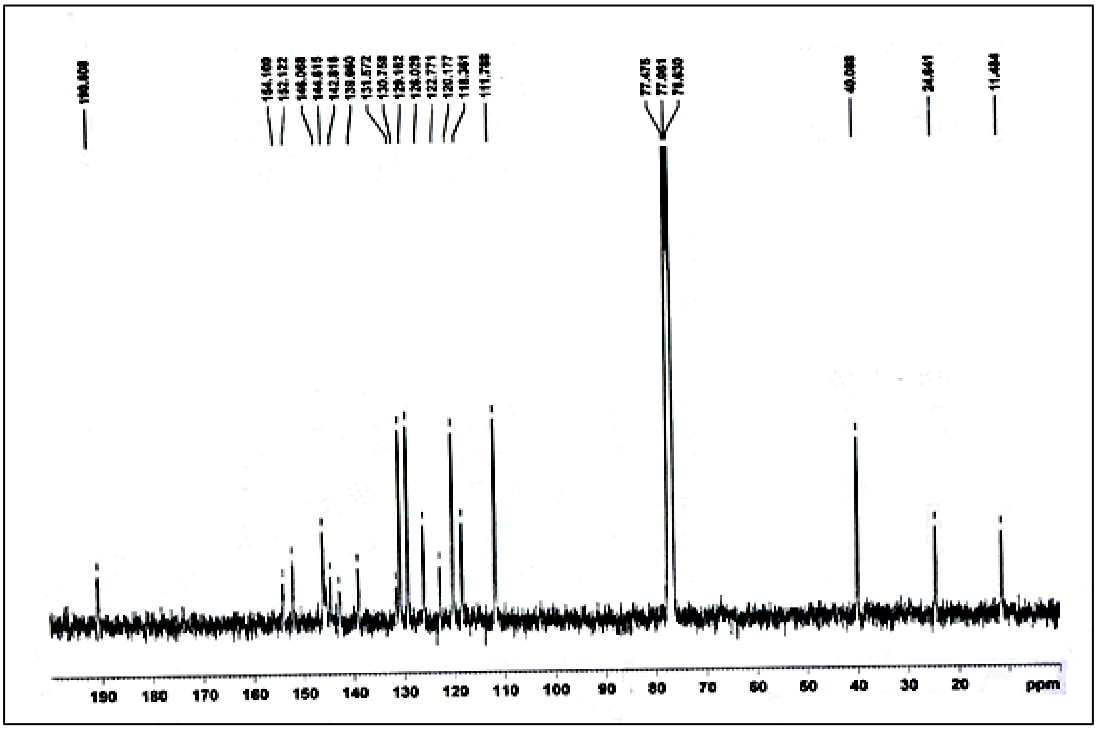

(Fig. 87), *^{13}C-NMR spectrum of 3,6-dimethyl-5-(4-N,N-dimethylaminobenzylideneacetyl)-1-phenyl-1H-pyrazolo[3,4-b]pyrazine (**25h**).$CDCl_3$*

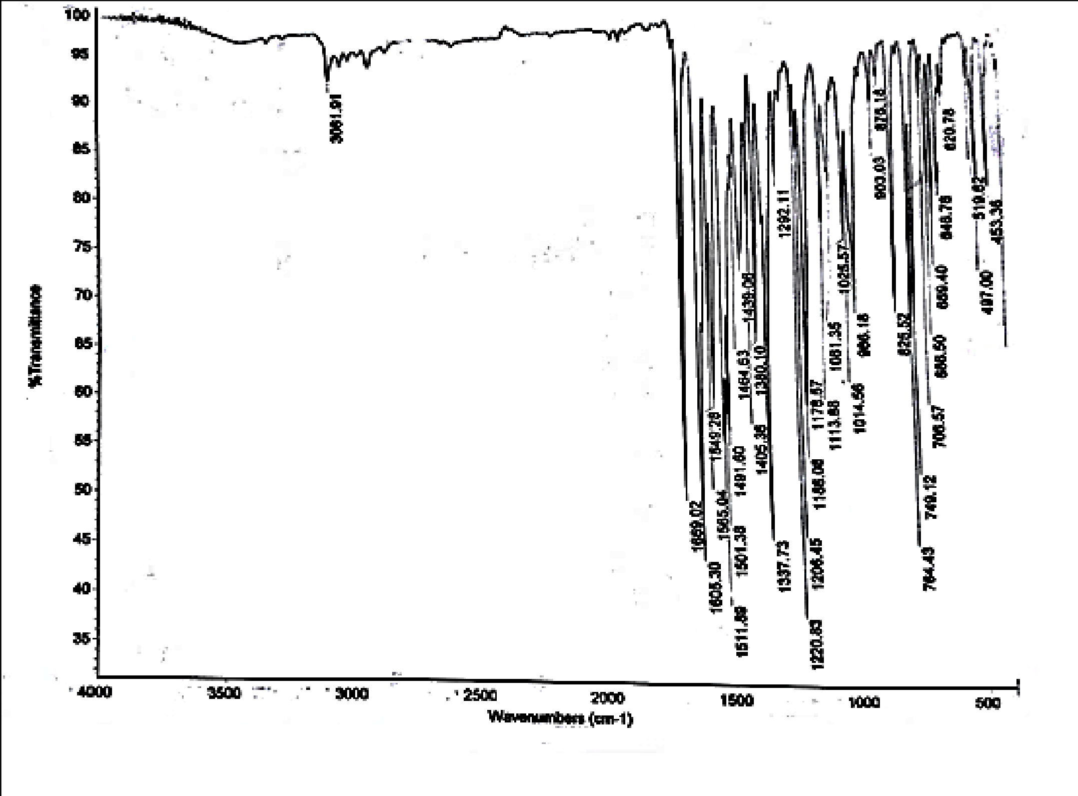

(Fig. 88), *IR spectrum of 3,6-dimethyl-5-(4-chlorobenzylideneacetyl)-1-phenyl-1H-pyrazolo[3,4-b]pyrazine **(25i)***

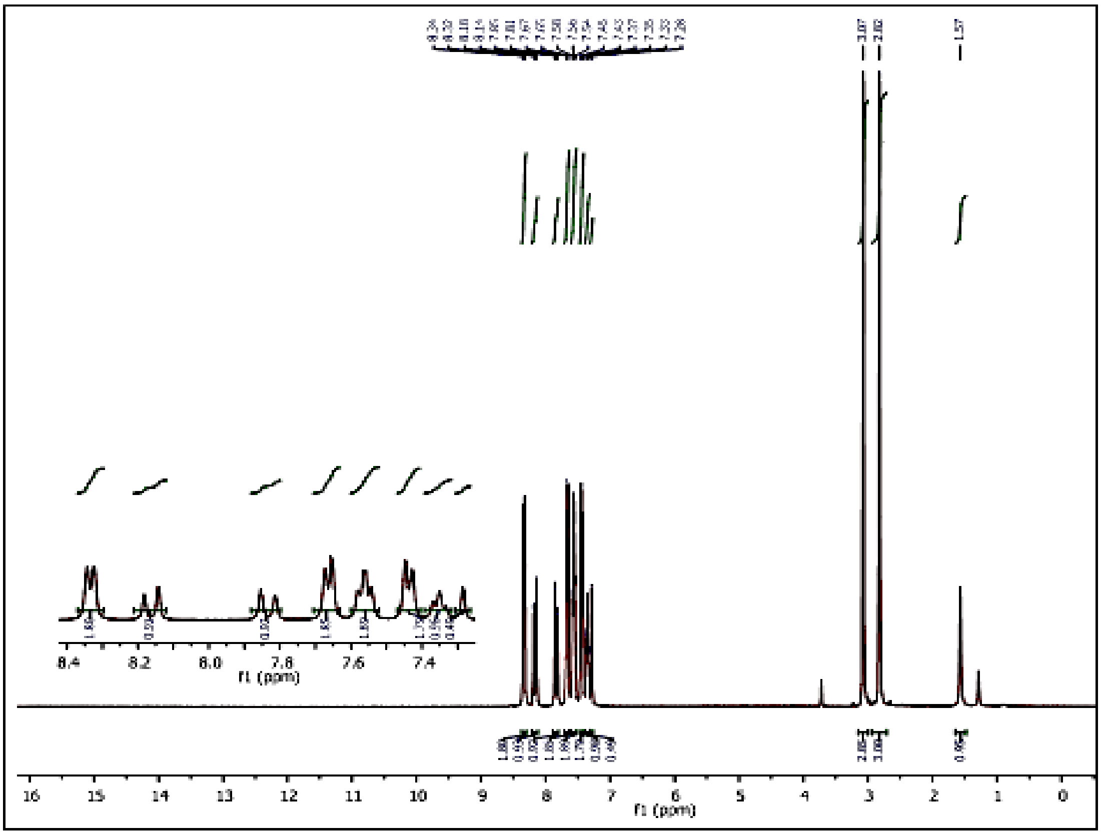

(Fig. 89), *^{1}H-NMR spectrum of 3,6-dimethyl-5-(4-chlorobenzylideneacetyl)-1-phenyl-1H-pyrazolo[3,4-b]pyrazine (****25i****). CDCl*$_3$*.*

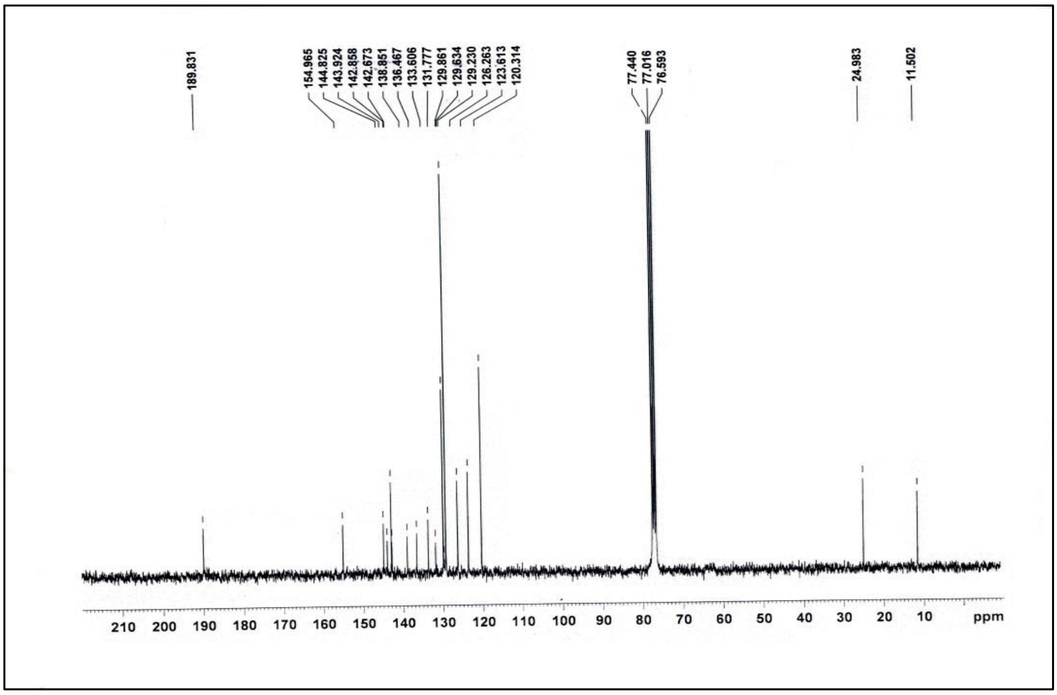

(Fig. 90), *^{13}C-NMR spectrum of 3,6-dimethyl-5-(4-chlorobenzylideneacetyl)-1-phenyl-1H-pyrazolo[3,4-b]pyrazine (****25i****). CDCl*$_3$*.*

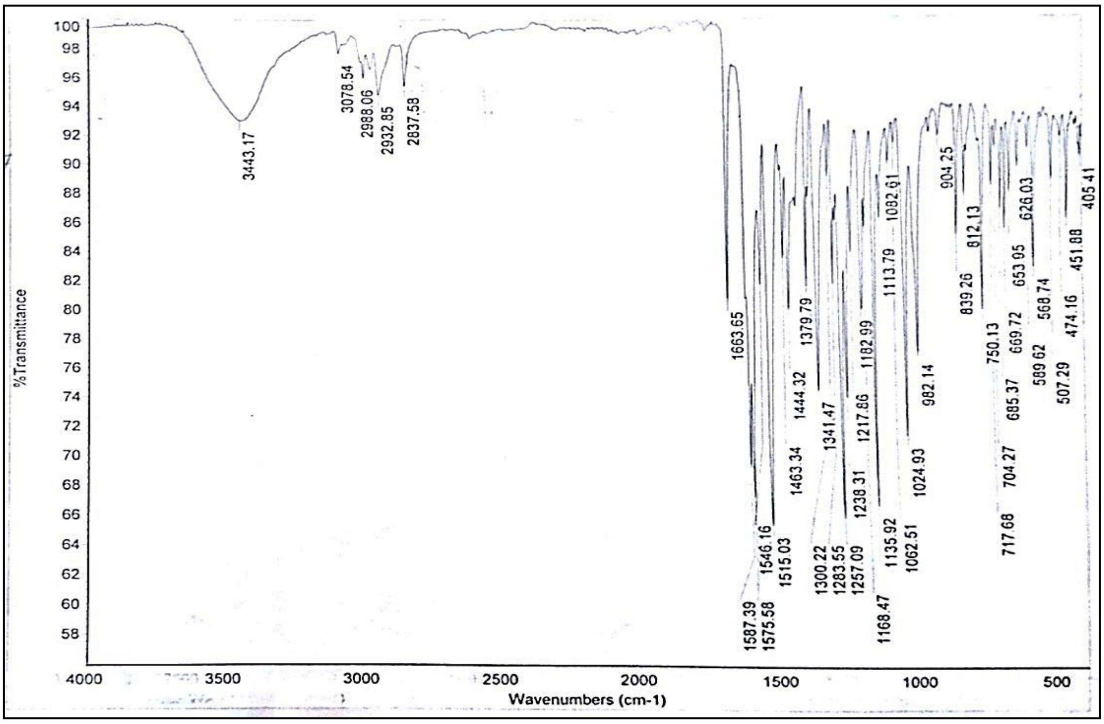

(Fig. 91), *IR spectrum of 3,6-dimethyl-5-(3,4-dimethoxybenzylideneacetyl)-1-phenyl-1H-pyrazolo[3,4-b] pyrazine (**25j**).*

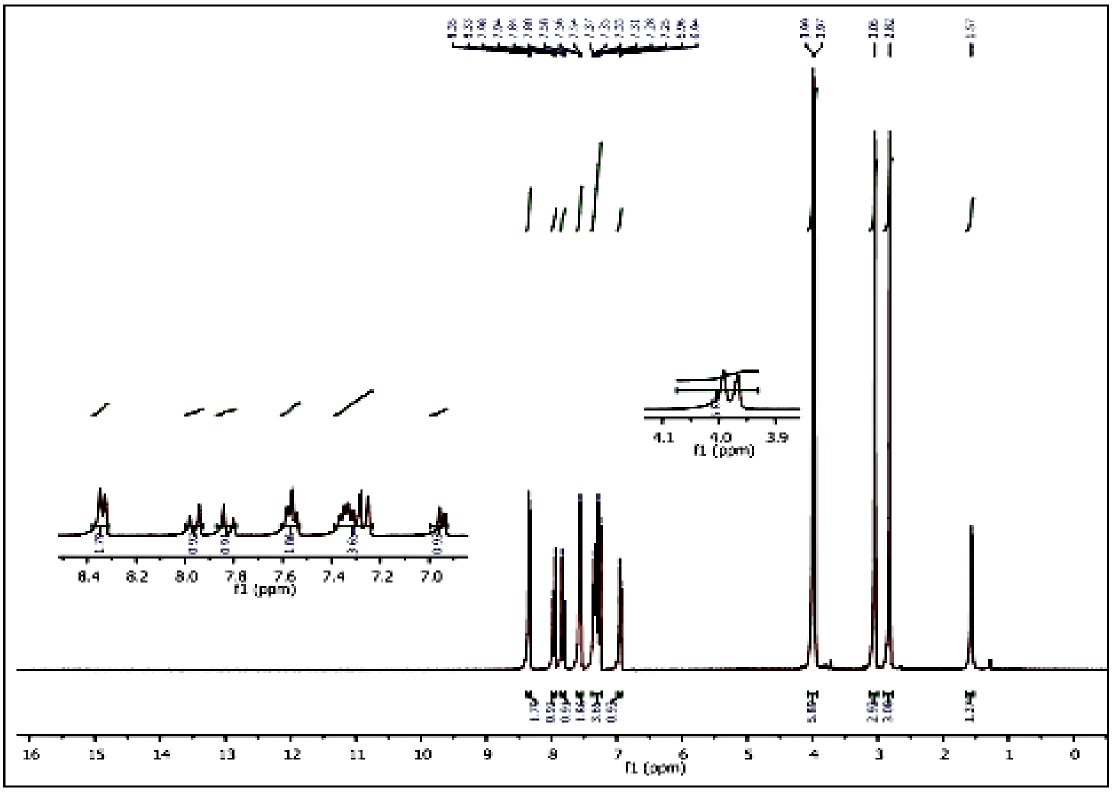

(Fig. 92), *^{1}H-NMR spectrum of 3,6-dimethyl-5-(3,4-dimethoxybenzylideneacetyl)-1-phenyl-1H-pyrazolo[3,4-b]pyrazine (**25j**).$CDCl_3$.*

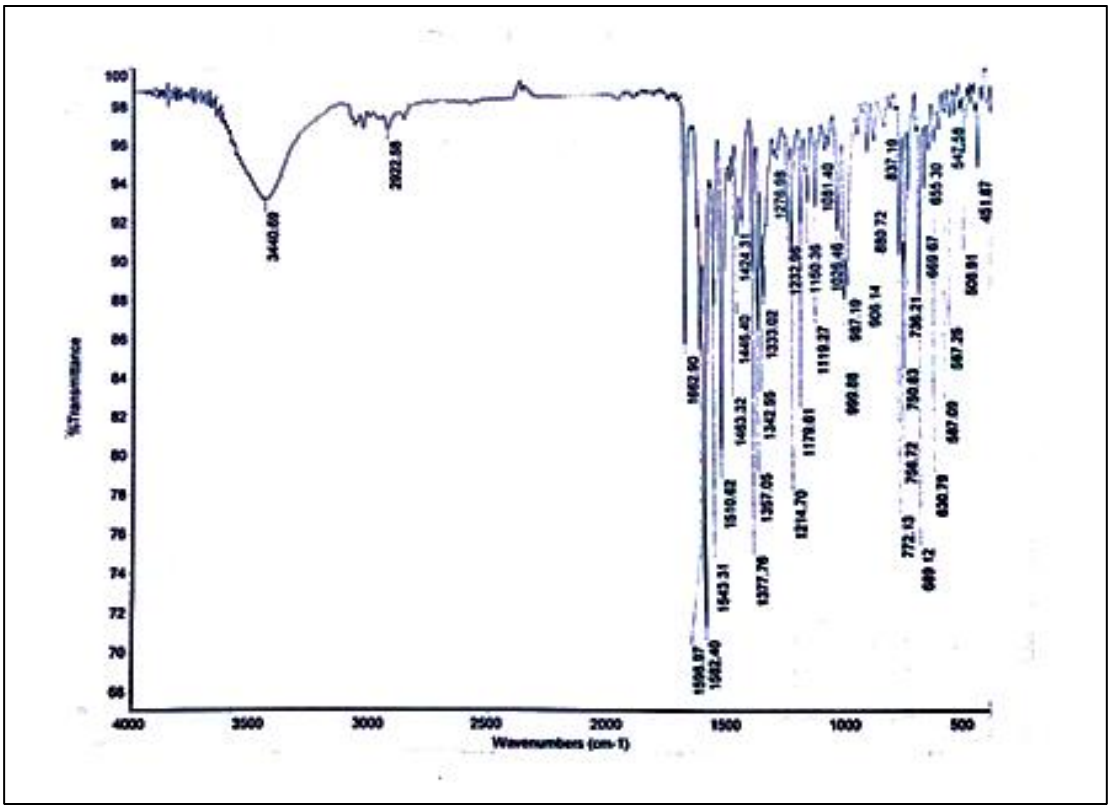

(Fig. 93), *IR spectrum of 3,6-dimethyl-5-(ethene-2-ylbenzylideneacetyl)-1-phenyl-1H-pyrazolo[3,4-b]pyrazine* ***(25k)****.*

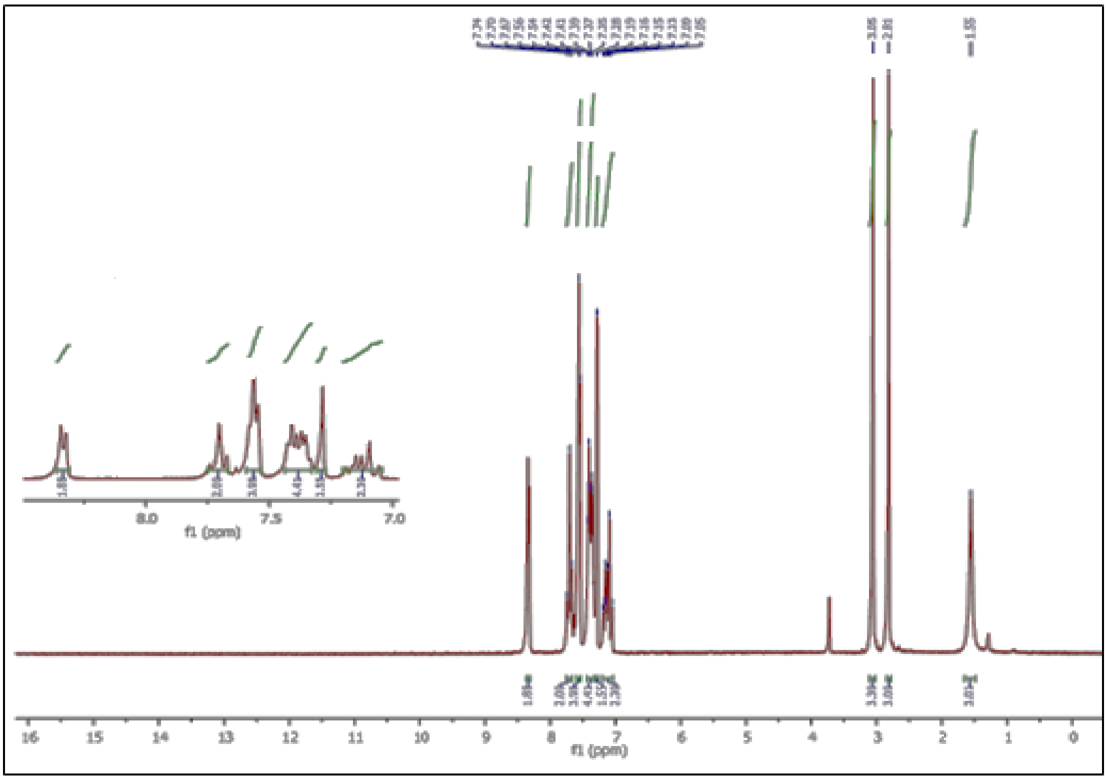

(Fig. 94), *^{1}H-NMR spectrum of 3,6-dimethyl-5-(ethene-2-ylbenzylideneacetyl)-1-phenyl-1H-pyrazolo[3,4-b]pyrazine* ***(25k)****.CDCl$_3$*

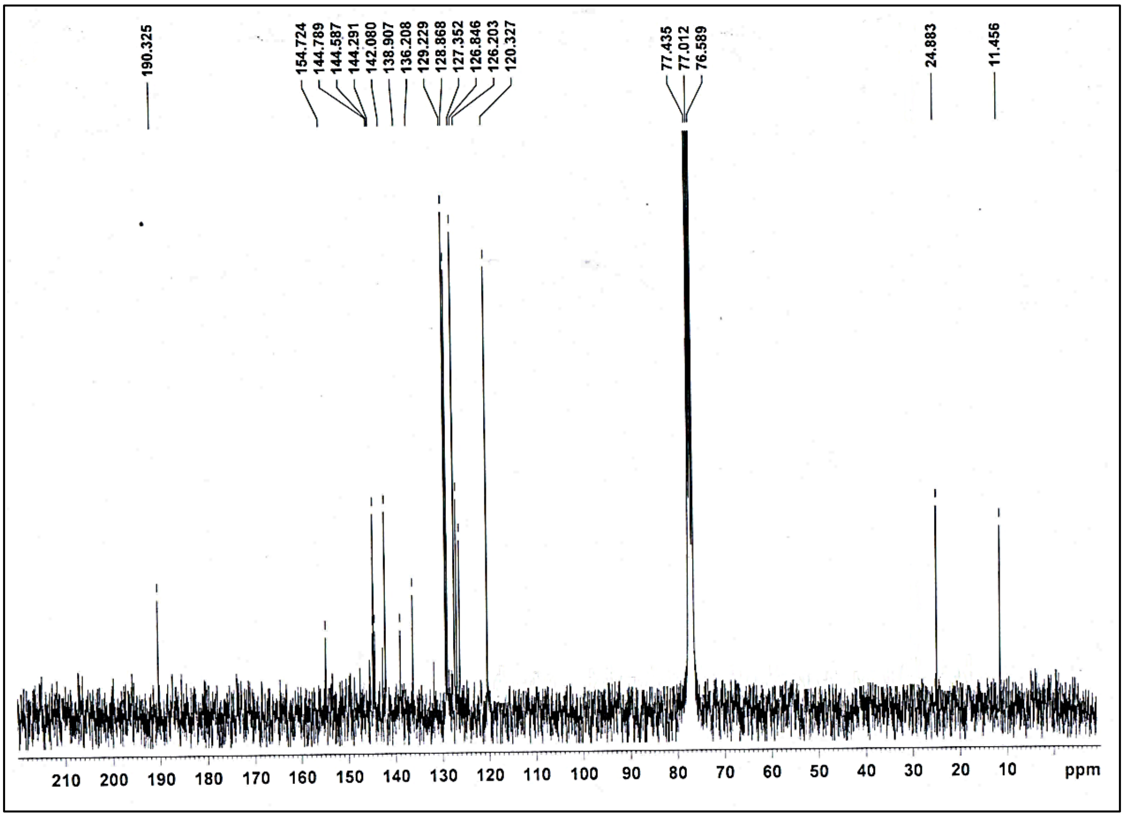

(Fig. 95), *^{13}C-NMR spectrum of 3,6-dimethyl-5-(ethene-2-ylbenzylideneacetyl)-1-phenyl-1H-pyrazolo[3,4-b]pyrazine (**25k**).$CDCl_3$*

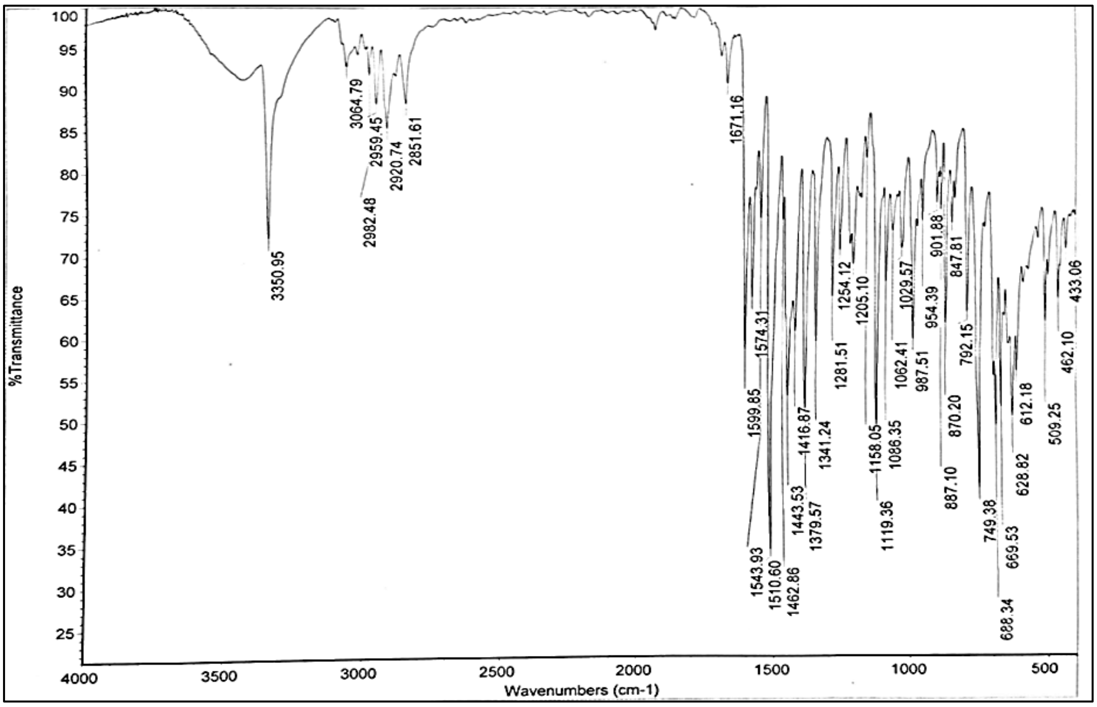

(Fig. 96), *IR spectrum of 3,6-dimethyl-5-(5-phenyl-4,5-dihydro-1H-pyrazol-3-yl)-1-phenyl-1H-pyrazolo[3,4-b]pyrazine (**26**).*

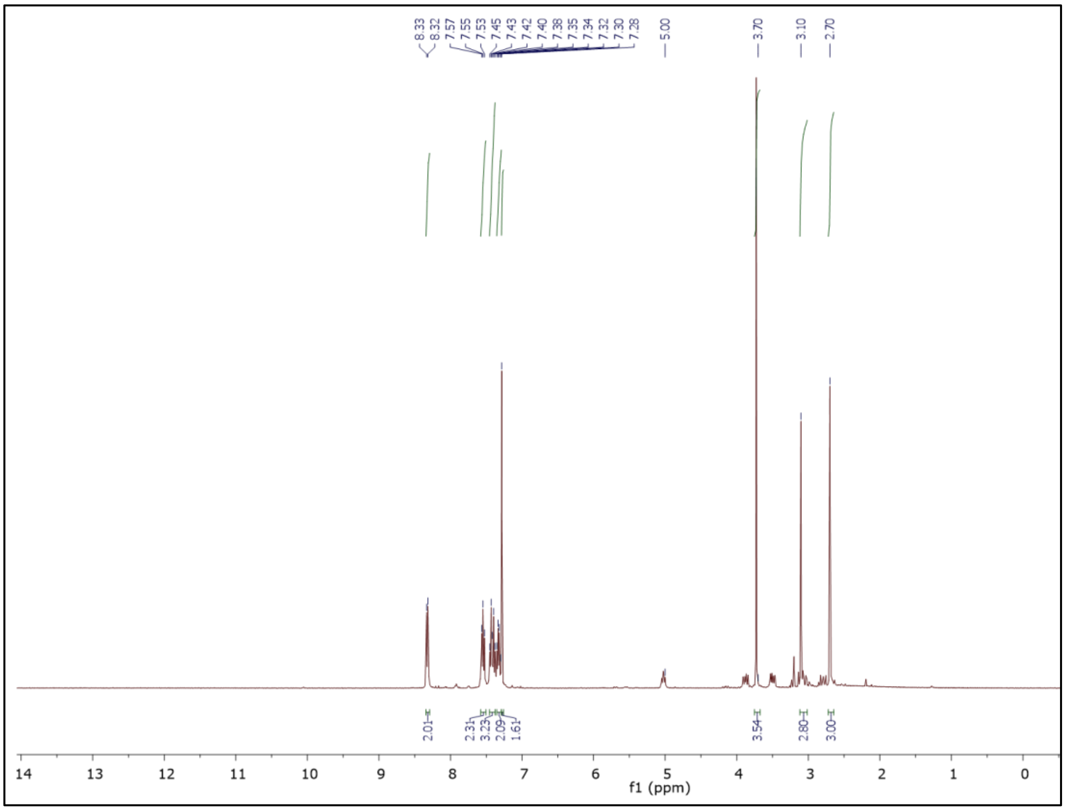

(Fig. 97), *^{1}H-NMR spectrum of 3,6-dimethyl-5-(5-phenyl-4,5-dihydro-1H-pyrazol-3-yl)-1-phenyl-1H-pyrazolo[3,4-b] pyrazine (**26**). $CDCl_3$*

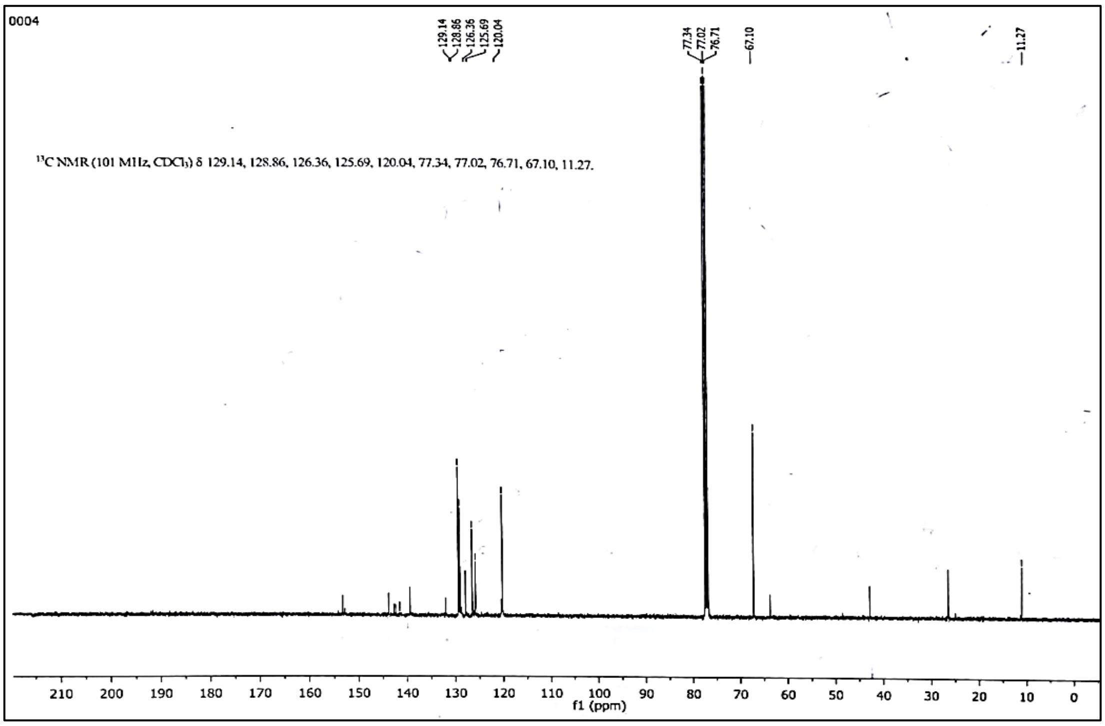

(Fig. 98), *^{13}C-NMR spectrum of 3,6-dimethyl-5-(5-phenyl-4,5-dihydro-1H-pyrazol-3-yl)-1-phenyl-1H-pyrazolo[3,4-b]pyrazine (**26**). $CDCl_3$*

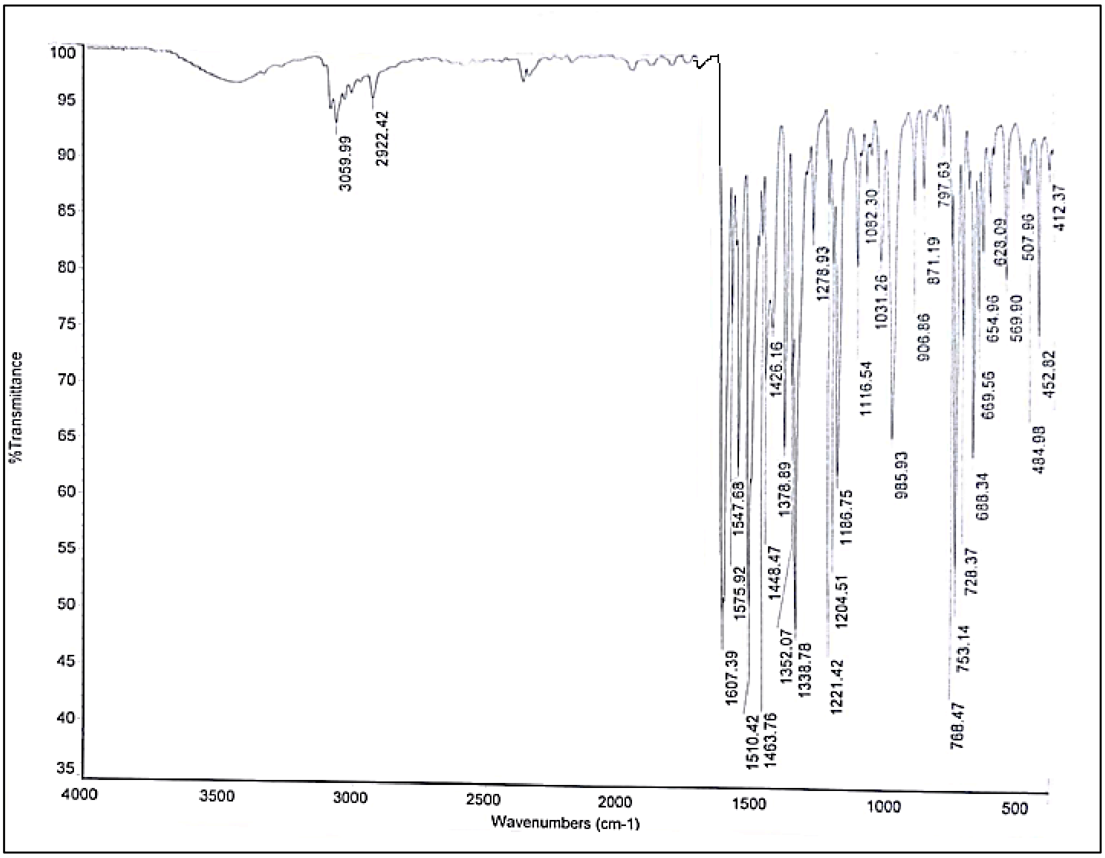

(Fig. 99), *IR spectrum of 3,6-dimethyl-5-(5-phenyl-4,5-dihydro-1-phenyl-1H-pyrazol-3-yl)-1-phenyl-1H-pyrazolo[3,4-b]pyrazine (**27**).*

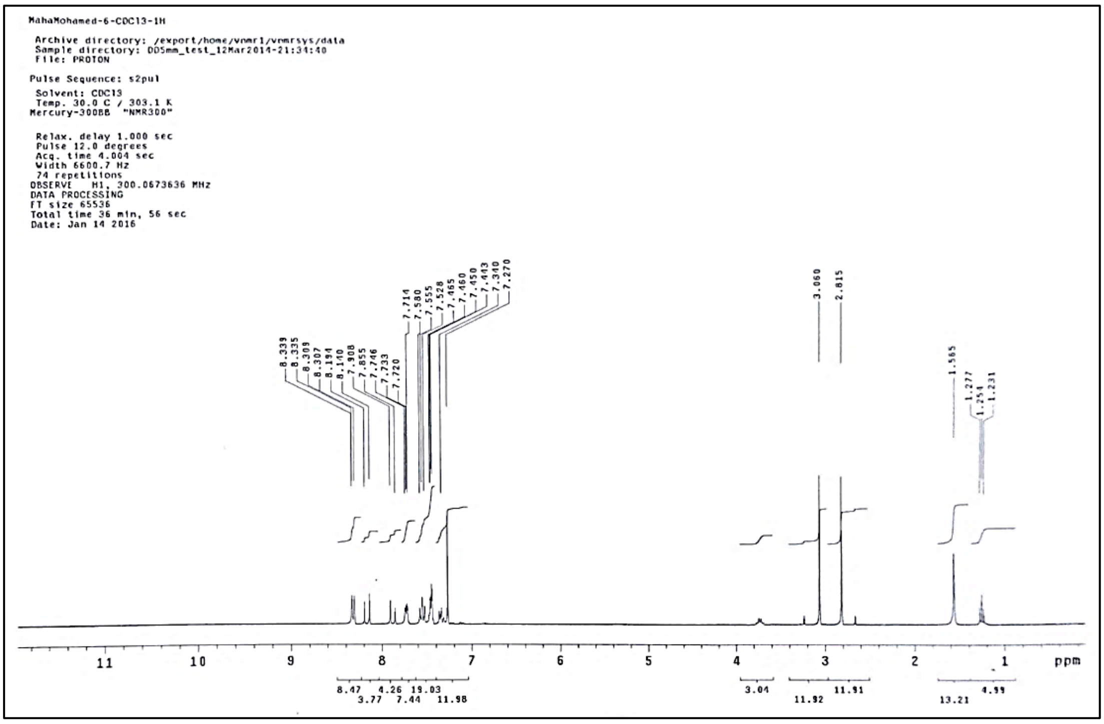

(Fig. 100), *^{1}H-NMR spectrum of 3,6-dimethyl-5-(5-phenyl-4,5-dihydro-1-phenyl-1H-pyrazol-3-yl)-1-phenyl-1H-pyrazolo[3,4-b]pyrazine (**27**).CDCl*$_3$

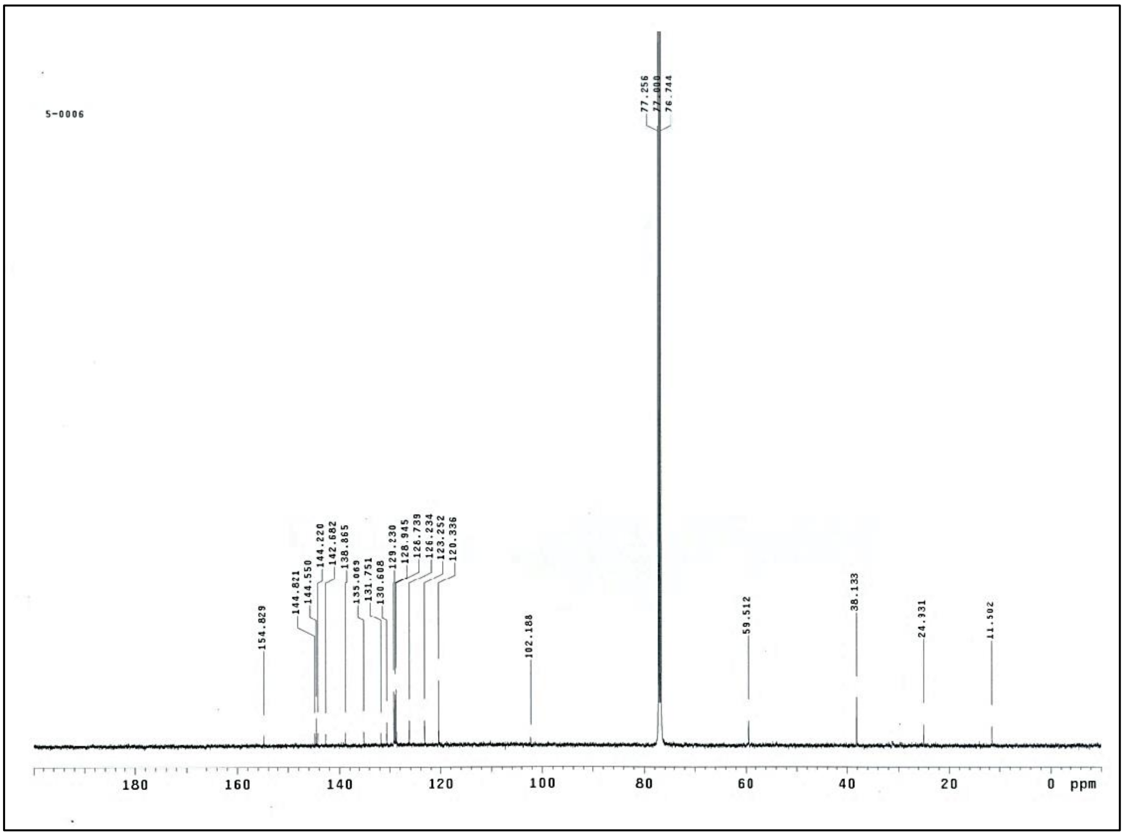

(Fig. 101), *^{13}C-NMR spectrum of 3,6-dimethyl-5-(5-phenyl-4,5-dihydro-1-phenyl-1H-pyrazol-3-yl)-1-phenyl-1H-pyrazolo[3,4-b]pyrazine (**27**).$CDCl_3$*

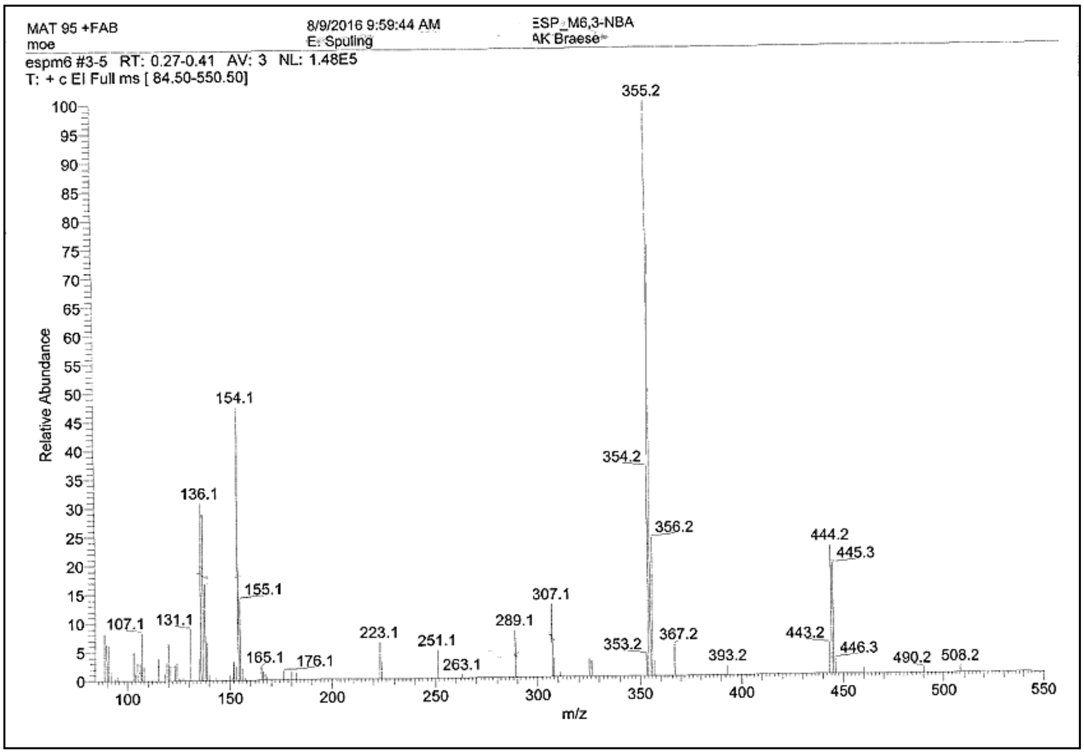

(Fig. 102), *Mass spectrum of 3,6-dimethyl-5-(5-phenyl-4,5-dihydro-1-phenyl-1H-pyrazol-3-yl)-1-phenyl-1H-pyrazolo[3,4-b]pyrazine (**27**).*

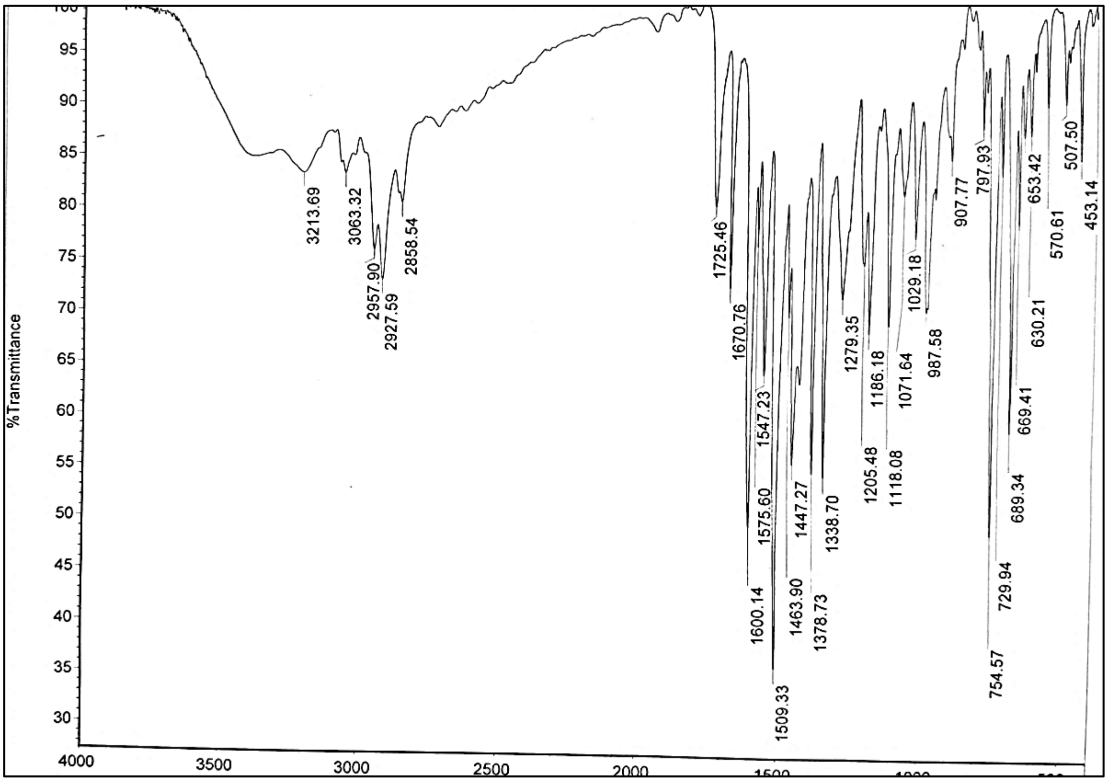

(Fig. 103), *IR spectrum of 3,6-dimethyl-5-(5-phenyl-4,5-dihydro[1,2]oxazol-3-yl)-1-phenyl-1H-pyrazolo[3,4-b]pyrazine (**28**).*

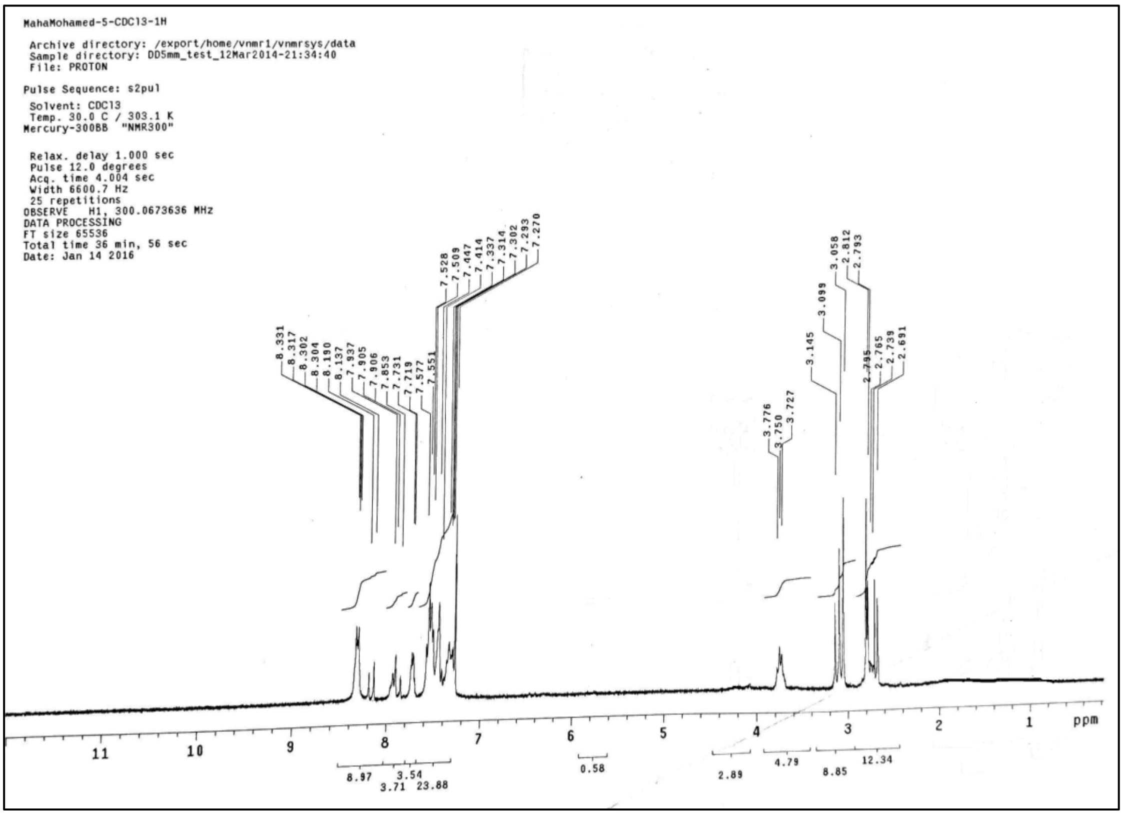

(Fig. 104), *^{1}H -NMR spectrum of 3,6-dimethyl-5-(5-phenyl-4,5-dihydro[1,2]oxazol-3-yl)-1-phenyl-1H-pyrazolo[3,4-b]pyrazine (**28**).$CDCl_3$*

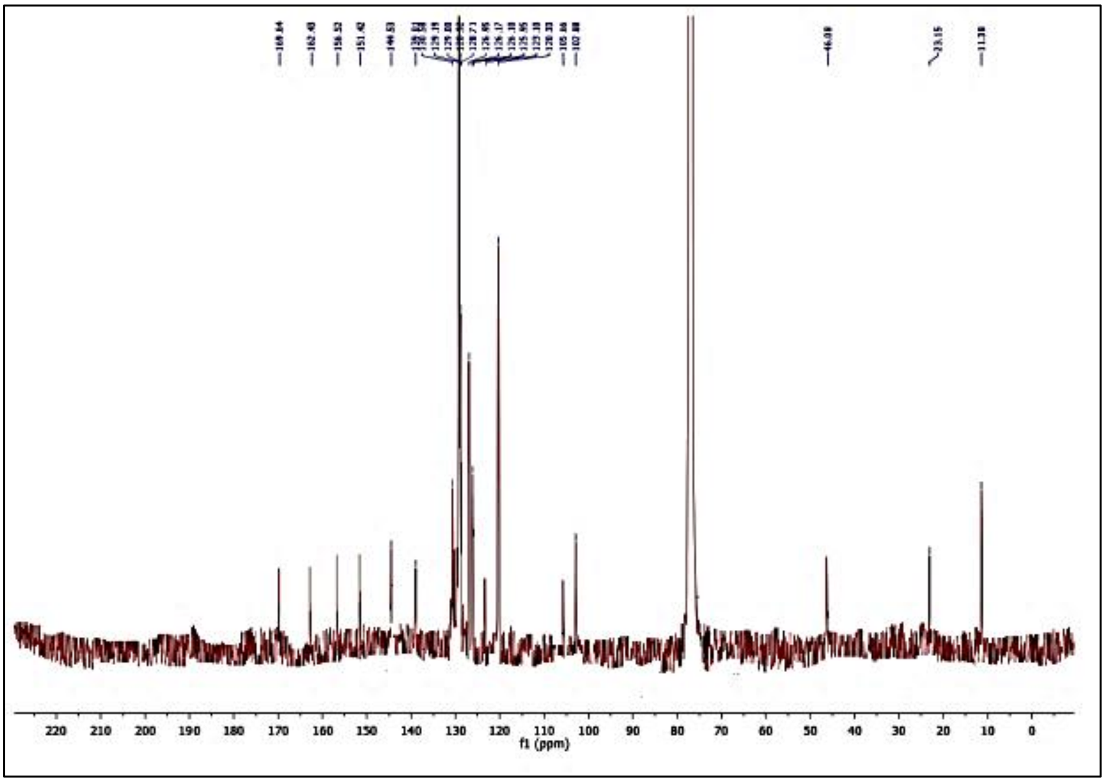

(Fig. 105), *^{13}C-NMR spectrum of 3,6-dimethyl-5-(5-phenyl-4,5-dihydro[1,2]oxazol-3-yl)-1-phenyl-1H-pyrazolo[3,4-b]pyrazine (**28**).$CDCl_3$*

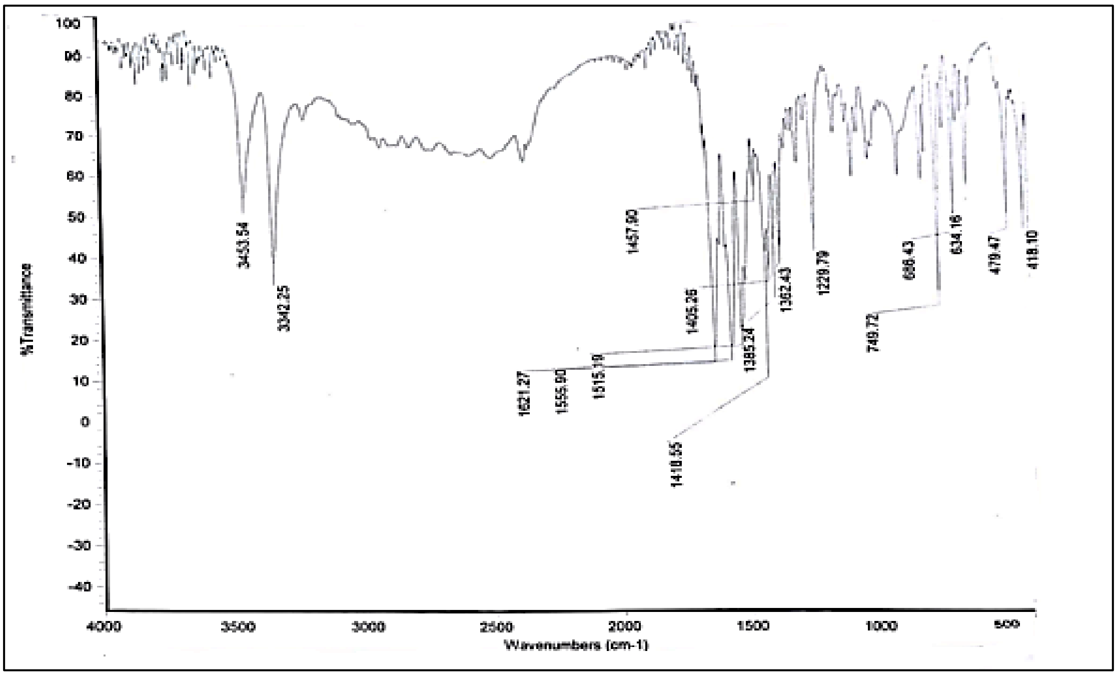

(Fig. 106), *IR spectrum of* 5-phenyl-4,5-dihydro-3-(3,6-dimethyl-1-phenyl-1*H*-pyrazolo[3,4-*b*]pyrazine-5-yl)pyrazole-1-carbothioamide *(29)*

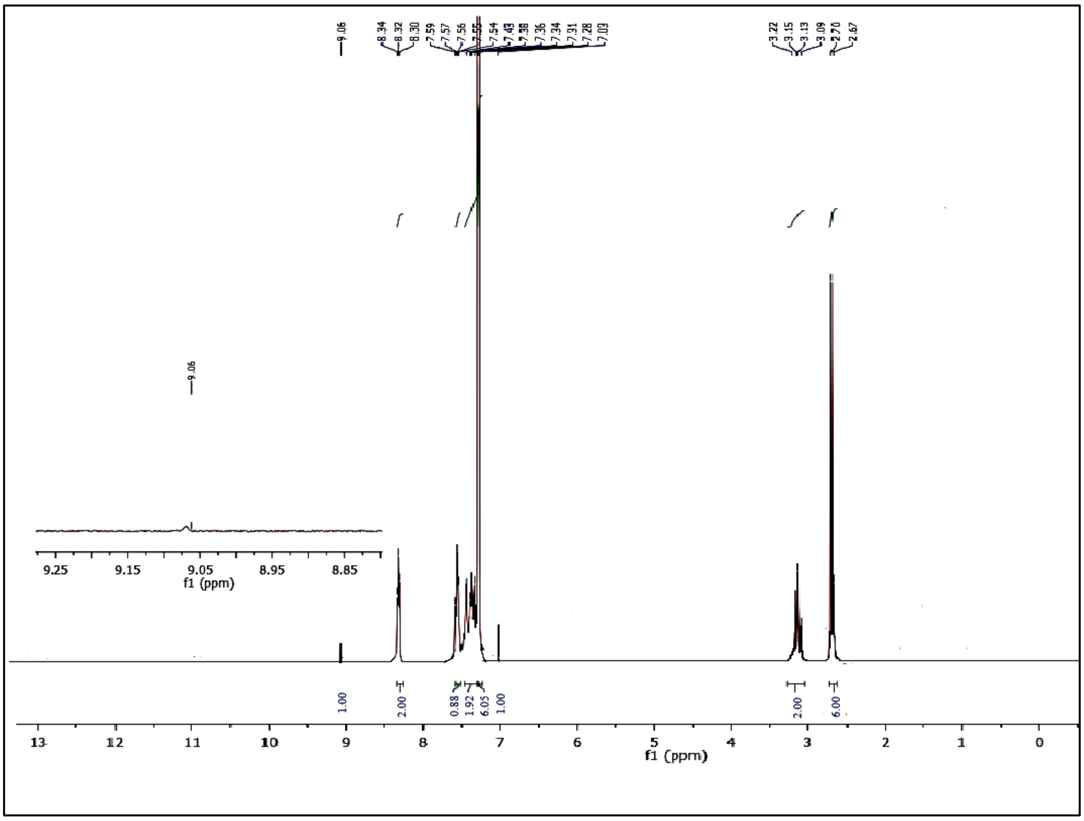

(Fig. 107), *^{1}H-NMRspectrum of* 5-phenyl-4,5-dihydro-3-(3,6-dimethyl-1-phenyl-1*H*-pyrazolo[3,4-*b*]pyrazine-5-yl)pyrazole-1-carbothioamide *(29).CDCl*$_3$

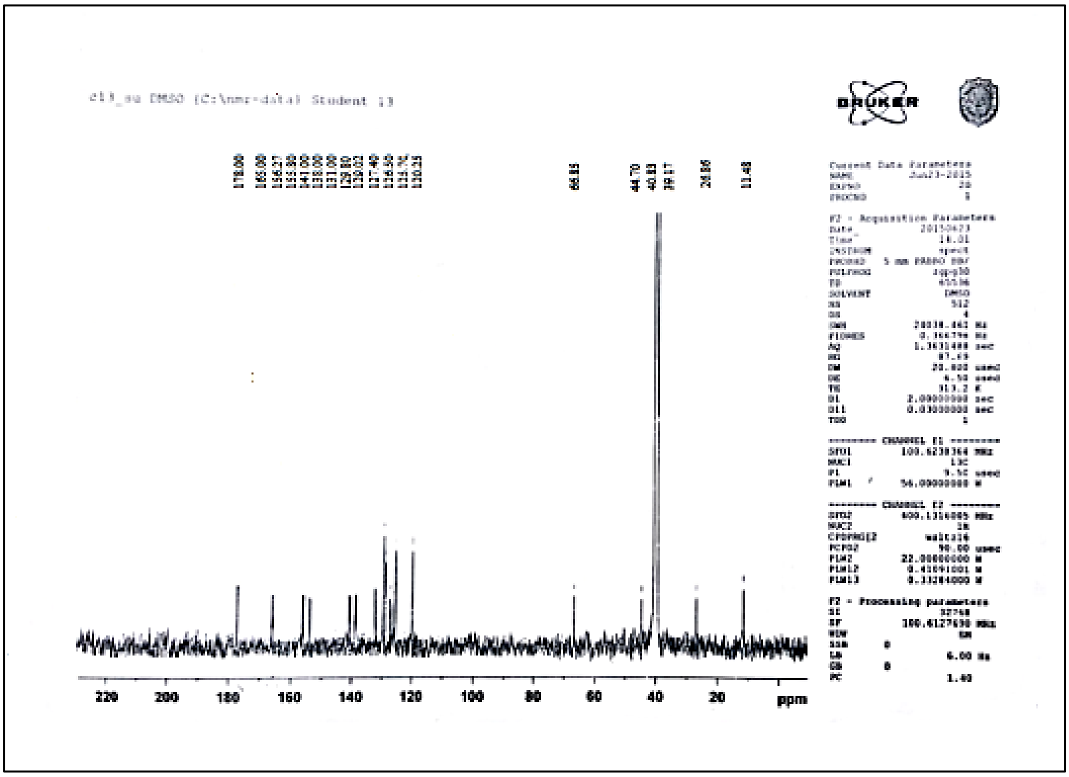

(Fig. 108), *^{13}C-NMRspectrum of* 5-phenyl-4,5-dihydro-3-(3,6-dimethyl-1-phenyl-1*H*-pyrazolo[3,4-*b*]pyrazine-5-yl)pyrazole-1-carbothioamide *(29).CDCl*$_3$

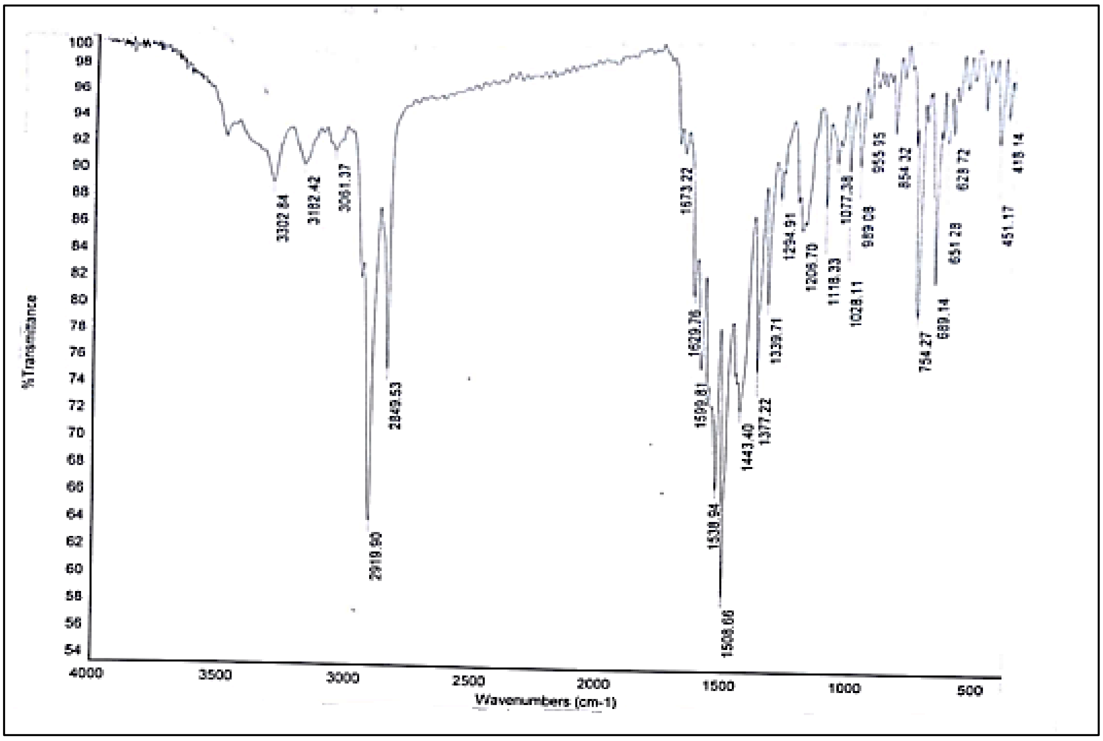

(Fig. 109), *IR spectrum of 2-amino-4-(4-phenyl)-6-(3,6-dimethyl-1-phenyl-1H-pyrazolo[3,4-b]pyrazine-5-yl)pyrimidine (**30**).*

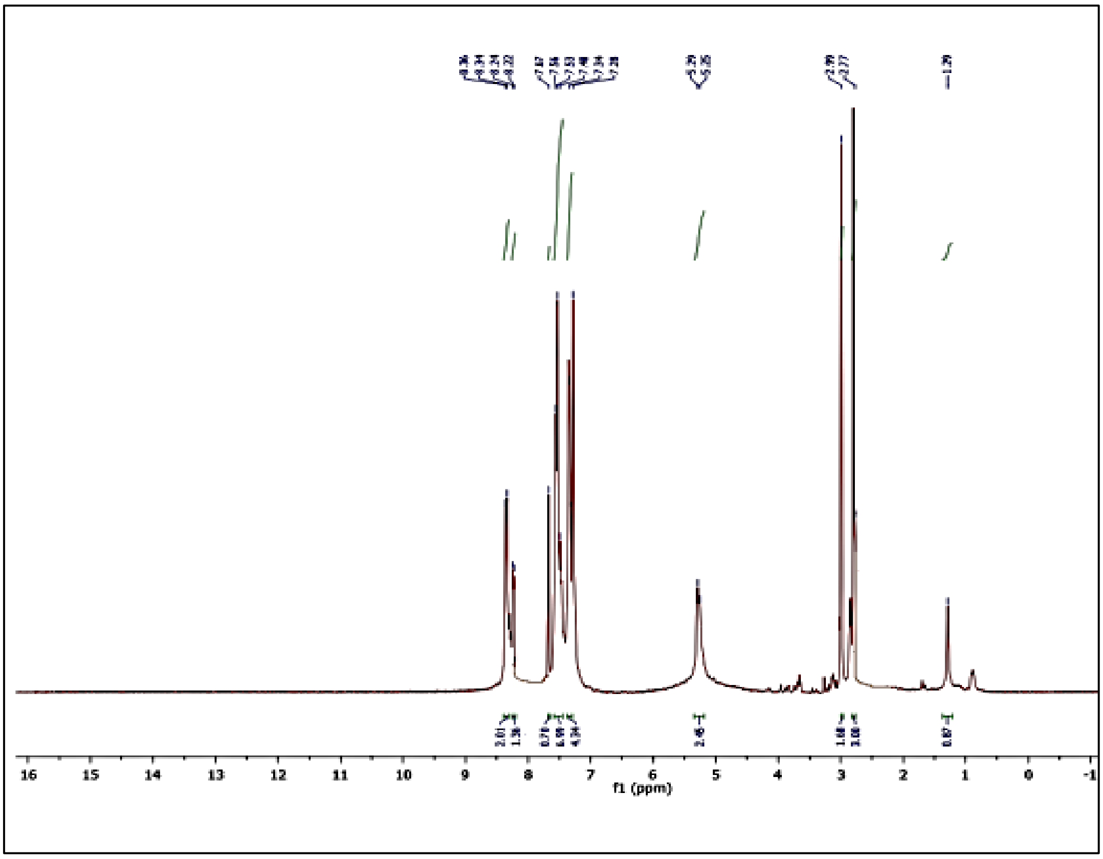

(Fig. 110), *IR spectrum of 2-amino-4-(4-phenyl)-6-(3,6-dimethyl-1-phenyl-1H-pyrazolo[3,4-b]pyrazine-5-yl) pyrimidine (**30**).CDCl*$_3$

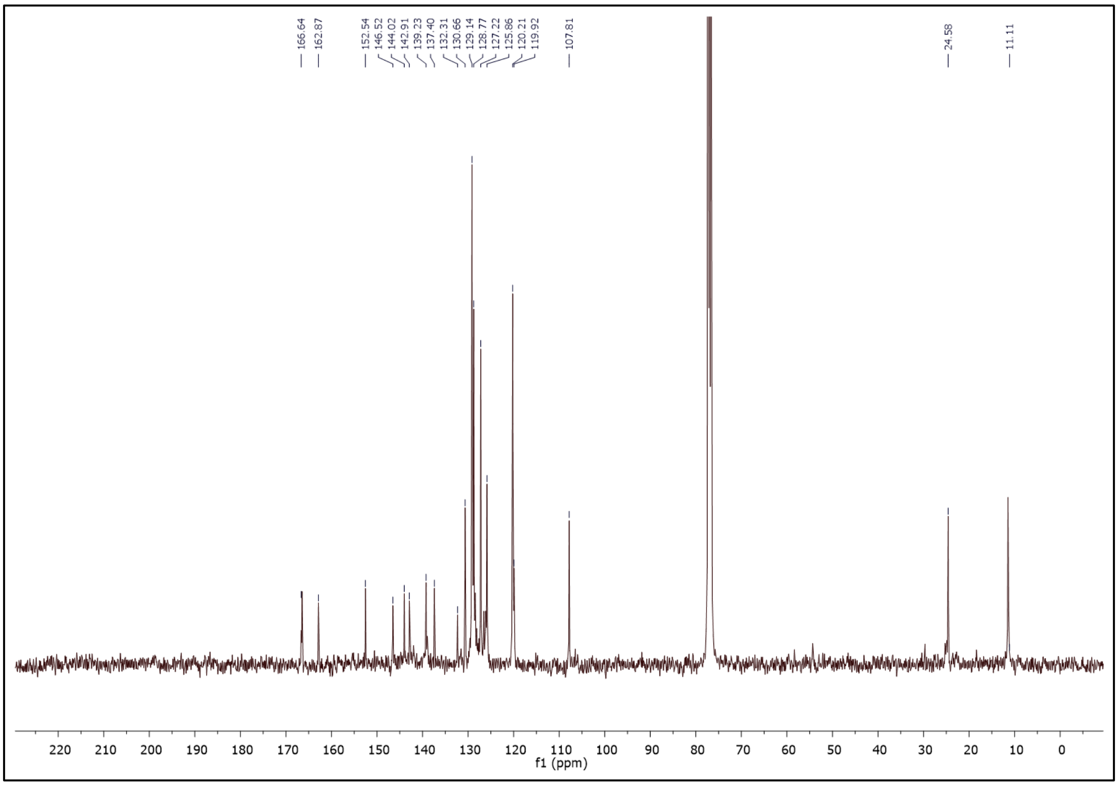

***(Fig. 111),** IR spectrum of 2-amino-4-(4-phenyl)-6-(3,6-dimethyl-1-phenyl-1H-pyrazolo[3,4-b]pyrazine-5-yl) pyrimidine (**30**).CDCl*$_3$
